# The critical role of the endogenous immune compartment after CAR T cell therapy in recurrent GBM

**DOI:** 10.1016/j.cell.2026.05.026

**Published:** 2026-06-15

**Authors:** Nelson F. Freeburg, Daniel Chafamo, Gayathri Konanur Gopikrishna, Regan M. Murphy, Jacqueline J. Peng, Shridhar Parthasarathy, Sydney Dumont, Edward G. Estrada, Meghan T. Logun, Yusha Sun, Xin Wang, Payal Grover, Jesse L. Rodriguez, Daniel L. Zhang, Kristen Park, Yao Fu, Nadine Ben Hamouda, Isaias Hernandez-Verdin, Lamia Lamrani, Kelly A. Hicks, Natalie A. Cooper, Christina Ekwegbara, Emma Grace Bawden, Joshua J. Waterfall, Jaime Fuentealba, Marion Alcantara, John T. Seykora, Stephen M. Prouty, David Barrett, Esha Banerjee, Arin Cox, Charles-Antoine Assenmacher, Camilla Macia, Melinda Yin, Erica L. Carpenter, Guo-li Ming, Catherine Sautès-Fridman, Wolf H. Fridman, Eric Tartour, E. John Wherry, Sebastian Amigorena, Joseph A. Fraietta, MacLean P. Nasrallah, Hongjun Song, Tyler E. Miller, Stephen J. Bagley, Donald M. O’Rourke, Zev A. Binder, Cécile Alanio, Dana Silverbush

**Affiliations:** 1 Cancer Biology Department, Perelman School of Medicine at the University of Pennsylvania, Philadelphia, PA 19104, USA; 2 Department of Pathology, Case Western Reserve University School of Medicine and Case Comprehensive Cancer Center, Cleveland, OH, USA; 3 Department of Neuroscience and Mahoney Institute for Neurosciences, Perelman School of Medicine at the University of Pennsylvania, Philadelphia, PA, USA; 4 Department of Neurosurgery, Perelman School of Medicine at the University of Pennsylvania, Philadelphia, PA 19104, USA; 5 Center for Cellular Immunotherapies, Perelman School of Medicine at the University of Pennsylvania, Philadelphia, PA 19104, USA; 6 GBM Translational Center of Excellence, Abramson Cancer Center, Perelman School of Medicine at the University of Pennsylvania, Philadelphia, PA 19104, USA; 7 Cell and Molecular Biology Graduate Group, Perelman School of Medicine at the University of Pennsylvania, Philadelphia, PA, USA; 8 Neuroscience Graduate Group, Perelman School of Medicine at the University of Pennsylvania, Philadelphia, PA, USA; 9 Department of Biology, School of Arts and Sciences, University of Pennsylvania, Philadelphia, PA, USA; 10 Université Paris Cité, INSERM, PARCC, Paris, France; 11 Department of Immunology, APHP, Hôpital Européen Georges Pompidou (HEGP)-Hôpital Necker, Paris, France; 12 Centre de Recherche des Cordeliers, Sorbonne Université, INSERM, Université Paris Cité, 75006 Paris, France; 13 CellAction, Center for Cancer Immunotherapy, INSERM U932, Institut Curie, Saint-Cloud, France; 14 Clinical Immunology Laboratory, Institut Curie, Paris, France; 15 Institut Curie, PSL University, INSERM U932, Immunity and Cancer, 75005 Paris, France; 16 Department of Translational Research, PSL University, Institut Curie, Paris, France; 17 INSERM U1330, PSL University, Institut Curie Research Center, Paris, France; 18 Clinical Hematology Unit, Institut Curie, Saint-Cloud, France; 19 Departments of Dermatology and Pathology, Perelman School of Medicine at the University of Pennsylvania, Philadelphia, PA, USA; 20 Department of Dermatology, Perelman School of Medicine at the University of Pennsylvania, Philadelphia, PA 19104, USA; 21 Kite, a Gilead Company, Santa Monica, CA, USA; 22 Comparative Pathology Core, Department of Pathobiology, University of Pennsylvania, School of Veterinary Medicine, Philadelphia, PA, USA; 23 Department of Medicine, Division of Hematology and Oncology, Perelman School of Medicine at the University of Pennsylvania, Philadelphia, PA, USA; 24 Department of Cell and Developmental Biology, Perelman School of Medicine at the University of Pennsylvania, Philadelphia, PA, USA; 25 Department of Psychiatry, Perelman School of Medicine at the University of Pennsylvania, Philadelphia, PA, USA; 26 Institute for Regenerative Medicine, University of Pennsylvania, Philadelphia, PA, USA; 27 Équipe labellisée Ligue Contre le Cancer, Centre de Recherche des Cordeliers, 15 rue de l’école de médecine, 75006 Paris, France; 28 Department of Systems Pharmacology and Translational Therapeutics, Perelman School of Medicine at the University of Pennsylvania, Philadelphia, PA, USA; 29 Institute for Immunology and Immune Health, Perelman School of Medicine at the University of Pennsylvania, Philadelphia, PA, USA; 30 Department of Microbiology, Perelman School of Medicine at the University of Pennsylvania, Philadelphia, PA, USA; 31 Department of Pathology and Laboratory Medicine, Perelman School of Medicine at the University of Pennsylvania, Philadelphia, PA, USA; 32 Parker Institute for Cancer Immunotherapy, University of Pennsylvania, Philadelphia, PA, USA; 33 Epigenetics Institute, Perelman School of Medicine at the University of Pennsylvania, Philadelphia, PA, USA; 34 Department of Pathology, University Hospitals Cleveland Medical Center, Cleveland, OH, USA; 35 Clinical Laboratory, Hôpital Foch, Suresnes, France; 36 These authors contributed equally; 37 Senior author; 38 Lead contact

## Abstract

Glioblastoma (GBM) is the most common primary malignant brain tumor in adults, with a median survival of under 15 months and no effective treatment after recurrence. A recent phase 1 trial of intracerebroventricular bivalent chimeric antigen receptor (CAR) T cells in recurrent GBM, registered at ClinicalTrials.gov (NCT05168423), showed promising responses, including tumor reduction and prolonged survival. However, relapse remains common. We performed in-depth profiling of longitudinal cerebrospinal fluid (CSF) and tumor samples from responders and non-responders to characterize immune dynamics following infusion. Our study reveals that, although CAR T cells activate post infusion across all patients, outcomes were defined by divergent remodeling of the endogenous immune landscape. Cytotoxic natural killer cell expansion characterized responders, whereas regulatory T cell expansion and abundant baseline immunosuppressive scavenger myeloid cells characterized non-responders. These findings indicate that host immune cells play a critical role in CAR T cell therapy for GBM, suggesting that combinatorial strategies modulating the endogenous immune compartment could improve next-generation treatments.

## In brief

Single intracerebroventricular CAR T cell dose in patients with recurrent glioblastoma induces dose-dependent endogenous immune remodeling. Cytotoxic NK cell expansion is associated with prolonged survival, whereas Treg expansion and baseline scavenger myeloid cells are associated with poor outcomes.

## Graphical abstract

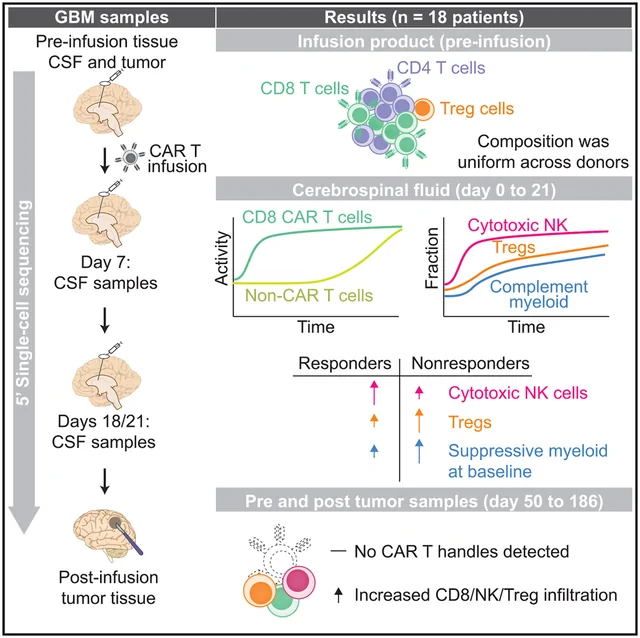

## INTRODUCTION

Although chimeric antigen receptor (CAR) T cell therapy has revolutionized the treatment of hematologic malignancies,^1,2^ translating this success to solid tumors has proven challenging.^3,4^ Key barriers include antigen paucity, inefficient T cell trafficking, tumor heterogeneity, antigen loss, and the immunosuppressive tumor microenvironment (TME).^5 ,6^ Glioblastoma (GBM), the most common and lethal primary malignant brain tumor in adults, exemplifies these obstacles, particularly regarding therapeutic resistance and immune evasion.^7^ Despite aggressive standard-of-care consisting of resection, radiotherapy, and temozolomide,^8^ progression typically occurs within 1 year of diagnosis. Overall survival (OS) remains poor—less than 15 months for newly diagnosed patients and 6–10 months for recurrent disease—with no meaningful therapeutic improvement since 2005.^9–13^

Recently, locoregional and intracerebroventricular (ICV) delivery of CAR T cells has demonstrated encouraging efficacy, marking a major advance in neuro-oncology.^14–18^ For example, IL13Rα2 CAR T cells mediated durable radiographic responses in a subset of patients with recurrent high-grade glioma,^15^ while GD2 CAR T cells (delivered intravenously and subsequently intracranially) led to tumor regression and neurological improvement in H3K27M-mutant diffuse midline gliomas.^19,20^ Similarly, ICV-delivered B7-H3 CAR T cells showed tolerability and efficacy signals in diffuse intrinsic pontine glioma (DIPG),^21,22^ and EGFRvIII CAR T cells secreting EGFR-directed T cell-engaging antibodies yielded dramatic radiographic responses in an early-phase trial for recurrent GBM (rGBM).^16^ Finally, a phase 1 study of bivalent EGFR-IL13Rα2 CAR T cells at the University of Pennsylvania treated 18 patients with rGBM; among those with measurable disease, 62% achieved tumor regression lasting ≥1 month, and 28% of the total cohort achieved disease control lasting ≥4 months, including one ongoing case exceeding 16 months.^14^

Although representing important progress, these studies underscore a critical limitation: many patients fail to respond, and benefits are often transient. Understanding the mechanisms underlying response and resistance is therefore crucial for optimizing future iterations.

Much of what is known about CAR T cell response and resistance is derived from hematological malignancies,^23,24^ where factors impacting response include apheresis^25^ or infusion product (IP)^25,26^ composition, immune checkpoints (e.g., TIGIT^27^), immunosuppressive myeloid cells,^28^ and the overall immune context.^29^ However, it remains unclear whether these same factors govern outcomes in GBM. Key questions regarding CAR T cell trafficking after infusion, their phenotypic and functional evolution within the CNS, mechanisms of persistence, and their impact on the broader immune landscape remain unresolved.

Investigating these dynamics in solid tumors is hindered by limited access to the TME. Blood-based analyses fail to capture intratumoral processes, and serial tumor sampling is rarely feasible. Although cerebrospinal fluid (CSF) has been utilized for quantifying cell-free DNA, cytokine profiling, and immune repertoire analysis,^14,30^ its inherently low cellularity has historically restricted its use for high-resolution cellular profiling. To overcome these challenges, we applied single-cell RNA sequencing (scRNA-seq) to 62 samples from 18 patients, integrating serial CSF sampled via an indwelling Ommaya reservoir, matched pre- and post-treatment tumor tissue, and CAR T cell IPs. Patients were stratified into high-dose (*n* = 12) and low-dose (*n* = 6) cohorts, with the high-dose group structured into a discovery set to identify longitudinal immune correlates and a validation set to confirm them. This dataset offers a high-resolution, integrated view of immune modulation and tumor evolution.

Here, we show that CAR T cells activated post infusion and peaked near day 7, triggering a dose-dependent remodeling of the endogenous immune compartment that was reproduced across the discovery and validation cohorts. Clinical outcomes were associated with the endogenous immune compartment rather than CAR T kinetics. Responders exhibited an expansion of cytotoxic natural killer (NK) cells, whereas non-responders showed regulatory T cell (Treg) expansion and a high baseline burden of immunosuppressive scavenger myeloid cells. These findings identify host immune programs that may be targeted to improve ICV CAR T therapy in GBM.

## RESULTS

### CSF immune landscape after ICV CAR T cell therapy

**Figure 1.**
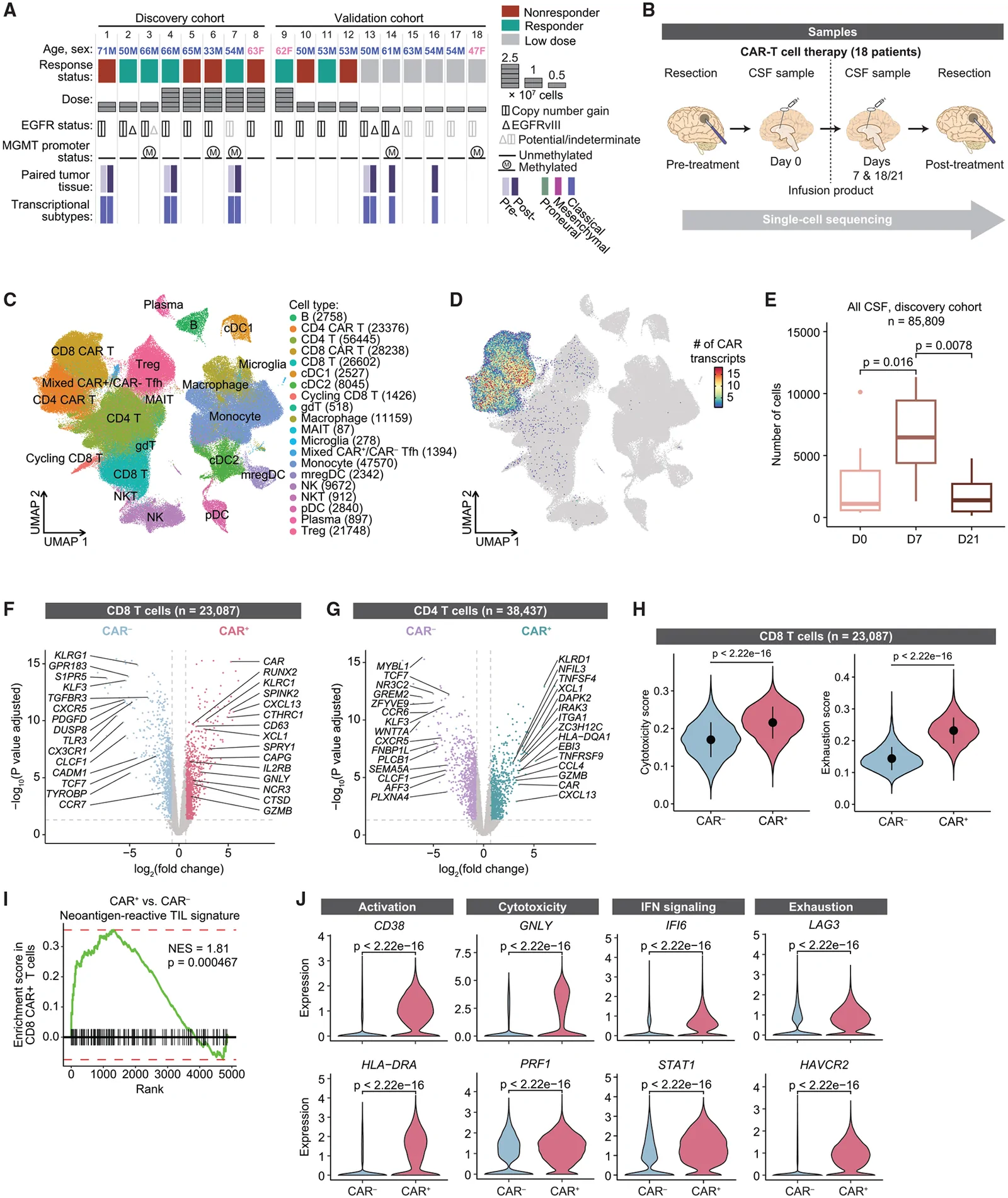
CSF immune landscape after ICV CAR T cell therapy (A) Patient clinical characteristics. (B) Sample collection timeline. (C and D) UMAP of full cohort CSF cells from 18 patients (*n* = 248,834 cells) annotated by (C) cell type and (D) CAR transcript count. (E) Total CSF cell recovery per patient by day (*n* = 8 patients, discovery cohort). (F and G) Differential gene expression between CAR+ and CAR− clusters in the discovery cohort within (F) CD8 T cells (*n* = 23,087) and (G) CD4 T cells (*n* = 38,437). (H) Cytotoxicity and exhaustion scores of CD8 T cells (*n* = 23,087) in CAR+ vs. CAR− clusters in the discovery cohort. (I and J) In CD8 T cells, (I) gene set enrichment analysis (GSEA) of the neoantigen-reactive TIL signature and (J) expression of selected genes comparing CAR+ vs. CAR− clusters (*n* = 23,087, discovery cohort). Data in (E) represent median (center), IQR (box), and 1.5× IQR (whiskers). In (H), black dots and vertical lines denote mean ± SD. *p* values: two-sided paired Wilcoxon signed-rank test (E), quasi-likelihood F test (F and G), or Wilcoxon rank-sum test (H and J). See also Figure S1.

Here, we describe the CSF and tumor immune landscape for all 18 patients from a phase 1 study of bivalent CAR T-EGFR-IL13Rα2 cells in EGFR-amplified rGBM (study design and eligibility published previously^14^). To enable discovery and confirmation of immune correlates, patients were stratified into three cohorts based on cell dose and sample availability (Figure 1A; Table 1). Deep longitudinal profiling of the high-dose discovery cohort identified early immune changes as key correlates of response (day 0 to day 7), which were subsequently tested in the high-dose validation and low-dose cohorts. Finally, to capture intratumoral dynamics, we analyzed paired pre- and post-treatment tissues from six patients requiring repeat resection. This integrated dataset allowed for a high-resolution analysis of CSF kinetics, IP characteristics, and the TME.

Clinically, the trial identified responders and non-responders based on a composite assessment of objective radiographic response, progression-free survival (PFS), and OS (Figures S1A–S1D; Table S1). Among the 12 high-dose patients (discovery + validation), outcomes were evenly distributed (six responders, six non-responders). Responders showed longer PFS (*p* = 0.0022; Figure S1B), greater tumor size reduction (*p* = 0.032; Figure S1C; see Figure 4 and Extended Data Figure 7 in Bagley et al.^14^ for longitudinal MRI series), and improved OS (*p* = 0.065; Figure S1D). The low-dose cohort showed insufficient clinical benefit following the first infusion, marked by reduced CAR T expansion and limited tumor shrinkage^14^; subsequent dosing was not analyzed here. Because baseline clinical features did not explain the variable outcomes in the high-dose cohort,^14^ we turned to granular single-cell profiling of the CSF and tumor tissue to delineate determinants of efficacy.

Unlike the typically acellular CSF of healthy individuals,^30^ the rGBM environment and CAR T cell infusion drastically alter this compartment. To define the kinetics of the infused cells and their impact on endogenous immunity, we longitudinally monitored CAR T cells alongside other immune cells in the CSF of trial participants. Guided by qPCR kinetics showing a day 7 peak followed by decline and persistence through the first month,^14,31,32^ we focused our single-cell analysis on day 0 (pre-infusion), day 7 (peak), and days 18–21 (contraction) (Figure 1B).

Combining data from all 18 patients, we generated a global uniform manifold approximation and projection (UMAP) of all cells circulating in the CSF at these time points (Figure 1C; Data S1). CAR T cells were identified using a CAR transcript (Figure 1D), and cells were annotated using lineage genes (Figure S1E). Clusters of CAR T cells were transcriptionally distinct from clusters not enriched for CAR T cells and were denoted CAR+ (CAR) and CAR− (non-CAR) T cell clusters. Of note, non-CAR T cells may include both non-transduced T cells present in the IP and endogenous T cells recruited to the CSF. The CSF landscape was dominated by CD8 and CD4 T cells (CAR and non-CAR), alongside populations of Tregs, NK cells, myeloid cells, B cells, and plasma cells (Figure S1F).

To quantify longitudinal kinetics, we focused on the discovery cohort. At day 0, the median single-cell recovery was 1,047 [IQR 571–3,706], indicating non-quiescent CSF at baseline (Figure S1G). Day 1 white blood cell counts and neutrophil/lymphocyte proportions did not differ by response status (Figure S1H). By day 7, median recovered cell counts increased to 6,266 [IQR 4,301–9,215] (Figure 1E, *p* = 0.016), before declining to 1,340 [IQR 480–2,598] at day 21 (*p* = 0.00078). Both CAR and non-CAR T cells followed these kinetics (Figures S1I and S1J). This acute expansion was robustly recapitulated in the validation cohort, where cell recovery increased in every patient, driving the median from 1,258 [IQR 134–3,038] at day 0 to 7,631 [IQR 3,855–12,379] at day 7.

To identify key differences between T cells in CAR and non-CAR clusters, we performed differential gene expression analysis on both CD8 and CD4 T cells (Figures 1F and 1G). We did not find a significant difference in the proportion of CD8:CD4 CAR T cells in responders vs. non-responders (Figure S1K). CD8 CAR T cells expressed higher levels of *RUNX2*, *GNLY*, *GZMB*, *IL2RB*, and *NCR3* compared with their non-CAR counterparts, suggesting an effector phenotype, while CD4 CAR T cells expressed higher levels of *KLRD1*, *GZMB*, and *TNFSF4*, indicating cytotoxic potential. Applying predefined cytotoxicity^33^ and exhaustion^34^ signatures confirmed that CD8 CAR T cells expressed higher levels of cytotoxic and exhaustion-related genes than non-CAR CD8 T cells (Figure 1H), consistently across patients, including the validation cohort (Table S2), thereby suggesting functional engagement with CAR targets. In line with this activated state, soluble CD27^35,36^ levels in the CSF increased at day 7 compared with day 0 (Figures S1L and S1M). CD8 CAR T cells were enriched for a neoantigen-reactive CD8 tumor-infiltrating lymphocyte (TIL) signature^37^ post infusion (discovery: Figure 1I, normalized enrichment score [NES] = 1.81, *p* = 4.67 × 10^−4^; validation: NES = 1.66, *p* = 1.43 × 10^−5^), driven by genes such as *GZMB* and *HLA-DR*, further supporting activation upon target engagement (Figure S1N). This suggested that CD8 CAR T cells adopted a chronic-stimulation gene-expression pattern resembling bona fide neoantigen-reactive TILs but via CAR-mediated recognition rather than T cell receptor (TCR)-based activation. Finally, specific gene analysis revealed that CAR+ clusters expressed higher levels of activation (*CD38* and *HLA-DRA*), cytotoxicity (*GNLY* and *PRF1*), recent interferon (IFN) signaling (*IFI6* and *STAT1*), and exhaustion (*LAG3* and *HAVCR2*) markers (Figure 1J; Table S3). Of note, non-CAR T cells also displayed signs of activation, with coordinated expression of *PRF1*, *STAT1*, and *LAG3* (Figure 1J). Together, these data indicated that CAR T cells circulating in the CSF acquired a highly activated effector phenotype.

### IP characteristics and clinical outcome associations

In hematologic malignancies (e.g., chronic lymphocytic leukemia^26^), specific product attributes, such as early memory and low exhaustion, correlate with improved outcomes. To characterize the infusion products (IPs), we used a 30-marker spectral flow cytometry panel (Figure S2A; Data S1) and scRNA-seq (Figures S2B and S2C, discovery cohort, *n* = 79,653 cells). Products were dominated by T helper (Th)1 CD4 (~66%) and CD8 (~25%) T cells, alongside smaller Th2, Th17, and Treg fractions (Figures S2D and S2E). Consistent with our bicistronic vector design, EGFR and IL13Rα2 CARs were expressed at similar levels (Figure S2F) and previously confirmed to co-express.^14^ CARs were detected on a median of 21% of infused CD8 T cells by flow (Figure S2G) and 25%–35% across subtypes by scRNA-seq (Figure S2H). Across modalities, CD8 CAR T cells exhibited an activated, highly proliferative profile (high HLA-DR/HLA-*DRA* and Ki-67/*MKI67*), with elevated LAG-3 but low programmed cell death 1 (PD-1)/*PDCD1*, CTLA-4, and TOX levels (Figures S2I and S2J). Despite this activated profile at infusion, neither broad features (CAR expression and activation; Figures S2G, S2I, and S2J) nor subtler cellular and transcriptional features (Data S1) differed significantly between outcome groups. Thus, within the statistical constraints of this study, variations in product composition did not explain the divergent clinical outcomes.

### Longitudinal evolution of CAR and non-CAR T cells in the CSF post infusion

**Figure 2.**
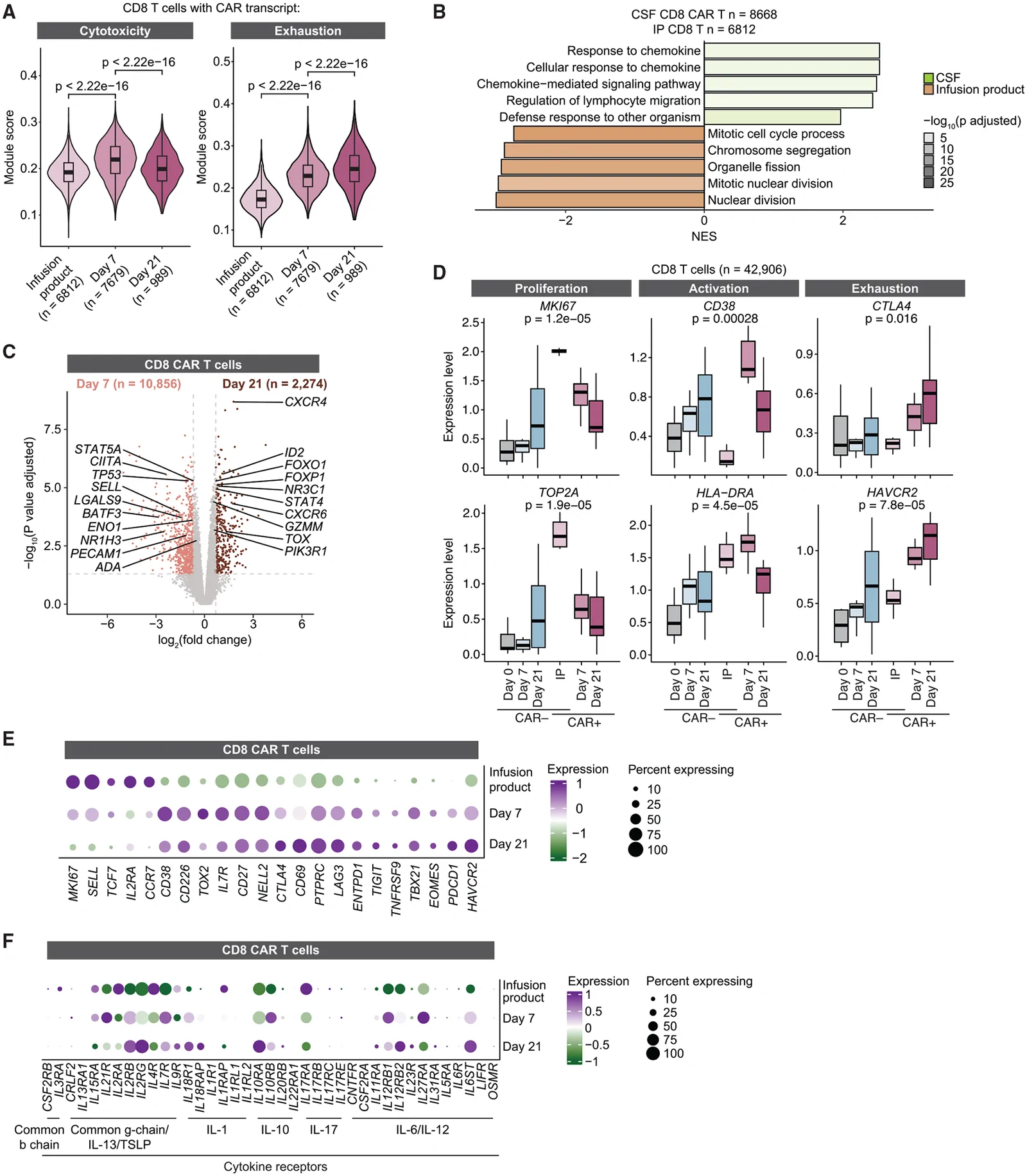
Longitudinal evolution of CAR and non-CAR T cells in the CSF post infusion (A) Cytotoxicity and exhaustion scores of CD8 T cells with CAR transcript in infusion product (IP; *n* = 6,812 cells) vs. CD8 T cells with CAR transcript in the CSF at day 7 (*n* = 7,679) and day 21 (*n* = 989). (B) GSEA comparing CSF CD8 CAR T cells (days 7 and 21) to IP. (C) Differential gene expression between days 7 (*n* = 10,856) and 21 (*n* = 2,274) CD8 T cells in the CAR+ cluster. Highlighted genes: Benjamini-Hochberg-adjusted *p* < 0.05 and absolute fold change > 2. (D) Expression levels of selected genes in CAR+ and CAR− clusters (*n* = 8 patients). (E and F) Average expression (color) and percent of cells expressing (dot size) for (E) key genes and (F) cytokine receptors across time points. All panels show data from the discovery cohort. Data in (A) and (D) depict median (center), inter-quartile range (IQR) (box), and 1.5 × IQR (whiskers). *p* values: Wilcoxon rank-sum test (A), Benjamini-Hochberg-adjusted empirical *p* values (B), quasi-likelihood F test (C), or Kruskal-Wallis test (D). See also Figures S2 and S3.

To assess whether the elevated cytotoxicity of CD8 CAR T cells in the CSF (Figure 1H) was driven by true *in vivo* exposure rather than manufacturing conditions, we tracked their phenotypic evolution from the IP through days 7 and 21 post infusion. Supporting robust *in vivo* activation, cytotoxicity peaked at day 7, significantly exceeding IP levels (*p* < 2.22E−16), before declining by day 21, though remaining modestly elevated above baseline (Figure 2A, left). Concurrently, exhaustion markers increased progressively from the IP to day 21, a temporal pattern consistent with ongoing antigen engagement and activation *in vivo* (Figure 2A, right). Pathway and gene enrichment analyses also reflect this functional shift, with downregulated proliferation programs and upregulated lymphocyte activation and migration in CD8 T cells post infusion (Figures 2B and S3A). These data indicate a transition from a proliferative to an effector state upon CSF entry, with transcriptional remodeling indicative of functional engagement and emerging exhaustion.

At day 21, CD8 CAR T cell numbers decreased (Figure S3B) alongside transcriptional shifts toward chronic stimulation (Figure 2C). Upregulation of the trafficking receptors *CXCR4* and *CXCR6* suggested altered tissue trafficking.^38^ Concurrently, diminished proliferation, with downregulated *MKI67* and *TOP2A* and fewer cells in S and G2/M phases (Figures 2D and S3C), coincided with increased exhaustion markers such as *CTLA4* and *HAVCR2* (Figures 2D and S3D). Longitudinal tracking of subset-defining genes confirmed a progressive CD8 CAR T cell differentiation: from infused proliferating memory T cells (*MKI67*, *SELL*, and *TCF7*) to day 7 activation (*CD38*) and a day 21 stimulated/exhausted phenotype (*CTLA4*, *LAG3*, and *HAVCR2*) (Figures 2E and S3E). Conversely, non-CAR CD8 T cells exhibited delayed activation, with *MKI67*, *TOP2A*, and *CD38* peaking at day 21, consistent with bystander activation (Figures 2D and S3F).

Although low CSF cell numbers limited functional assays, gene expression analysis offered insight into functional potential. At day 7, CD8 CAR T cells upregulated effector (*IL12RB1* and *IL21R*) and memory (*IL7R*)-associated cytokine receptors compared with the IP (Figure 2F). By day 21, these signals waned alongside upregulation of immunosuppressive markers, including *TGFB1* and the inhibitory receptor *IL10RA* (Figures 2F and S3G). However, sustained expression of activating receptors *IL6ST* and *IL2RG* suggests retained functional potential despite this emerging immunosuppression.

Together, these longitudinal dynamics highlight distinct temporal kinetics: CD8 CAR T cells undergo activation peaking at day 7 followed by progressive exhaustion, whereas non-CAR T cells exhibit a delayed wave of bystander activation at day 21.

### Longitudinal evolution of GBM tumor tissue post infusion

**Figure 3.**
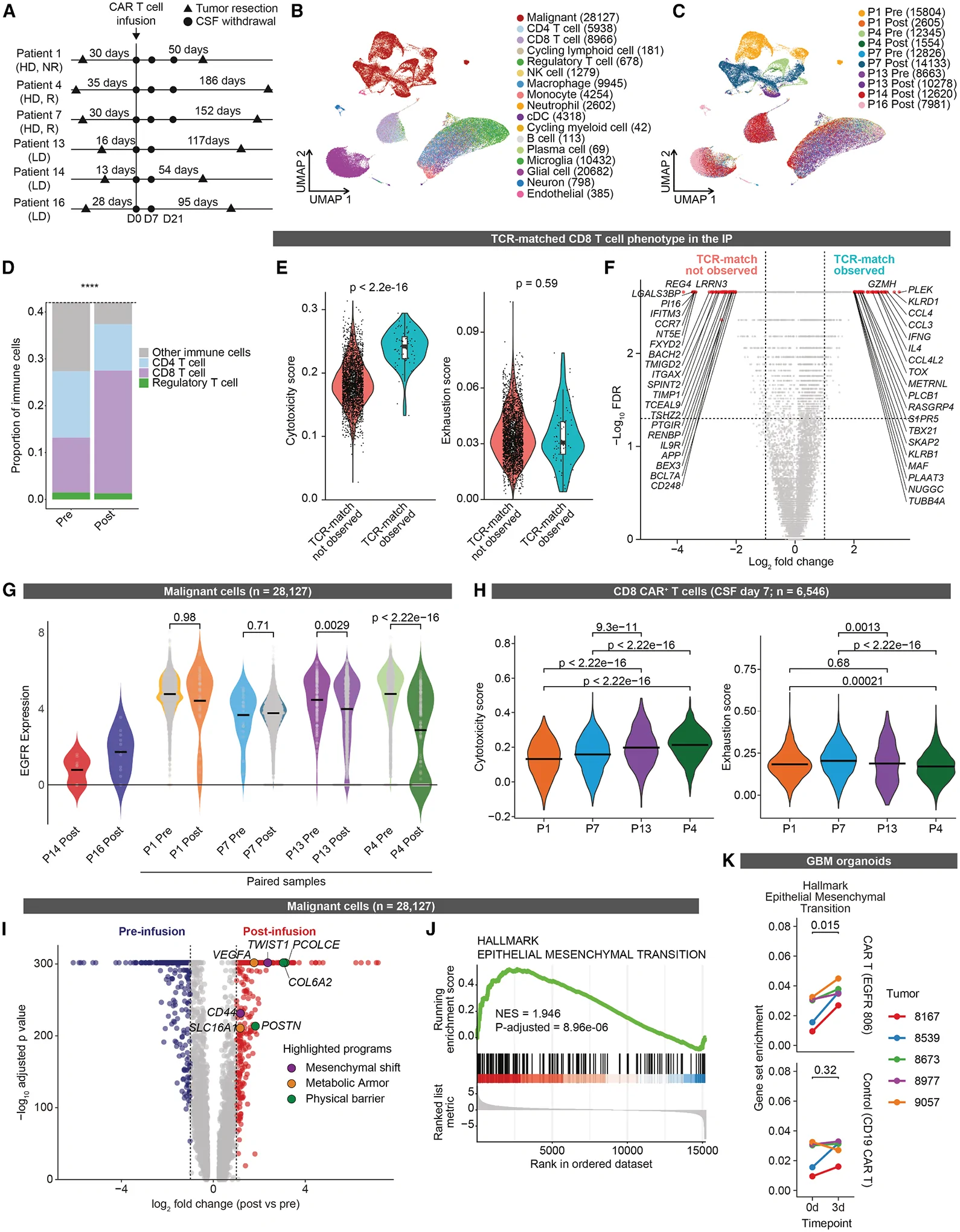
Longitudinal evolution of GBM tumor tissue post infusion (A) Sample collection timeline. HD: high-dose; LD: low-dose. (B and C) UMAP of snRNA-seq profiles (*n* = 98,809) colored by (B) cell type and (C) sample. (D) Tumor tissue T cell composition (*n* = 48,817 cells and 15,582 T cells). (E and F) CD8 T cells from the IP with TCR-matched clones in the post-infusion tumor tissue vs. those without matched clones: (E) cytotoxicity/exhaustion scores and (F) differentially expressed genes (*n* = 67 TCR-matched and 2,523 non-matched cells). (G) EGFR expression in malignant cells (*n* = 28,127). (H) Cytotoxicity/exhaustion scores in CSF CD8 CAR T cells at day 7 (*n* = 6,546). (I) Differentially expressed genes in pre- vs. post-infusion malignant cells (*n* = 28,127). (J and K) Enrichment of HALLMARK_EPITHELIAL_MESENCHYMAL_TRANSITION in (J) malignant cells (*n* = 28,127 cells, GSEA) and (K) GBOs co-cultured with EGFR-806-epitope vs. CD19 CAR T cells (*n* = 5 tumors, 2 time points per tumor); organoids derived from the same tumor are paired. Data in (E) represent median (center), IQR (box), and 1.5× IQR (whiskers). In (G) and (H) lines represent mean. *p* values: one-sided Fisher exact test (**** = CD8 T cell composition *p* < 0.0001) (D), Wilcoxon rank-sum test (E, G, and H), Multiple Monte Carlo (MMC) test with resampling to account for class imbalance (F), logistic regression, Bonferroni-adjusted (I), Benjamini-Hochberg-adjusted (J), and AddModuleScore paired *t* test (K). See also Figure S4.

Having defined the activation dynamics of CAR and non-CAR T cells in the CSF, we next assessed corresponding changes in tumor tissue following CAR T cell infusion. We analyzed matched pre- and post-treatment GBM samples from the six patients who underwent a second resection (50–186 days post infusion; Figure 3A). Histological analysis showed a spectrum of post-infusion changes ranging from prominent treatment effects (P1) to recurrent tumor with a high proliferative index (P7). Immunohistochemical (IHC) staining revealed a mild increase in immune infiltration in the post-infusion tissue. Lymphocytes were often located close to vessels, but many were scattered within the tumor or treated areas (Table S4; Figure S4A; Data S1).

Single-nucleus RNA-seq (snRNA-seq; 10 of 12 samples with sufficient cells for analysis, *n* = 98,809) resolved distinct immune and tumor compartments (Figures 3B and 3C; Data S1), with cell types and neoplastic (hereafter “malignant”) cells identified via canonical lineage markers (Figure S4B) and inferred copy-number alterations (Figure S4C). Sample composition, particularly the abundance of malignant cells, varied by individual rather than time point (Figure S4D), likely reflecting intrinsic tumor heterogeneity rather than a response to therapy. Non-CAR CD8 T cells in post-infusion tumors showed an overall increase compared with pre-treatment samples (Figure 3D, *p* < 0.0001), with a significant increase in two of the three high-dose pairs (Figure S4E). These cells also displayed a subtle but significant shift toward increased expression of genes associated with activation (*CD38* and *HLA-DRA*) and proliferation (*MKI67*) (Data S1).

At the time of the second resection, we were unable to detect CAR T cells in the tissue by qPCR or alignment to snRNA-seq data. However, TCR-based tracking identified a few small (2–17 cells across 1–8 clones per pair) TCR-matched clones traversing from the IP to post-infusion tissue in 5 of 6 tumors. Although this time point likely missed the window of peak tumor infiltration, retrospective analysis of the IP revealed that these matched clones possessed a distinct phenotype prior to infusion. In the two cases with sufficient cell numbers (≥10 cells, P7 and P14), the matched clones were significantly enriched for cytotoxic, trafficking, and effector markers, such as *IFNG*, *GZMH*, and *KLRD1* (Figures 3E and 3F; Data S1).

To evaluate immunologic selection pressure on persisting malignant cells, we assessed CAR target expression. Although IL13Rα2 is difficult to quantify due to known detection limitations,^39^ IHC confirmed its continued presence post infusion (Figure S4F; Data S1).^40^ In contrast, *EGFR* was quantifiable by snRNA-seq and significantly downregulated in post-treatment malignant cells in 2 of 4 paired samples (P4, responder; P13, prolonged survival), validated via bulk RNA-seq and RNAscope (Figure 3G; Data S1). Notably, day 7 CSF CAR T cells from these patients exhibited elevated cytotoxicity^33^ and minimal exhaustion^34^ (Figure 3H), suggesting that high early immune pressure drove some antigen loss through immunoediting.

Beyond target downregulation, persisting malignant cells showed no consistent change in overall Neftel et al.^41^ cell-state composition (Figure S4G). However, global differential gene expression comparing post- to pre-infusion malignant cells revealed upregulation of key drivers of transcriptional reprogramming, extracellular matrix (ECM) remodeling, and metabolic competition (Figure 3I), consistent with previously described coordinated mechanisms of immune evasion in GBM.^42–46^ Specifically, post-infusion malignant cells upregulated *CD44* and *TWIST1* and were enriched for the Hallmark epithelial-to-mesenchymal transition (EMT) gene set (Figures 3J and S4H), reflecting a mesenchymal-like adaptive injury response to withstand immune pressure.^47–50^ This shift was supported by significantly enriched transcription factor activity of *TWIST1* (*p* = 5.1 × 10 ^−6^, Wilcoxon rank-sum test) and nuclear factor κB (NF-κB) (*p* = 7.5 × 10^−5^, Wilcoxon rank-sum test)—known to orchestrate mesenchymal plasticity and immune evasion in GBM.^43,46^ The EMT signature was elevated across all Neftel et al.^41^ cell states, reaching statistical significance in the mesenchymal (MES)-like and astrocyte (AC)-like states (Figure S4I), independent of response status or dose level (Figure S4J). Furthermore, analysis of data from patient-derived GBM organoids (GBOs) co-cultured with EGFR-806-targeted CAR T cells (targeting one of the two antigens in the clinical trial CAR product)^51^ showed that the GBO model recapitulated the rapid EMT enrichment observed in the patient cohort (Figure 3K, *p* = 0.015). These findings nominate mesenchymal shift as an escape mechanism for immunotherapy and potential therapeutic vulnerability, echoing previous studies showing that blocking upstream drivers of MES induction mitigates radioresistance in preclinical GBM models.^43^

Collectively, these data suggest a reciprocal landscape wherein therapeutic immune pressure in the tumor tissue is associated with EGFR antigen editing, while persisting malignant cells engage transcriptional programs to withstand immune elimination.

### Endogenous immune cells in CSF evolve after CAR T cell infusion

**Figure 4.**
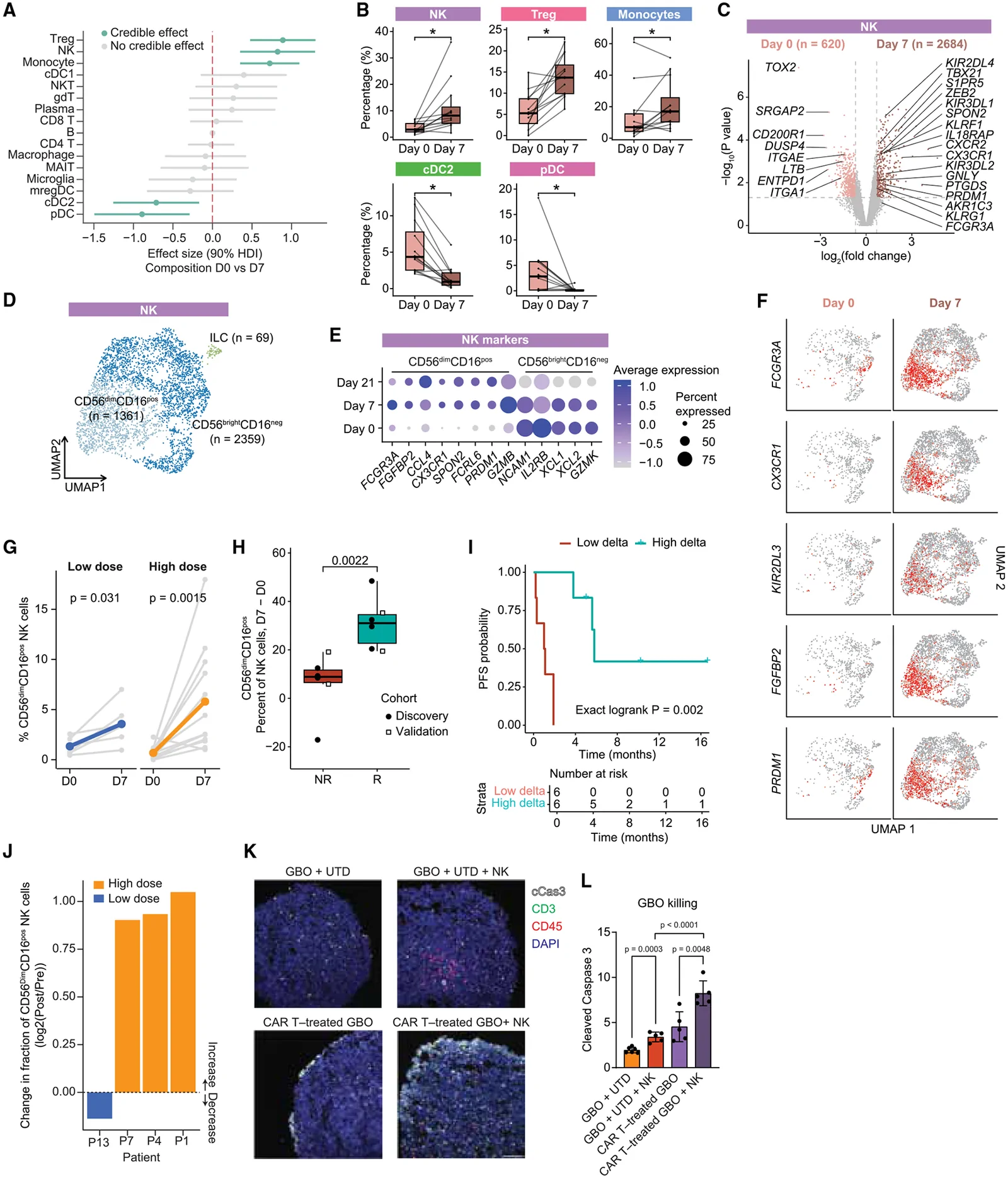
Phenotypic shift of NK cells is associated with improved clinical outcomes (A) Bayesian composition analysis of CSF non-CAR immune subsets (day 0 vs. day 7; *n* = 12; high-dose). Green: significant effect (90% high-density interval [HDI]). (B) Proportional abundance of immune subsets, day 0 vs. day 7 (*n* = 12 patients). Paired points represent patients; asterisks: >90% inclusion probability (10% false discovery rate [FDR]). (C) Differential gene expression in day 0 vs. day 7 CSF NK cells (*n* = 3,304 cells; discovery cohort). (D) UMAP of CSF NK cells (all time points; *n* = 3,789 cells; discovery cohort) annotated by subtype. (E) Expression of subtype-defining markers. Dot size: percent expressing; color: average expression (*n* = 3,789 cells; discovery cohort). (F) UMAP from (D) colored by marker expression at day 0 and day 7 (*n* = 3,304 cells; discovery cohort). (G) Proportion of CD56^dim^ CD16^pos^ NK cells among non-CAR T and NK cells in the CSF (day 0 and day 7) by dose (*n* = 6 low-dose patients; *n* = 12 high-dose patients). Paired points represent individual patients; colored lines represent mean. (H) Change in proportion of CD56^dim^CD16^pos^ NK among CSF NK cells (day 0 to day 7), non-responders (NRs, *n* = 6) vs. responders (Rs, *n* = 6). (I) Kaplan-Meier curves of progression-free survival (PFS) stratified by median change in proportion of CD56^dim^CD16^pos^ NK cells among CSF NK cells day 0 to day 7 (*n* = 12 patients, high dose). (J) Log-ratio change in CD56^dim^CD16^pos^ fraction among all NK cells in paired tumor tissue (*n* = 4 patients). (K) Representative images of GBO co-cultured with untransduced donor T cells (UTD), bivalent CAR T cells, and/or NK cells (scale bar: 100 μm). (L) Tumor cell death (cleaved caspase-3) in GBO co-cultures, normalized to total nuclei (*n* = 6 replicates). Data in (B) and (H) represent median (center), IQR (box), and 1.5× IQR (whiskers). Data in (L) represent mean ± SD. *p* values: two-sided paired Wilcoxon signed-rank test (G), two-sided Wilcoxon rank-sum test (H), exact log-rank test (I), or unpaired *t* test (L). See also Figure S5.

Alongside the increase in non-CAR T cell numbers (Figure S1J), endogenous immune populations undergo compositional changes after infusion (Figure S1F). To quantify these shifts, we applied a Bayesian model that accounted for sample variability and compositional constraints to identify cell types with credible changes in relative abundance. This analysis revealed significant proportional increases in NK cells, Treg cells, and monocytes, as well as decreases in plasmacytoid dendritic cells (DCs) (pDCs) and type 2 conventional DCs (cDC2s) (Figure 4A), dynamics that were independent of corticosteroid exposure (Table S1; Data S1). These populations play distinct and often opposing roles: NK cells are cytotoxic and have been associated with enhanced CAR T cell efficacy in hematologic malignancies,^52^ whereas Tregs and myeloid cells are typically immunosuppressive and have been linked to resistance mechanisms in CAR T therapy in solid tumors^53,54^ and in GBM under standard treatment.^55,56^ Although changes in immune cell composition were generally consistent, we observed some variation across patients (Figure 4B) and across days (Figure S5A), prompting us to investigate whether the expansion of specific populations correlated with clinical outcomes.

### Phenotypic shift of NK cells is associated with improved clinical outcomes

Beyond numeric expansion, CSF NK cells underwent a distinct phenotypic shift post infusion, whether by local adaptation or recruitment. Differential gene expression analysis (day 7 vs. day 0, discovery cohort) revealed significant upregulation at day 7 of genes associated with effector and cytokine-secreting functions, including *KIR3DL1*, *CX3CR1*, *KLRG1*, and *IL18RAP*, whereas genes such as *ENTPD1*, *DUSP4*, and *TOX2* were enriched at baseline (Figure 4C). Categorically, these changes resolved into two NK cell subsets and a cluster of innate lymphoid cells (ILCs) (Figures 4D and S5B). Longitudinal tracking revealed a notable day 7 expansion of the CD56^dim^CD16^pos^ cytotoxic NK subset (Figures 4E and S5C), a dynamic confirmed in the validation cohort (Figure S5D). As expected, this subset expressed cytotoxic markers such as FCGR3A, CX3CR1, KIR2DL3, FGFBP2, and PRDM1 (Figure 4F), characteristic of the predominant mature, highly cytotoxic human NK population capable of mediating direct target cell lysis and antibody-dependent cellular cytotoxicity.^57–59^ The increasing abundance of this population suggests an early functional shift within the CSF NK cell compartment after CAR T therapy. Notably, this phenotypic shift exhibited dose dependency: in the low-dose cohort, the CD56^dim^CD16^pos^ proportion increased on average from 1.3% to 3.6%, while the high-dose cohort showed a superior expansion from 0.7% to 5.8% (Figure 4G).

The clinical relevance of this expanding CD56^dim^CD16^pos^ NK population was underscored by the association between the magnitude of this phenotypic shift and improved clinical outcome. Across both cohorts, responders exhibited a significantly greater increase in the proportion of CD56^dim^CD16^pos^ NK cells between days 0 and 7 compared with non-responders (median +31.0% vs. +8.9%, *p* = 0.0022; Figure 4H). Stratifying the cohort by the median increase (19.3 percentage points) revealed a strong association between higher NK cell expansion and improved PFS (median PFS 5.8 vs. 1.05 months, *p* = 0.002; Figure 4I) and OS (median OS 19.3 vs. 7.4 months, *p* = 0.03; Figure S5E). These findings suggest that early expansion of cytotoxic NK cells in the CSF is a predictive biomarker of clinical benefit.

These phenotypic changes were durable, persisting beyond day 7 with continued expression observed at day 21 (Figures S5F and S5G). They were also still evident in all of the high-dose-treated post-infusion tumor tissues (Figure 4J; Data S1), mirroring the phenotypic shift observed in the CSF and supporting a therapy-associated effect across compartments.

Given the association with clinical response, we evaluated the potential for increased tumor killing *ex vivo*. We generated patient-derived GBOs that recapitulate key immune interactions observed *in vivo* and co-cultured them with bivalent CAR T cells alone, NK cells alone, or a combination, validated across two independent donor batches with 6 replicates each. The combination of CAR T cells and NK cells induced significantly higher tumor cell lysis, as determined by cleaved caspase-3 staining after 6 days of co-culture, compared with either population alone (Figures 4K, 4L, and S5H). To determine whether this enhanced cytotoxicity was driven by a heightened state of immune activation, we profiled the release of inflammatory mediators. This analysis revealed that the combination triggered a robust upregulation of effector cytokines, including IFN-γ, tumor necrosis factor α (TNF-α), and granulocyte-macrophage colony-stimulating factor (GM-CSF). In contrast, secretion of the T cell-restricted cytokine interleukin (IL)-2^60^ remained unchanged (Figure S5I).

Together, these findings suggest a synergy whereby endogenous NK cells augment CAR T cell-mediated tumor lysis, with NK cell expansion associated with improved clinical outcomes.

### High baseline myeloid immunosuppression is associated with poor response

**Figure 5.**
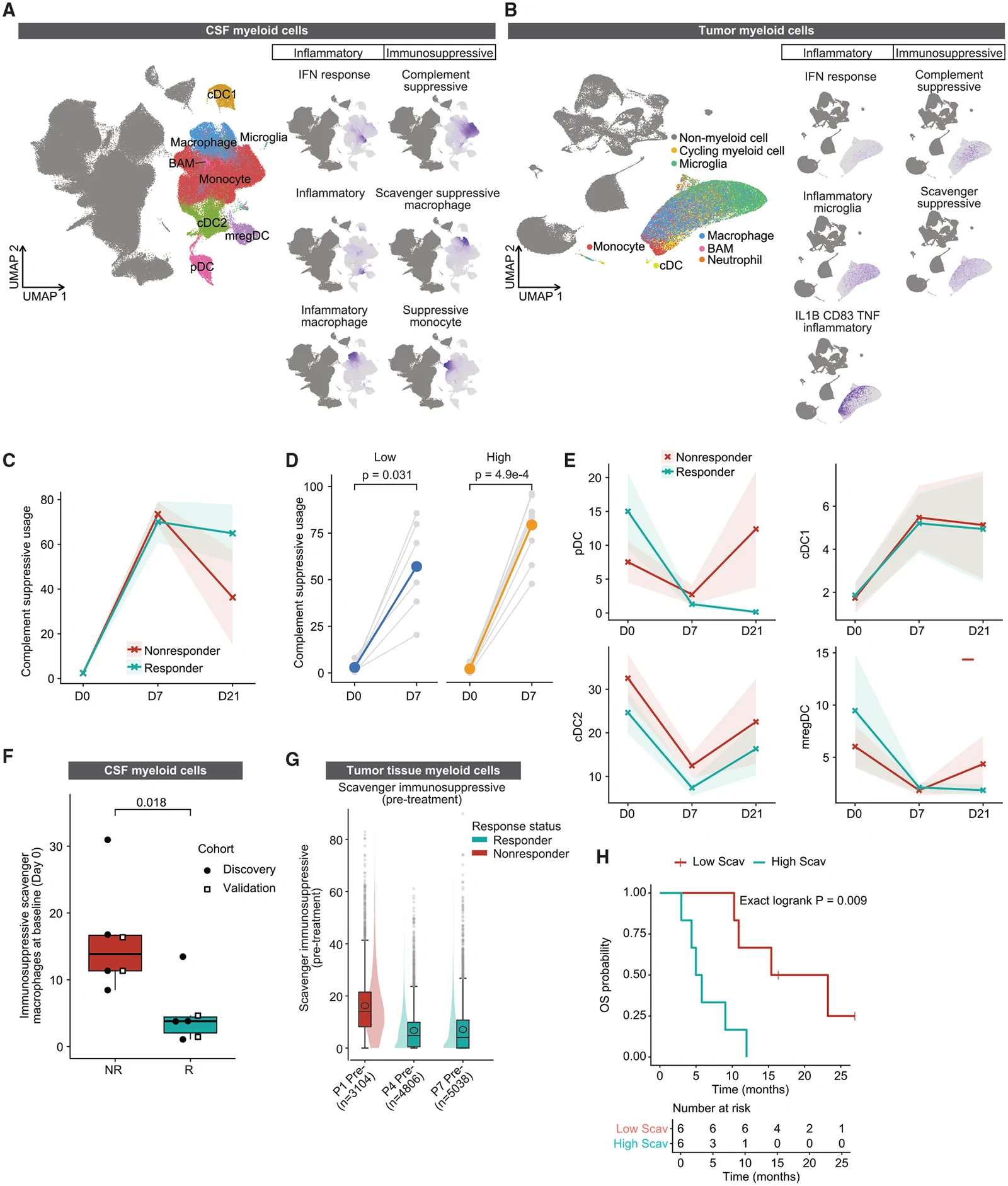
High baseline myeloid immunosuppression is associated with poor response (A and B) UMAP of myeloid cells and activity programs in (A) CSF (full cohort, *n* = 74,761 cells) and (B) tumor tissue (*n* = 31,593 cells). (C) Longitudinal dynamics of immunosuppressive complement program usage in the high-dose CSF cohort (*n* = 32 samples in 12 patients). (D) Immunosuppressive complement program usage (CSF day 0 and day 7) by dose (*n* = 6 low-dose patients; *n* = 12 high-dose patients). Paired points represent individual patients; colored lines represent mean. (E) Longitudinal dynamics of the four DC program usages in CSF (*n* = 32 samples, 12 high-dose patients). (F and G) Baseline scavenger program usage in (F) CSF from non-responders (NRs; *n* = 6, high dose) vs. responders (Rs; *n* = 6, high dose), and (G) tumor tissue (*n* = 3, all available high-dose patients). (H) Kaplan-Meier curve of overall survival (OS) stratified by median baseline scavenger program usage in CSF (*n* = 12 patients, high-dose). Lines in (C) and (E) represent mean usage stratified by response; shading indicates 95% confidence intervals (CIs). Data in (F) and (G) represent median (center), IQR (box), and 1.5× IQR (whiskers). *p* values: two-sided paired Wilcoxon signed-rank test (D), two-sided Wilcoxon rank-sum test (F), or exact log-rank test (H). See also Figure S6.

Although the expansion and activation of NK cells appeared to support CAR T cell efficacy, other immune populations that rose in abundance following infusion (Figure 4A), namely myeloid cells and Tregs, may contribute to immunosuppressive feedback mechanisms that limit therapeutic impact. We defined myeloid cell types and their functional states by mapping myeloid cells from the tumor tissue to the reference programs of Miller et al.^56^ and applied consensus non-negative matrix factorization (cNMF) to discover unique programs within the CSF (Figure S6A). This approach allowed us to define myeloid cell types and activities in both the CSF (Figures 5A, S6B, and S6C) and tumor tissue (Figures 5B and S6D).

Longitudinal analysis revealed a shift in myeloid subsets in the CSF in response to CAR T cell infusion. Specifically, we observed a significant increase in myeloid cells expressing the Complement Immunosuppressive program defined in gliomaassociated myeloid cells^56^ (Figure 5C). This transition was dose dependent (Figure 5D) and mirrored the shift from immune-activating to suppressive myeloid states reported in other CNS CAR T cell trials.^19^ In parallel, several antigen-presenting subsets—pDCs, cDC2s, and mature regulatory DCs—diminished over time, whereas cDC1s, which are critical for cross-presentation to CD8 T cells, increased (Figure 5E). These shifts were consistent across patients but did not correlate with patient response.

In contrast, baseline composition of myeloid states was strongly associated with clinical outcome. High expression of the scavenger-like program in the CSF prior to CAR T cell infusion was significantly associated with lack of response (Figure 5F), with a similar trend observed in the tumor tissue (Figure 5G). Furthermore, patients with high scavenger program usage in the CSF exhibited significantly shorter OS (Figure 5H, *p* = 0.009) and a trend toward shorter PFS (Figure S6E, *p* = 0.1). Biologically, this program is defined by the expression of scavenger receptors such as *MSR1* (CD204), *MRC1* (CD206), and *CD163*, all of which have known roles in immunosuppression.^56^ This finding aligns with recent work by Miller et al., which identified the scavenger program as associated with resistance to PD-1 checkpoint blockade and was a predictor of poor survival in GBM.^56^

Together, these findings highlight the scavenger immunosuppressive program as a key driver of resistance and a specific actionable target to sensitize the GBM microenvironment to CAR T cell therapy.

### Treg expansion inversely correlates with tumor size reduction

**Figure 6.**
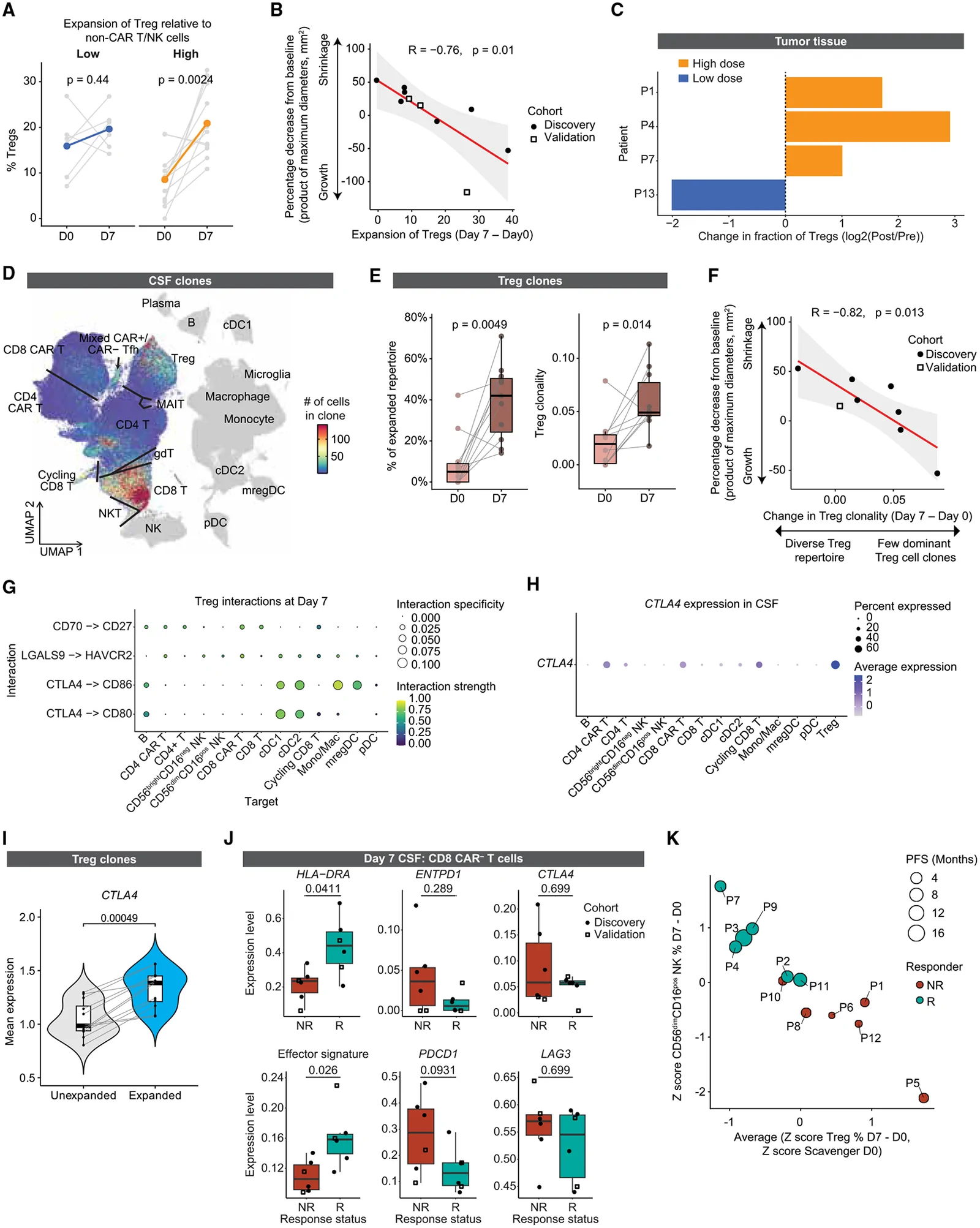
Treg expansion inversely correlates with tumor size reduction (A) Proportion of non-CAR Tregs among non-CAR T and NK cells in the CSF (days 0 and 7) by dose (*n* = 6 low-dose patients; *n* = 12 high-dose patients). Paired points represent individual patients; colored lines represent mean. (B) Correlation between Treg expansion (day 0 to day 7) and tumor size reduction for patients with measurable disease at baseline (*n* = 10 patients, high dose). (C) Log-ratio of Treg expansion among all immune cells in tumor tissue (*n* = 4 pairs). (D) UMAP of CSF cells colored by clone size. (E) Left: proportion of expanded non-CAR Treg clones among expanded non-CAR T cell clones. Right: change in clonality of the non-CAR Treg TCR repertoire from day 0 to day 7 among patients with non-CAR Treg TCRs detected at both days (*n* = 10, high dose). Paired points represent individual patients. (F) Correlation between the change in non-CAR Treg clonality from day 0 to day 7 and tumor size reduction, in patients with measurable disease at baseline and non-CAR Treg TCRs detected at both days (*n* = 8 patients, high dose). (G) Selected ligand → receptor interactions (non-CAR Treg ligands) at day 7 (*n* = 12, high dose). (H and I) *CTLA4* expression across CSF (H) immune cells and (I) expanded vs. non-expanded non-CAR Treg clones, with paired points representing individual patients. (J) Average expression of selected markers in CSF non-CAR CD8 T cells at day 7, comparing Rs and NRs (*n* = 12 patients, high dose). (K) *Z* score change in CD56^dim^CD16^pos^ NK cells (day 0 to day 7) vs. a composite immunosuppression score (mean *Z* score of day 0 to day 7 change in non-CAR Tregs and baseline scavenger macrophages) (*n* = 12 patients, high dose). In (B) and (F), shading indicates 95% CI. In (E), (I), and (J), data represent median (center), IQR (box), and 1.5× IQR (whiskers). In (G), dot size represents interaction specificity and color interaction strength. *p* values: Pearson’s R (B and F), paired Wilcoxon signed-rank test (A, E, and I), or two-sided Wilcoxon rank-sum test (J). See also Figure S7.

In parallel with the changes observed in NK and myeloid states, Tregs also expanded significantly in the CSF (Figure 4A), increasing from day 0 to day 7 in the majority of patients (Figure 4B) in a dose-dependent manner (Figure 6A). CSF proteomics corroborated these trends, revealing elevated NK-associated (IL-2, IL-15, and IL-12p40)^61^ and Treg-associated (IL-4 and IL-10)^62^ cytokines (Figure S7A). Notably, the magnitude of Treg expansion was inversely correlated with tumor size reduction (Figure 6B; Pearson’s R = −0.76, *p* = 0.01), suggesting that greater Treg expansion limits therapeutic response. This increase in Tregs was also evident in all high-dose tumor tissue samples (Figure 6C).

Further TCR sequencing revealed increased clonal expansion within the Treg compartment. Expanded clones (>1 cell) were increasingly composed of CD8 T cells and Tregs over time (Figures 6D and 6E, left, *p* ≤ 0.0049; Figure S7B). Treg clonality—a metric reflecting dominance by a few expanded clones—significantly increased from day 0 to day 7 (Figure 6E, right, *p* ≤ 0.014; Figure S7C). Notably, high Treg clonality at day 7, reflecting dominance by a few highly expanded clones, was strongly associated with poor tumor size reduction (Figure 6F; Pearson’s R = −0.82, *p* = 0.013). Expanded Treg clones also transitioned over time from a quiescent, stem-like state (*TCF7* and *LEF1*) to a highly proliferative (*MKI67* and *CDK1*), trafficking (*CCR8*), and potently suppressive (*CTLA4*, *LGALS1*, and *TNFRSF18*) phenotype (Figure S7D). Together, these findings suggested that early, antigen-driven Treg expansion and activation impair tumor control.

Tregs exert immunosuppressive effects through multiple mechanisms, including ligand-receptor interactions. Ligand-receptor interaction analysis (Figure 6G; Table S5; Data S1) identified a potential interaction between Treg-derived *LGALS9* (galectin-9) and *HAVCR2* (TIM-3) on myeloid cells at day 7, suggesting that Tregs may promote immunosuppressive myeloid phenotypes early during CAR T cell therapy. The CTLA-4 interactions (Figure 6G) point to a second inhibitory pathway in which high expression of CTLA-4 in Tregs (Figure 6H), particularly expanded Tregs (Figure 6I), may dampen DC activity via the CD80/CD86 axis.

Such interactions could reduce DC priming capacity, thereby limiting the activation of non-CAR CD8 T cell responses to bystander antigens. Because T cell priming can also occur within peripheral tertiary lymphoid structures (TLSs),^63^ we assessed TLS-related features in the CSF. In line with this hypothesis, four characteristic TLS signatures^63^ were significantly elevated at day 7 compared with day 0 (Figure S7E), suggesting that TLS-like priming could potentially occur within the GBM tumor tissue^64,65^ or CSF-exposed leptomeningeal niches.^66^ Further supporting a bystander impact of CAR T therapy on non-CAR T cells, non-responders exhibited reduced non-CAR CD8 T cell activation at day 7, marked by lower *HLA-DRA* expression and a diminished effector signature (Figure 6J). One interpretation is that insufficient priming, potentially mediated by suppressive Treg-DC interactions^67,68^ or by defective composition of TLS-like structures,^63^ may contribute to suboptimal non-CAR T cell responses during ICV CAR T therapy. Together, these findings highlight immunoregulatory pathways, particularly those involving *CTLA4*, as actionable targets to limit Treg suppression and reinvigorate the endogenous immune response.

Collectively, these data define three endogenous immune determinants of clinical outcome: the expansion of cytotoxic NK cells correlates with response, whereas Treg expansion and a high baseline abundance of suppressive scavenger myeloid cells correlate with disease progression (Figures 6K, S7F, and S7G).

## DISCUSSION

Profiling the longitudinal CSF and tumor landscape in recurrent GBM (days 0–21) revealed distinct immunological processes underlying clinical outcomes. We confirmed that CAR T cells activate locally, likely engaging target antigens, as evidenced by acquiring a neoantigen-specific TIL signature. This activation peaks at day 7 and is dominated by cytotoxic CD8 CAR T cells. While the expression of exhaustion-related genes emerges by day 21, cytotoxicity-associated genes, pro-inflammatory cytokines, and cytokine receptors remain expressed, suggesting sustained functional potential. In patients with available post-CAR tumor tissue, CAR T cells were not detected, consistent with prior GBM studies in which robust detection was largely limited to short (≤14 day) resection intervals.^31,32^ Nonetheless, we observed signs of T cell infiltration, immune pressure on *EGFR* expression, and malignant cell remodeling in these post-infusion tumor tissues.

Beyond the CAR T cells, host endogenous immune remodeling emerged as a critical determinant of efficacy. On the effector side, non-CAR CD8 T cells showed progressive activation over time, indicating broad engagement of endogenous anti-tumor immunity. Additionally, favorable outcomes were strongly associated with early, dose-dependent expansion of cytotoxic NK cells. *Ex vivo* modeling confirmed increased killing when combining CAR T and NK cells, suggesting positive synergy. Future strategies could exploit this synergy by combining CAR T cells with NK cell engagers^69^ or cytokine signals to amplify this early effector wave. In contrast, limited efficacy was characterized by the accumulation of Tregs and baseline levels of “scavenger” myeloid cells. The scavenger myeloid signature is detectable in the CSF and could serve as a non-invasive liquid biopsy to identify patients with immunosuppressive microenvironments who might benefit from neoadjuvant myeloid-reprogramming agents. Specifically, pharmacological inhibition of p300 (e.g., with GNE-781) has been shown to reprogram scavenger-positive myeloid cells toward an inflammatory phenotype,^56^ offering a potential combinatorial strategy to prevent myeloid-mediated suppression. Concurrently, Treg accumulation may dampen anti-tumor immunity via CTLA-4-mediated inhibition of priming or galectin-9-TIM-3 interactions with myeloid cells. As such, limiting Tregs, not only in the IP^70^ but also in the host following product infusion, may further support efficacy. This may be achieved through Treg-depleting antibodies (e.g., anti-CD25^NIB^)^71^ or targeted therapies disrupting key interactions identified in our study (e.g., anti-CTLA-4).^72^ Alternatively, our data support engineering IL-10RA-deficient CAR T cells to interrupt the deleterious effect of Treg-secreted IL-10. Beyond these examples, our dataset provides multiple resources for identifying additional therapeutic pathways to inform the next generation of CAR T cell strategies for brain tumors.

In summary, our results suggest that the CSF is a dynamic compartment with immunological relevance, carrying important implications for monitoring and treating patients with GBM undergoing immunotherapy. Our findings reveal that the host endogenous immune compartment plays a critical role in ICV CAR T cell therapy for GBM, an insight that should inform future treatment strategies combining CAR T cells with approaches that harness or modulate the patient’s immune response to enhance efficacy.

### Limitations of the study

First, our study is limited by the small sample size (*n* = 18) inherent to phase 1 trials and variable single-cell capture efficiency, which restricts statistical power compared with larger studies. This also precluded the assessment of sex differences in response and immune dynamics. Second, paired tumor tissue analysis (*n* = 6) involved highly variable intervals between infusion and resection (50–186 days), likely capturing late-stage remodeling rather than peak intratumoral immune activity, with intrinsic tumor heterogeneity confounding comparisons. Third, the temporal disconnect between robust CSF activation and the scarcity of detectable CAR T cells in post-treatment tumors limits our ability to definitively confirm antigen engagement *in situ*. Fourth, although TCR tracking identified shared clones between the IP and other compartments, we cannot exclude the possibility that some clonotypes represent pre-existing endogenous T cells. Fifth, target expression quantification was complicated by spatial heterogeneity and a lack of specific reagents, particularly for IL13Rɑ2.^39^ Finally, inferred ligand-receptor interactions rely on transcriptomic proxies and warrant validation via spatial proteomics to confirm physical engagement *in situ*.

## RESOURCE AVAILABILITY

### Lead contact

Requests for further information and resources should be directed to and will be fulfilled by the lead contact, Dana Silverbush (dana.silverbush@pennmedicine.upenn.edu).

### Materials availability

This study did not generate new, unique reagents.

### Data and code availability

Processed single-cell data and annotations have been deposited in GEO under accession number GEO: GSE296419 and are available through the Broad Single Cell Portal (SCP3237). Raw sequencing data have been deposited under regulated access in the NIH dbGaP database. Analysis code is available at https://github.com/SilverbushLab/Bivalent-CAR-T-cell-therapy-in-recurrent-GBM.

Any additional information required to reanalyze the data reported in this paper is available from the lead contact upon request.

## STAR★METHODS

### EXPERIMENTAL MODEL AND STUDY PARTICIPANT DETAILS

#### Human subject selection

This exploratory analysis uses samples obtained from a previously published Phase I clinical trial of bivalent EGFR-IL13Rα2 CAR T cells in recurrent GBM.^14^ All CSF and tumor samples analyzed here were collected under the clinical trial NCT05168423 (University of Pennsylvania IRB Protocol #850297), which focused on safety, feasibility, and clinical response as primary and secondary endpoints. This study extends beyond those endpoints to investigate immune correlates of response using banked specimens. Inclusion in the discovery cohort was based on a timing cutoff (CAR T infusion by May 2024, with D21 samples banked by June 2024). All patients treated by this date and all associated time points (infusion product, D0 CSF, D7 CSF, D18–21 CSF, and paired tumor resections, if applicable) were included; no samples were excluded. The remaining high-dose patients were assigned to the validation cohort, and all low-dose patients were analyzed as a separate cohort. For these patients (validation and low-dose), D0 and D7 CSF samples were profiled, along with all available tumor tissue. In addition, for patients in the validation and low-dose cohorts with available post-infusion tumor tissue (P13, P14, and P16), the corresponding infusion products were profiled and used for TCR tracing (Data S1). Patient numbers reflect the chronological order of infusion. Additional patient data and clinical details, including the clinical trial protocol, are provided in Table S1 and the clinical trial publication.^14^ The influence of sex and gender on the results is discussed in limitations of the study.

#### Glioblastoma organoid culture

Glioblastoma organoids (GBO) for co-culture experiments were derived as follows: UP-O-9121 was derived from a 64-year-old male, harboring EGFR copy number gain and EGFRvIII mutation. UP-O-11390 was derived from a 60-year-old female, harboring EGFR copy number gain (University of Pennsylvania IRB protocol #816686). Race and ethnicity were not reported for these two patients. GBOs were generated and maintained as previously described,^97^ with minor modifications: Freshly resected glioblastoma specimens were immediately placed in a cold dissection medium consisting of Hibernate A supplemented with GlutaMax and Antibiotic-Antimycotic (Thermo Fisher Scientific) and transported to the laboratory. Upon arrival, tumor tissue was mechanically minced into approximately 1 mm^3^ fragments and subjected to red blood cell lysis using 1× RBC lysis buffer (Thermo Fisher Scientific), followed by washing with Dulbecco’s phosphate-buffered saline (DPBS). Tissue fragments were transferred to six-well plates containing GBO culture medium composed of a 1:1 mixture of Neurobasal and DMEM/F12 supplemented with non-essential amino acids, GlutaMax, penicillin/streptomycin, B27 supplement without vitamin A, N2 supplement, 2-mercaptoethanol, and 2.5 μg/mL recombinant human insulin. Organoids were maintained on an orbital shaker at 110 rpm in a humidified incubator at 37°C with 5% CO_2_.

### METHOD DETAILS

#### CSF sample preparation for scRNA-seq

CSF samples were drawn from patients via an Ommaya reservoir. The cellular fraction of the CSF was cryopreserved in 1 mL of 50% Fetal Bovine Serum, 40% RPMI media, and 10% dimethyl sulfoxide (DMSO) and stored in liquid nitrogen until ready for single cell library preparation. CSF cells were thawed in a 37°C water bath and diluted in warm RPMI-1640 media (Thermo Fisher 11875093) containing 10% FBS and 10 μg/mL DNAse. Cells were counted via hemocytometer. If red blood cells are present in the sample, red blood cell depletion was performed using the STEMCELL EasySep RBC depletion reagent (#18170) and STEMCELL EasyPlate Magnet (#18102).

For patients 1–6, cells were counted via hemocytometer from a volume of 0.5 mL PBS. Cells were then resuspended in a volume calculated to give an appropriate loading concentration per 10x Genomics Chromium GEM-X Single Cell 5’ Reagent Kits v3 protocol (CG000733 Rev A). For patients 7–18, cells were counted in approximately 20 μL PBS and loaded into a hemocytometer at a dilution of 1:5. After counting, cells were loaded per 10x Genomics protocol. Counting in a low volume and avoiding subsequent resuspension increases counting accuracy and improves cell recovery. As a result, cell recovery was substantially higher for these patients. In total, CSF was withdrawn from 18 different patients, and all panels related to patient CSF use data from these patients.

#### CAR T product preparation for scRNA-seq

CAR T cell infusion products are produced by the University of Pennsylvania Clinical Cell and Vaccine Production Facility. CAR T cells were cryopreserved and stored in liquid nitrogen until ready for use. Prior to single cell library preparation, cells were thawed in a 37°C water bath and diluted with warm RPMI-1640 media (Thermo Fisher 11875093) containing 10% FBS and 10 μg/mL DNase. Cells were then stained for viability with Calcein AM (Thermo Fisher C1430) at a working concentration of 1 μg/mL and TO-PRO-3 (T3605) at a working concentration of 0.33 μM. Cells were then sorted for viability on an SH800S cell sorter system (Sony). Only cells staining positive for Calcein AM and negative for TO-PRO-3 were accepted. After sorting, cells were centrifuged at 400g for 5 minutes to concentrate to a volume of 400–2000 cells/μL. Concentration was confirmed via hemocytometer. After measuring concentration, an appropriate volume of cells was loaded into a 10x Genomics Chromium X device per GEM-X Single Cell 5’ Reagent Kits v3 protocol (CG000733 Rev A), targeting a recovery of approximately 8000 cells.

#### Tumor sample preparation for snRNA-seq

Tumor biopsies before and after CAR T therapy were processed by mechanical dissociation using a gentleMACS Octo Dissociator (130–093-235) and then filtered through a 75-μm strainer to generate a single-cell suspension. Samples were cryopreserved in a base media of DMEM/F-12 (Millipore Sigma D8437) supplemented with 5% B-27 supplement (Thermo Fisher 17504044) and 1% GlutaMax supplement (Thermo Fisher 35050061). Cryomedia was prepared as 90% base media and 10% DMSO. Tumor cells were suspended in cryomedia and stored in liquid nitrogen until further use. For single nucleus preparation, tumors were thawed and nuclei isolated based on the protocol from Spitzer et al.,^98^ in which dissociated tissue was incubated in a lysis buffer consisting of 0.49% CHAPS, 0.1% bovine serum albumin, 10 mM Tris, 146 mM NaCl, 1 mM CaCl_2_, and 21 mM MgCl_2_ with trituration for 10 minutes or until homogenous. Lysed suspension was then strained through a 40 μM filter. Isolated nuclei were stained with DAPI (Thermo Fisher D1306) at working concentration of 10 μg/mL. Nuclei suspensions were sorted for high-quality DAPI-positive nuclei using an SH800S cell sorter system (Sony). Sorted nuclei were centrifuged at 500g for 5 minutes to concentrate to a range of 200–2000 nuclei/μL and counted using a hemocytometer. After measuring nuclei concentration, an appropriate volume of nuclei was loaded into a 10x Genomics Chromium X device per GEM-X Single Cell 5’ Reagent Kits v3 protocol (CG000733 Rev A). In total, paired pre and post-infusion tumor samples were obtained from six different patients (*n* = 6), and all panels related to patient tumor results use data from these patients.

#### CAR detection by cytometry

Identification of CAR+ T cells in the peripheral blood and the infusion product was performed on single-cell suspensions using spectral cytometry. Briefly, thawed cells were resuspended in PBS-FBS 2% and then incubated with either EGFR or IL13Rα2 biotinylated proteins for 20 min at 4 °C before further staining with surface and intracellular antibodies as described below (Data S1).

#### Spectral cytometry

Thawed single-cell suspensions of infusion products were assessed using CyTek. Briefly, antibody panels (T-cell panel) were designed to simultaneously measure the expression of molecules related to cell lineage, differentiation state and function. For T cell memory subpopulations by flow cytometry after staining with mAbs specific to CD14, CD19, CD3, CD45RA, CD27, CCR7 (Biolegend). Cells (3 × 10^6^) were stained at 4 °C for 20 min with fluorochrome-labeled antibodies to detect surface proteins. Cells were then permeabilized using Fixation/Permeabilization solution (Thermo Fisher, 00–5523-00) at room temperature for 15 min and then stained at 4 °C for 30 min with fluorochrome-labeled antibodies to detect intracellular proteins. Cells were washed in permeabilization buffer and resuspended in PBS, FBS 2% paraformaldehyde before acquisition by CyTek. CD45+ cells were identified as LiveDeadnegSingleCD45+. Data were analyzed using OMIQ (https://www.omiq.ai/) and FlowJo 10.10.0 software (Becton, Dickinson and Company). Gating was performed as described in Data S1. All comparisons between Responder and Non-Responder conditions were performed using unpaired t-tests resulting in p > 0.05.

#### scRNA-seq and snRNA-seq library preparation

Single-cell and single-nucleus V(D)J and gene expression libraries were made from CSF, infusion product, and tumor samples using the GEM-X Single Cell 5’ Kit, which includes the Chromium GEM-X Single Cell 5′ Kit v3 16 rxns (PN-1000699), Library Construction Kit C 16 rxns (PN-1000694), Chromium GEM-X Single Cell 5′ Chip Kit 4 chips (PN-1000698), Chromium Single Cell Human TCR Amplification 16 rxns (PN-1000252), and Dual Index Kit TT Set A 96 rxns (PN-1000215). CSF samples were loaded into the 10x Chromium X as described above, and subsequent steps were performed according to the 10x Genomics Chromium GEM-X Single Cell 5’ Reagent Kits v3 protocol (CG000733 Rev A). Library quality control was performed using the Agilent Technologies Tapestation and High-Sensitivity D5000 Screentape (5067–5592).

#### Illumina sequencing of sc/snRNA-seq

Sequencing of single-cell CSF and infusion product and single-nucleus was performed by the Children’s Hospital of Philadelphia High Throughput Sequencing Core facility on an Illumina Novaseq 6000 or Novaseq X (Illumina) sequencer. Libraries were sequenced in paired-end mode at a targeted minimum depth of 60,000 reads per cell for gene expression 15,000 reads per cell for V(D)J sequences using a NovaSeq S1 or S2 reagent kits (version 1.5, 100 cycles) or a NovaSeq X 1.5B or 10B read kit (100 cycles), depending on the number of cells sequenced. The run geometry was 28 × 10 × 10 × 90. Libraries were quantified by the sequencing core using the Roche KAPA quantitative PCR kit (KK4824).

#### Bulk RNA-seq from FFPE tumor samples

Hematoxylin and eosin (H&E)-stained slides from formalin-fixed, paraffin-embedded tumor samples were analyzed to identify regions rich in malignant cells. Tissue from these regions was collected by laser capture microdissection using a Leica LMD7000 microscope and tissue sections on PEN membrane slides (Leica, 11505158). To ensure acquisition of sufficient tissue, multiple sections were collected per block, depending on area collected and estimated RNA yield. The average RNA yield was 2.09 ng/mm^2^. RNA was collected from dissected tissue using the RNeasy FFPE Kit (Qiagen, 73504). RNA quality was assessed using a Tapestation (Agilent) with High-sensitivity RNA reagents (Agilent, 5067–5579). Transcriptome libraries were generated from RNA by the Penn Genomics and Sequencing Core using the SMART-Seq^®^ Total RNA Pico Input with UMIs (ZapR^™^ Mammalian) (Takara, 634354) with Unique Dual Index Kit (1–24) (Takara, 634756). Libraries were sequenced on a NextSeq 2000 (Illumina) with a P3 sequencing kit at an approximate depth of 100 million reads per sample.

#### GBO Generation and Co-culture

Co-culture experiments were performed using GBOs derived from patients as described above. (See experimental model and study participant details). On D1, individual organoids were placed into single wells of 96-well U-bottom plates containing 200 μL of GBO culture medium. Dual-targeting CAR T cells were thawed and activated in ImmunoCult^™^-XF T Cell Expansion Medium supplemented with penicillin/streptomycin and 30 IU/mL recombinant human IL-2.

On D2, 10,000 CAR T cells or an equivalent number of untransduced T cells (UTD) were added per well, corresponding to an approximate effector-to-target (E:T) ratio of 1:10. NK cells were thawed in RPMI-1640 media with 10% FBS and 100 IU/mL recombinant human IL-2. To preserve viability and cytotoxicity, NK cells had been treated with IL-15 and IL-18 at 50 and 250 ng/mL, respectively, 24 hours prior to cryopreservation as previously described.^99^ On D3, 7,000 thawed NK cells were added per well, achieving an approximate 1:1 ratio with CAR T cells after accounting for T-cell proliferation. Six experimental conditions were evaluated: GBO alone, GBO + CAR T cells, GBO + UTD cells, GBO + NK cells, GBO + CAR T cells + NK cells, and GBO + UTD cells + NK cells.

Culture medium was replaced daily, with spent medium collected for conditioned media analysis. Organoids and co-culture samples were harvested on D6 and fixed for downstream immunohistochemical analysis.

#### Immunohistochemical Staining and Analysis

GBOs and co-cultures were fixed, dehydrated, and cryosectioned into serial 20 μm sections using a Leica cryostat (Deer Park, IL). Sections were mounted onto charged glass slides (Thermo Fisher Scientific), air-dried at room temperature, and stored at −20°C until use. Prior to immunostaining, sections were outlined using a hydrophobic barrier pen (Vector Laboratories) and rinsed with Tris-buffered saline (TBS) containing 0.1% Tween-20.

For immunofluorescence staining, sections were permeabilized and blocked for 1 hour at room temperature in blocking solution containing 10% donkey serum, 0.5% Triton X-100, 1% bovine serum albumin, 0.1% gelatin, and 22.52 mg/mL glycine in TBST. Sections were incubated overnight at 4°C with primary antibodies diluted in TBST supplemented with 5% donkey serum and 0.1% Triton X-100. Primary antibodies included anti-CD3 (mouse monoclonal, BioLegend, #344802, 1:500), anti-cleaved caspase-3 (rabbit polyclonal, Cell Signaling Technology, #9661S, 1:1000), and anti-CD45 (rat monoclonal, Santa Cruz Biotechnology, #sc-65344, 1:500).

Following primary antibody incubation, sections were washed with TBST and incubated for 1.5 hours at room temperature with species-appropriate donkey-derived secondary antibodies (Thermo Fisher Scientific) and DAPI (Sigma-Aldrich), diluted 1:500 in TBST containing 5% donkey serum and 0.1% Triton X-100. Sections were rinsed with DPBS, mounted with commercial mounting medium (Vector Laboratories), coverslipped, and sealed.

Confocal images were acquired using a Zeiss LSM 710 microscope with Zen 2 software (Zeiss). Quantitative image analysis was performed using ImageJ Fiji (v2.9.0) to measure mean cleaved caspase-3 signal intensity following background subtraction.

For immunohistochemical staining of patient FFPE tissue slides, sections were processed according to manufacturer’s protocol. Staining was performed on a Leica Bond-PRIME instrument using the Bond Prime Polymer Refine Detection System (Leica Cat# DS9284). Heat-induced epitope retrieval was performed using Bond Prime Epitope Retrieval 1 (Leica, Cat # AR0086) or Bond Prime Epitope Retrieval 2 (Leica, Cat# AR0087). Primary antibodies were anti-CD3 (Leica, Cat# PA0553), anti-CD68 (Dako, Cat# IR609), and Ki67 (Dako, Cat# IR62661),

#### Cytokine quantification

GBO co-culture supernatants from D1, D3, and D5 were collected, pooled, and stored at −20 °C until use. Samples were thawed on ice and centrifuged at 1000 × g for 10 minutes at 4°C to remove any cells or particulates prior to processing. The assay was performed according to the manufacturer’s protocol, using the MACSPlex Cytokine 12 Kit, human (Miltenyi Biotec, 130–099-169). Samples were not diluted and were run in triplicate on a 96-well filter plate. Sample acquisition was performed using a MACSQuant Analyzer 10 Flow Cytometer with the automated Express Mode setting, and the data were analyzed using the MACSQuantify software (Miltenyi Biotec).

#### Soluble CD27 quantification

Patient CSF samples from D0 (pre-infusion) and D7 (post-infusion) were thawed on ice and gently pipette-mixed before processing. Aside from the predilution step, the assay was performed according to the manufacturer’s protocol using the Human sCD27 Instant ELISA Kit (Invitrogen, BMS286INST) which allows determination of the concentration of soluble CD27 in a sample in a Microwell Plate. CSF samples were not prediluted prior to addition to the Microwell Plate unless at a low volume. These samples were diluted appropriately so there was enough volume to perform the assay, and later concentrations were multiplied by the dilution factor accordingly. All samples were processed in technical duplicates; absorbance was measured at 450 nm on a Synergy H4 Hybrid Reader (BioTek) and normalized to the blank using the Gen5 software program. sCD27 concentration was calculated according to the manufacturer’s protocol, using a standard curve ranging from 0 U/mL to 20 U/mL.

#### RNAscope

In situ hybridization was performed using a Leica BOND RXm automated platform using the RNAscope technology (RNAscope^™^ 2.5 LS Probe- Hs-EGFR (Cat No.310068)). The specific chromogenic detection kit (ACD #322150) was implemented with a Leica BOND RED Detection System (DS9390). Slides were counterstained in hematoxylin, air-dried, and permanently mounted with EcoMount (Biocare EM897L).

The slides were then scanned using the Leica Versa 200 whole slide scanner at 40x magnification and images were visualized using the ImageScope software. Digital image analysis was performed by tuning a Positive Pixel Count algorithm available on the ImageScope software. Briefly, regions of interest were annotated on the scans and used for the analysis. The algorithm was tuned to detect pixels considered positive by the pathologist. The ratio of positive pixels to the total number of pixels (i.e., Positivity) was calculated by the software.

### QUANTIFICATION AND STATISTICAL ANALYSIS

#### FFPE bulk RNA-seq alignment and processing

Fastq files were first trimmed using umi_tools^96^ (v1.1.6) extract, trimming the first 8 bases of read 1, containing the UMIs and appending the UMI to the read name. Cutadapt^100^ (v5.2) was then used to trim 14 bp from the beginning of read 2 to remove non-biological sequence introduced during library preparation. Reads were then aligned to a custom version of the hg38 genome to which the CAR transcript was appended using the STAR aligner^95^ (v2.7.9a) with the settings -outSAMtype BAM SortedByCoordinate and –outSAMattributes NH HI AS nM. Resulting BAM files were indexed using samtools (v1.23)^101^ index. Duplicate reads (such as PCR duplicates) were removed from the indexed BAM file based on UMI using umi_tools (v1.1.6) dedup. Reads were counted using featureCounts^102^ (v2.1.1). Ensembl gene IDs were collapsed to human gene symbols using biomaRt^103^ (v2.58.2). CPM values were computed after TMM normalization with CalcNormFactors (edgeR v4.0.16).^93^

#### Single Cell RNA Sequencing data analysis

Code for generating the results presented in this study is available on the Silverbush lab GitHub; see the data and code availability section.

##### CSF/infusion Cell Ranger with CAR reference

FASTQ files were processed using Cell Ranger Multi v8.0.1 pipeline (10x Genomics). To specifically detect CAR transcripts, gene expression data were aligned and quantified against a custom reference genome generated by appending the full nucleotide sequence of the bicistronic EGFR-806/IL13Rα2 CAR handle to the standard human reference genome (GRCh38; refdata-gex-GRCh38–2020-A) using the cellranger mkref pipeline. Given the use of 5′ library capture, detection relies on the unique sequence signature at the 5′ end of the construct. Because the CAR utilizes a human leader sequence, specificity is ensured by reads spanning the junction between the signal peptide and the unique proximal transgene sequence. The 91-nucleotide sequence corresponding to this region is: ATGGCCCTCCCTGTCACCGCCCTGCTGCTTCCGCTGGCTCTTCTGCTCCACGCCGCTCGGCCCGATATTCTGATGACTCAATCTCCGTCTT. TCR data were aligned and quantified against the GRCh38 Ensembl v7.1.0 reference. All pipeline steps were executed with default parameters unless otherwise specified.

##### Tumor Cell Ranger and Seurat preprocessing

Fastq files from single-nucleus gene expression and V(D)J libraries were uploaded to the 10x Genomics Cloud analysis platform for alignment using Cellranger Multi v8.0.1. Gene expression data were aligned to the custom reference genome based on GRCh38-2020-A described above, and V(D)J data were aligned to the GRCh38 Ensembl v7.2.0 reference. After alignment, gene expression data was subjected to preprocessing Cellbender^74^ v0.3.0 to remove signal from ambient RNA. Count data processed by Cellbender were analyzed by the Python tool Scrublet v0.2.3 to identify doublets. Gene expression data was further pre-processed in Seurat v5.2.1.^104^ Cells were first filtered to exclude doublets called by Scrublet, then cross-referenced with cells passing the Cellranger quality filter to exclude any cells not passing the filter. Cells were manually filtered to exclude probable doublets and empty droplets based on per-cell RNA counts and gene counts. Finally, cells with more than 10% mitochondrial genes were excluded. Tumor cell type annotations were done similarly as in CSF.

##### Ambient RNA correction

Ambient RNA contamination for CSF, tumor, and infusion product samples was removed from the raw feature-barcode matrices using CellBender Remove-Background^74^ v0.3.0. For each CSF and infusion product sample, the unfiltered UMI count matrix produced by Cell Ranger was supplied to CellBender, and the model was run with a false-positive rate of 0.1 to conservatively estimate ambient RNA profiles and subtract them from cell barcodes; all other parameters were left at their defaults. The resulting decontaminated count matrices were carried forward into all downstream analyses. For tumor samples, CellBender was run with a false-positive rate of 0.0 for most conservative signal removal, given the higher heterogeneity and potential low RNA content of some tumor nuclei. Suspected empty droplets were subsequently removed through manual filtering based on UMI and gene count distributions, as described in the quality control section. This two-step approach allowed us to retain legitimate nuclei while effectively eliminating low-quality droplets.

##### Quality control

Single cell count matrices were subjected to quality control using Seurat v5.1.0 and Scanpy v1.9.1. CSF and infusion product cells with fewer than 500 detected genes, fewer than 1,000 UMIs, or more than 50,000 UMIs were excluded, as were any cells exhibiting over 10% mitochondrial transcript content. For tumor samples, custom exclusion criteria were created for each sample to filter empty droplets based on distribution of detected UMI and gene counts. Cells with more than 10% mitochondrial genes were excluded. To identify and remove multiplets, Scrublet was run on each sequencing batch with an expected doublet rate of 6%; barcodes flagged as doublets were discarded. Quality and filtering metrics are included in Table S6.

##### Dimensionality reduction & batch correction

Following QC and normalization (log-transformed counts scaled to 10,000 UMIs per cell), we defined a global set of highly variable genes (HVGs) by first selecting the top 4,000 (2000 for tumor samples) most variable genes within each individual sample and then taking the union of these lists across all samples. To ensure the embedding reflected meaningful biology rather than clonotype-specific or proliferative signals, we removed from this union all TCR variable segments, immunoglobulin variable genes, and any genes whose variance was predominantly driven by cell-cycle phase. The resulting HVG set was centered and scaled. Principal component analysis on these features yielded 50 principal components that captured the majority of transcriptomic variance. To align cells across library processing dates, we applied Harmony v1.2.0^75^ to all 50 principal components, using processing date as the batch covariate. The Harmony-adjusted embeddings were then used to construct a shared nearest-neighbor graph and to define clusters via Louvain community detection (resolution = 0.5). For visualization, UMAP was performed on the 50 Harmony-corrected principal components. Tumor samples were found to have minimal batch effects, as all samples were sequenced in one sequencing experiment. Therefore, batch correction was not performed on tumor samples.

##### Statistical analysis

Unless stated otherwise, comparisons of groups were assessed for statistical significance with a two-sided Wilcoxon rank-sum test for unpaired analyses and a two-sided Wilcoxon signed-rank test for paired analyses. For all statistical analyses, p values less than the threshold of 2.22 × 10^−16^ are printed as *p* < 2.22E−16, and all other p values are printed as the exact value.

##### Cell type annotation

For CSF and infusion product cells, CellTypist^76^ (v1.6.3) algorithm was first used to assign preliminary identities, with the *Immune_All_Low* and *Immune_All_High* reference models to establish preliminary immune-subset identities. These automated annotations were manually refined by evaluating canonical marker genes and gene signature scores, resulting in the delineation of four major cellular compartments: B cells, plasma cells, T/NK cells, and myeloid cells. B and plasma cell populations retained their preliminary annotations without additional reclustering. Cells from the T/NK and myeloid compartments were extracted and separately reclustered as described above. Clusters exhibiting robust co-expression of markers from multiple lineages were identified as putative doublets and subsequently excluded based on verification of higher mean doublet scores (assigned by Scrublet) and elevated numbers of UMIs relative to other clusters. Final refined cell-type annotations for each cluster were assigned based on canonical lineage- and subset-specific marker expression, as illustrated in Figure S1E, and these annotations were then integrated into the main dataset for subsequent analyses.

For tumor tissue samples, malignant cells were identified using inferred copy number alterations (CNAs) relative to non-malignant populations (as shown in Figure S4C). After removing immune and non-malignant cells, remaining tumor cells were subclustered in Seurat, and the FindAllMarkers function was used to identify genes enriched in each cluster. Canonical genes for each cell type were used to manually annotate cells. Lymphoid and myeloid were each sub-clustered to distinguish cell types within these groups, and FindAllMarkers was used again to identify cell types within each sub-cluster (Figure S4B).

##### Malignant cell state annotation

Malignant states in tumor single-nucleus data were scored and annotated as described in Neftel et al.^41^: Cells were scored with gene signatures associated with each cell state using AddModuleScore (Seurat v5.2.1). Cells were then first assigned a stemness score based on the larger of their score for NPC/OPC signature (stem-like) or AC/MES (differentiated-like). Cells deemed stem-like are further categorized as either NPC or OPC based on the larger module score. Differentiated-like cells are likewise categorized by their AC and MES score.

##### Myeloid Program Characterization in CSF

Myeloid cells were analyzed following the cNMF pipeline as previously described.^56^ 87k Myeloid cells were selected from all patient samples (44) with leiden clustering (resolution = 0.1). The top 2000 variable genes were selected with num_var_genes (scanpy v1.10.3) after filtering for 0.01 mean gene expression and 0.1 expression in 0.1% of all cells. cNMF was then run (https://app.terra.bio/#workflows/mparikh/cnmf_parallel/7). For cNMF ‘prepare’, we performed factorization over k = [4, 26], using parameters – numgenes 2000; –num_iter 500; –density 0.02. We selected K = 14 (silhouette score > 0.8) and annotated the 14 programs with enrichment scores from running gProfiler with a custom gene set list. We identified a T cell program within the 14 programs, which we considered to be the result of myeloid-T cell doublets. To remove these doublets, we filtered out myeloid cells with high usage of this T-cell program (>30%). We then re-ran cNMF on this cohort of cleaned cells. 2000 variable genes were again selected for the 81k myeloid cells and cNMF was re-ran as described above. We identified and annotated 12 myeloid programs. There was a T cell program which was restricted to less than 0.1% of cells so we removed it for downstream analysis. The final ‘usage’ matrix output of cNMF was used for downstream analysis. For each cell, the usage scores were normalized to 100%. For each sample, the number of cells with a normalized usage score for each program > 20% was calculated then normalized by the total number of cells in the sample. This creates a percentage of cells using each program at a sample level.

The baseline sample for patient 10 (P10D0) was calculated separately due to sequencing timing issue, and the usage scores for the replacement samples were calculated with the myeloid programs gene-spectra file. We filtered the raw counts matrix to genes in the spectra file and then used the cNMF prepare script to normalize the raw counts matrix. We used sklearn.decomposition.non_negative_factorization where X is the filtered normalized expression matrix and H is the gene-spectra matrix. The following parameters were used: n_components= 14, init=‘random’, update_H=False, solver=‘cd’, beta_loss=‘frobenius’, tol=0.0001, max_iter=1000, alpha=0.0, alpha_W=0.0, alpha_H=‘same’, l1_ratio=0.0, regularization=None, random_state=None, verbose=0, shuffle=False. The usage scores were normalized to 100% for each cell.

##### Use of cNMF programs in myeloid cells

Rather than performing de novo cNMF on cells from GBM tissue, we utilized the consensus myeloid programs previously defined,^56^ as these represent more robust programs than what could be determined from 6 samples. To do this, we selected the 31,593 myeloid cells and their 36,602 identified genes from all CAR-infusion tumor tissue samples (*n* = 6). These genes were filtered to the list of genes intersecting the genes in the reference spectra from the 14 myeloid programs found in Miller et al.^56^ To “back-calculate” the usages of the 14 programs, factorization of the myeloid cell-gene matrix was performed using the non_negative_matrix_factorization from the sklearn package, solving for the cell-program matrix using the same parameters as during CSF myeloid program factorization. This cell-program matrix was then row-normalized to determine program usages on a 0–100 scale.

##### Cell type composition analysis

We applied the single-cell compositional data analysis framework (scCODA,^77^ v0.1.9) to evaluate changes in immune subset frequencies between D0 and D7. To avoid confounding by the infused product, CAR-positive clusters were excluded prior to quantification. For each patient at each time point, the relative abundance of the remaining annotated cell types was computed. To account for the compositional nature of these data, scCODA fits a Bayesian linear regression with a Dirichlet–Multinomial likelihood, automatically selecting a reference population whose relative abundance exhibits the lowest dispersion across samples and is detected in ≥ 95 % of all specimens. In our D0 versus D7 analysis, B cells were chosen as the reference by this automated selection method. Parameter inference was carried out via Hamiltonian Monte Carlo sampling (20,000 iterations), and differential abundance was deemed significant at a false discovery rate of 0.1.

To evaluate the impact of steroid administration on cell type composition, we performed a separate scCODA analysis, within the low-dose cohort alone. The high-dose cohort was excluded because all high-dose patients received similar steroid exposure consistent with trial eligibility criteria which mandated a low-dose corticosteroid window prior to infusion and homogeneous steroid treatment post-infusion in the high-dose cohort. In contrast, steroid use in the low-dose cohort was heterogeneous, where half of the patients received supportive care only. We use scCODA to model the interaction between steroid use and time point (Steroid × D7) which allows us to identify cell subsets where the magnitude or direction of proportion changes between D0 and D7 was significantly dependent on steroid exposure. The same reference population selection method, inference parameters and FDR as above were applied.

##### Cell type-specific differential expression

To characterize transcriptional dynamics across time points, clinical (responder vs non-responder), and compartmental (infusion product vs CSF, expanded Treg vs nonexpanded Treg, etc.) comparisons, we employed a pseudo-bulk differential expression workflow using muscat^78^ (v1.16.0). Briefly, raw UMI counts were aggregated per gene for each sample–cell-type combination. To ensure reliable variance estimation, any sample–cell-type pair containing fewer than ten cells was excluded, and muscat’s internal filters for low-quality samples and lowly expressed genes were applied. Differential expression between groups within each cell type was assessed through muscat’s pbDS workflow using the edgeR method: for each gene, a negative-binomial generalized linear model with the group variable as predictor was fitted, and quasi-likelihood F-tests (glmQLFTest) were performed to evaluate significance. Results were subsequently filtered to retain only protein-coding genes based on Ensembl^105^ biotype annotations. Genes with nominal *p* < 0.05 and absolute fold-change > 2 were deemed differentially expressed and reported in Table S3. For tumor samples, cells were subsetted to malignant cells. Due to the low number of available paired samples, differential analysis was performed cell-wise using the FindMarkers tool with logistic regression (“LR”) from Seurat (v 5.2.1), comparing post-CAR to pre-CAR. Only genes expressed by a minimum of 20% of cells in either group were tested, and per-cell library size was regressed out during analysis. Multiple comparisons were controlled with the Bonferroni method.

##### Gene set enrichment analysis

Gene set enrichment analysis was performed in R using the fgsea package^79^ (v1.28.0, CSF and infusion product) or clusterProfiler^94^ (v4.10.1, tumor). Genes were pre ranked by average log2 fold-change from the pseudo-bulk differential expression workflow (CSF and infusion product) or cell-wise differential expression (tumor) described above and sorted in descending order. Adaptive permutation testing produced normalized enrichment scores (NES) that account for gene-set size, and multiple testing was controlled by the Benjamini–Hochberg procedure (FDR < 0.05). For the D7 CSF analysis, CAR-positive and CAR-negative CD8^+^ T cell clusters were evaluated against a 243-gene program originally defined in metastatic human tumors as the core transcriptional module of CD8 neoantigen-reactive TILs.^37^ The running-sum enrichment score was plotted across the full ranked gene list to illustrate signal accumulation and leading-edge analysis identifying the subset of signature genes driving peak enrichment was applied, and the core drivers of enrichment were reported. To compare CD8^+^ CAR T cells in the infusion product with those in CSF, we tested for enrichment of MSigDB (v7.5.1) Gene Ontology Biological Process gene sets between the two compartments. Selected significantly enriched GOBP pathways were depicted as bar plots.

##### Functional Module Enrichment Analysis

To evaluate cellular functional states, we curated well-defined gene signatures for cytotoxicity and exhaustion from published sources.^33,34^ Human-mouse gene mapping was based on the HomoloGene database (https://www.ncbi.nlm.nih.gov/homologene). Percell expression values were first normalized and log-transformed as described above. We then applied the UCell (v2.6.2)^80^ algorithm, which ranks all genes by expression within each cell and computes an enrichment score based on the empirical cumulative distribution of signature genes among those ranks. This yields a continuous score for each module in every cell. For each signature, we compared the distribution of per-cell scores between the groups of interest using two-sided Wilcoxon rank-sum tests.

##### TCR clonotype assignment & diversity analysis

Cell Ranger V(D)J outputs were imported into R using scRepertoire^81^ (v2.3.4), and productive α- and β-chain contig tables were merged across all samples with stringent quality filtering: barcodes lacking either chain, containing nonproductive contigs, or exhibiting more than two chains were excluded; in cases of multiple contigs per chain, only the sequence with the highest UMI count was retained. Unique clonotypes were then defined by paired CDR3 amino-acid sequences of the α and β chains, and each cell was annotated with its clonotype ID and clone size (the number of cells sharing that ID). Clonal frequency (pᵢ) was computed as the fraction of T cells assigned to each clonotype within a sample. Shannon entropy (*H* = −∑i p_i_ ln p_i_) was calculated after downsampling each sample’s T-cell pool to match the size of the smallest repertoire. Entropy estimates were stabilized via 1,000 bootstrap replicates. Clonality was defined as the complement of Pielou’s evenness (*C* = 1 - *H* / ln *S*), where *S* is the number of unique clonotypes. Clonality values range from 0 to 1, where higher values indicate greater dominance of expanded clones, whereas values closer to 0 reflect a more even, polyclonal landscape.

##### Longitudinal tracking of TCR clonotypes

Utilizing the clonotype assignments described above, we characterized the transcriptional evolution of persisting CD8 T cells. Analysis was restricted to clonotypes present at all three time points: baseline (Infusion Product for CAR+ clones or D0 for CAR- clones), D7, and D21. These persisting clones were stratified into CAR-positive and CAR-negative based on CAR transcript detection. For each shared clonotype, the mean expression of select genes was calculated at each timepoint. Trajectories for each gene were classified based on the directionality of expression changes between sequential intervals (Baseline to D7 and D7 to D21). Patterns that were both frequent (comprising >10% of the persisting repertoire) and statistically significant (paired Wilcoxon signed-rank test *p* < 0.05) were highlighted.

##### Corr immune composition\clonality with outcome

To evaluate whether cell-type frequencies or TCR clonality correlated with clinical outcomes, we computed Pearson’s correlation coefficient (r) against two endpoints: progression-free survival (PFS) and percent change in tumor size from baseline by RANO criteria. Tumor size change was defined as the maximum percentage change in the sum of products of the longest perpendicular diameters of target lesions, as specified by the RANO guidelines. One patient lacking measurable disease at infusion (enhancing lesion <1 cm × 1 cm) was excluded from tumor-size analyses. PFS was defined as the interval from CAR T infusion to either radiographic progression (per RANO) or death. For each immune-based predictor, we report the Pearson correlation coefficient and the two-sided p-value for the null hypothesis of zero correlation; nominal significance was set at *p* < 0.05.

##### Infusion Product and Infiltration Analysis

Analysis of infusion product cells and response status included the above cell type composition analysis with scCODA, functional module enrichment analysis with UCell, and *Cell type-specific differential expression analysis* with muscat and edgeR. In addition to cell-type-specific DGE, to compare gene expression overall while controlling for cell type in infusion products of responders and non-responders, gene expression was aggregated to the patient level, and DGE was conducted via edgeR with cell type modeled as a fixed effect.

To investigate features of infused T cells which infiltrated tumor tissue, TCR-based clonotype matching was performed to identify TCR-matched clones between infusion product and post-infusion tumor samples. Infusion product cells with clonotypes detected in the post-infusion tumor were labeled “TCR-matched observed,” and all other infusion product cells were labeled “Non-matched.” Samples with fewer than ten “TCR-matched observed” cells were excluded from downstream analysis.

Given the scarcity of “TCR-matched observed” cells, it was necessary to rely upon Monte Carlo hypothesis testing for differential gene expression analysis using the MMCtest algorithm from the simctest package (v2.6.1) with default testing parameters.^88^ Protein-coding genes detected in at least ten cells and with total counts across all cells of at least 20 were retained. A test statistic was defined as the log2 difference between pseudobulk expression in all *n* “TCR-matched observed” cells and the mean pseudobulk expression of 100 random samples of *n* “Non-matched” cells. Random permutation of the “TCR-matched observed” label permitted generation of a null distribution from which FDR-corrected p-values were estimated.

Gene set enrichment was carried out with gprofiler2 (v0.2.3) using custom background set to the filtered input gene list, and an ordered query ranking genes by the log2 fold change defined above.^89^ A fold enrichment score was calculated as log2[(intersection size × effective domain size) / (term size × query size)].

##### Differential TF activity for T cells Analysis

To elucidate activity of known transcription factor programs, regulons were selected from the DoRothEA database (v1.20.0) of human transcription factor regulons with confidence levels A, B, or C.^90^ The Virtual Inference of Protein-activity by Enriched Regulon (VIPER, v1.42.0) scale method was used to assign TF activities to cells.^91^

For infusion product-response comparisons, TF activity was aggregated to the patient level, and differential TF activity analysis was performed via limma (v3.66.0) for continuous normalized data, with cell type modeled as a fixed effect.^92^ Cell-type-specific analysis was performed with muscat’s pbDS method using limma. For the infiltration analysis, differential TF activity analysis was carried out via Monte Carlo hypothesis testing with the MMCtest method as described for gene expression above.

##### Kaplan–Meier & exact log-rank tests

Progression-free survival was defined as above and patients without an event were censored at last follow-up. Patients were dichotomized at the median change in the percentage of CD56 ^dim^ CD16 ^pos^ NK cells among total NK cells (D7 minus D0) into “low-change” and “high-change” groups. An exact permutation–based log-rank test was used to compare progression-free survival between these cohorts. Kaplan–Meier survival curves were estimated separately for the low-change and high-change groups with numbers at risk shown at regular intervals below the plot. Median progression-free survival estimates were calculated and reported in the text.

##### Ligand-receptor analysis

Ligand-receptor analysis was performed using the LIANA R package^82^ (version 0.1.14). LIANA was run using the default settings on each sample. Then, the predictions from all samples were combined. All predicted ligand-receptor interactions were considered without filtering to make sure key interactions were not missed. The strength of an interaction is represented by SingleCellSignalR’s LRScore,^106^ and the specificity of an interaction is represented by NATMI’s edge specificity.^107^ Missing values were imputed with zero. CTLA4, while classified as a receptor in LIANA’s framework, was treated as a ligand in our analysis to reflect its biological role in initiating suppressive signaling upon binding to CD80/CD86 on antigen-presenting cells. This adjustment allowed us to better interpret Treg-mediated suppressive interactions in the CSF. Monocytes, macrophages, microglia, and BAMs were grouped together for the analysis for convenience of interpretation. Only cell types of interest were plotted, and the top 1000 predicted ligand-receptor interactions ranked by LIANA can be found in Table S5. Cytotoxicity signature was generated as described above in functional module enrichment analysis.

### ADDITIONAL RESOURCES

This work relies on clinical samples generated from a clinical trial (NCT05168423), which can be accessed at https://clinicaltrials.gov/study/NCT05168423.

## Supplementary Material

1

2

3

4

Supplemental information can be found online at https://doi.org/10.1016/j.cell.2026.05.026.

## Figures and Tables

**Table 1.** Cohort overview

Cohort	Dose (CAR T cells)	Patient IDs	Tumor tissue
High-dose discovery	1–2.5 × 10^7^	P1–P8	P1, P4, P7
High-dose validation	1–2.5 × 10^7^	P9–P12	–
Low-dose cohort	5 × 10^6^	P13–P18	P13, P14, P16

**Table:** KEY RESOURCES TABLE

REAGENT or RESOURCE	SOURCE	IDENTIFIER
Antibodies		
Mouse monoclonal anti-human CD3 (SK7)	BioLegend	Cat# 344802; RRID:AB_2043995
Rabbit polyclonal anti-cleaved caspase-3	Cell Signaling Technology	Cat# 9661S; RRID:AB_2341188
Rat monoclonal anti-CD45 Antibody (YAML 501.4)	Santa Cruz Biotechnology	Cat# sc-65344; RRID:AB_782214
CD14	BD Biosciences	Available from Translational and Correlative Studies Laboratory upon request
CD19	BD Biosciences	Available from Translational and Correlative Studies Laboratory upon request
CD3	BD Biosciences	Available from Translational and Correlative Studies Laboratory upon request
CD45RA	BD Biosciences	Available from Translational and Correlative Studies Laboratory upon request
CD27	Biolegend	Available from Translational and Correlative Studies Laboratory upon request
CCR7	BD Biosciences	Available from Translational and Correlative Studies Laboratory upon request
Monoclonal Mouse Anti-Human CD3(LN10)	Leica Biosystems	Cat# PA0553; RRID: AB_10554601
FLEX Monoclonal Mouse Anti-Human CD68 (KP1)	Dako	Cat# IR609
Alexa Flour 647 Donkey anti-Rabbit IgG (H+L) Highly Cross-Adsorbed Secondary Antibody	Thermo Fisher Scientific	Cat# A-31573; RRID: AB_2536183
Alexa Flour 488 anti-Mouse IgG (H+L) Highly Cross-Adsorbed Secondary Antibody	Thermo Fisher Scientific	Cat# A-21202; RRID: AB_141607
Alexa Flour 594 Donkey anti-Rat IgG (H+L) Highly Cross-Adsorbed Secondary Antibody,	Thermo Fisher Scientific	Cat#A-21209; RRID: AB_2535795
Alexa Flour 532 Mouse Monoclonal anti-human CD45 (HI30)	Thermo Fisher Scientific	Cat# 58-0459-41; RRID: AB_11218084
eFluor 450 Mouse anti- human CD69(FN50)	Fisher Scientific	Cat# 15568573
Alexa Fluor 647 Rabbit Monoclonal anti- TCF1 (C63D9)	Cell Signaling Technology	Cat# 6709S; RRID: AB_2797631
Alexa Fluor 660 Mouse anti-human CD223 (LAAG-3) (3DS223H)	Thermo Fisher Scientific	Cat# 606-2239-42; RRID: AB_2896269
Alexa Fluor 700 Mouse anti- human Ki67(B56)	BD Biosciences	Cat# 561277; RRID: AB_10611571
APC Fire 810 Mouse anti- human CD27 (QA17A18)	Biolegend	Cat# 393213; RRID: AB_2860961
APC efluor 780 Mouse monoclonal anti- CD137 (4-1BB) (4B4-1)	FischerScientific	Cat# 17123913; RRID: AB_2848365
PerCP Efluor710 Mouse monoclonal anti-TIGIT (MBSA43)	Thermo Fisher Scientific	Cat# 46-9500-42; RRID: AB_10853679
BUV395 Mouse anti-human CD2 (RPA-2.10)	BD Biosciences	Cat# 563820;RRID: AB_2744356
BUV496 Mouse anti-human CD38 (HIT2)	BD Biosciences	Cat# 612946;RRID: AB_2870225
BUV563 Mouse anti-human CD8 (SK1)	BD Biosciences	Cat# 741440; RRID: AB_287096
BUV615 Mouse anti-human CD25 (2A3)	BD Biosciences	Cat# 612996; RRID: AB_2870268
BUV661 Mouse anti-human CD226 (DX11)	BD Biosciences	Cat# 741934; RRID: AB_2874171
BUV737 Mouse anti-human CD279 (PD-1) (EH12.1)	BD Biosciences	Cat# 612792; RRID: AB_2870119
SuperBright600 Mouse monoclonal anti-human CD3 (OKT3)	Thermo Fisher Scientific	Cat# 63-0037-42; RRID: AB_2637435
PE-eFluor 610 Mouse anti-human EOMES (WD1928)	Thermo Fisher Scientific	Cat# 61-4877-42; RRID: AB_2574616
Brilliant Volet 480 Mouse anti-human CD127 (HIL-7R-M21 (RUO))	BD Biosciences	Cat# 566158; RRID: AB_2869742
PE-Cy7 Mouse monoclonal anti-human CD45RA (HI100)	Ebioscience	Cat# 25-0458-42; RRID: AB_1548774
Brilliant Violet 650 Mouse monoclonal anti human Tbet (O4-46)	BD Biosciences	Cat# 564142; RRID: AB_2738616
Brilliant Violet 711 Mouse monoclonal anti human TIM3 (CD366) (7D3)	BD Biosciences	Cat# 565567; RRID: AB_2744370
BV750 Mouse anti human CD39 (TU66)	BD Biosciences	Cat# 747079; RRID: AB_2871834
PE Recombinant anti human TOX (REA473)	Miltenyi	Cat# 130-120-716; RRID: AB_2801780
PE-Cy5 Mouse anti human CTLA4 (BN13)	BD Biosciences	Cat# 555854; RRID: AB_396177
BB700 Mouse anti human FOXP3 (236A/E7)	BD Biosciences	Cat# 566526; RRID: AB_2744476
Qdot800 Mouse anti human CD4 (S3.5)	ThermoFisher Scientific	Cat# Q22153; RRID: AB_2556509
Spark Violet 538 Mouse anti human HLA-DR (L243)	BioLegend	Cat# 307678; RRID: AB_2890742
Brilliant Violet 421 Streptavidin conjugate	BioLegend	Cat# 405226
Biological samples		
Patient CSF Samples	This Paper	N/A
Patient Tumor Tissue	This Paper	N/A
Patient FFPE Tumor Tissue	This Paper	N/A
Patient-derived GBO (UP-O-9121)	Sun et al.^73^	PMID: 39821165
Infusion product	This paper	N/A
Chemicals, peptides, and recombinant proteins		
Recombinant human IL-2	STEMCELL Technologies	Cat# 78036
Recombinant human insulin	Sigma	Cat# 19278
ImmunoCult^™^-XF T Cell Expansion Medium	STEMCELL Technologies	Cat# 10981
RPMI-1640 Media	Thermo Fisher	Cat# 11875093
Calcein AM	Thermo Fisher	Cat# C1430
TO-PRO-3 Iodide	Thermo Fisher	Cat# T3605
DMEM/F-12	Millipore Sigma	Cat# D8437
Fixable Viability Stain	BD Biosciences	Cat# 564406
B-27 Supplement	Thermo Fisher	Cat# 17504044
GlutaMax Supplement	Thermo Fisher	Cat# 35050061
DAPI	Thermo Fisher	Cat# D1306
Hibernate A	Thermo Fisher	Cat# A1247501
Neurobasal Medium	Thermo Fisher	Cat# 21103049
N2 Supplement	Thermo Fisher	Cat# 17502048
Antibiotic-Antimycotic	Thermo Fisher	Cat# 15240062
Critical commercial assays		
Chromium GEM-X Single Cell 5’ Reagent Kit v3	10x Genomics	Cat# PN-1000699
Chromium GEM-X Single Cell 5’ Chip Kit	10x Genomics	Cat# PN-1000698
Chromium Single Cell Human TCR Amplification	10x Genomics	Cat# PN-1000252
Dual Index Kit TT Set A	10x Genomics	Cat# PN-1000215
EasySep RBC depletion reagent	STEMCELL Technologies	Cat# 18170
MACSPlex Cytokine 12 Kit, human	Miltenyi Biotec	Cat# 130-099-169
KAPA quantitative PCR kit	Roche	Cat# KK4824
High-Sensitivity D5000 Screentape	Agilent Technologies	Cat# 5067-5592
High-Sensitivity RNA Screentape	Agilent Technologies	Cat# 5067-5579
Fixation/Permeabilization solution	Thermo Fisher	Cat# 00-5523-00
High Sensitivity D5000 sample buffer and ladder	Agilent	Part# 5067-5593
Human sCD27 Instant ELISA Kit	Invitrogen	Cat# BMS286INST
RNAscope^™^ 2.5 LS Probe- Hs-EGFR	ACD	Cat# 310068
Chromogenic detection kit	ACD	Cat# 322150
BOND RED Detection System	Leica	Cat# DS9390
Bond Prime Polymer Refine Detection System	Leica	Cat# DS9284
Bond Prime Epitope Retrieval 1	Leica	Cat# AR0086
Bond Prime Epitope Retrieval 2	Leica	Cat# AR0087
RNeasy FFPE Kit	Qiagen	Cat# 73504
SMART-Seq^®^ Total RNA Pico Input with UMIs (ZapR^™^ Mammalian)	Takara	Cat# 634354
Unique Dual Index Kit (1-24)	Takara	Cat# 634756
Deposited data		
Single-cell RNA-seq data	This Paper	https://www.ncbi.nlm.nih.gov/geo/query/acc.cgi?acc=GSE296419
Processed Single-cell data (Viz)	This Paper	https://singlecell.broadinstitute.org/single_cell/study/SCP3237/the-critical-role-of-the-host-endogenous-immune-compartment-after-intracerebroventricular-car-t-cell-therapy-in-recurrent-gbm
Patient clinical data	Bagley et al.^14^	https://doi.org/10.1038/s41591-025-03824-2
Glioblastoma organoid gene expression data (Figure 3K)	Zhang et al.^51^	https://doi.org/10.1101/2024.10.03.616537
Software and algorithms		
Cell Ranger v8.0.1	10x Genomics	https://support.10xgenomics.com/
Seurat v5.2.1	Satija Lab	https://satijalab.org/seurat/
Scanpy v1.9.1	Theis Lab	https://scanpy.readthedocs.io/
CellBender v0.3.0	Fleming et al.^74^	https://github.com/broadinstitute/CellBender
Scrublet v0.2.3	Wolock et al.	https://github.com/swolock/scrublet
Harmony v1.2.0	Korsunsky et al.^75^	https://github.com/immunogenomics/harmony
CellTypist v1.6.3	Domínguez Conde et al.^76^	https://www.celltypist.org/
inferCNV v1.13.0	Broad Institute	https://github.com/broadinstitute/infercnv
scCODA v0.1.9	Büttner et al.^77^	https://github.com/theislab/scCODA
muscat v1.16.0	Crowell et al.^78^	https://github.com/HelenaLC/muscat
fgsea v1.28.0	Korotkevich et al.^79^	https://bioconductor.org/packages/fgsea/
UCell v2.6.2	Andreatta & Carmona^80^	https://github.com/carmonalab/UCell
scRepertoire v2.3.4	Borcherding et al.^81^	https://github.com/ncborcherding/scRepertoire
LIANA v0.1.14	Dimitrov et al.^82^	https://github.com/saezlab/liana
FlowJo v10.10.0	Becton, Dickinson	https://www.flowjo.com/
OMIQ	OMIQ, Inc.	https://www.omiq.ai/
ImageJ Fiji v2.9.0	Schindelin et al.^83^	https://imagej.net/software/fiji/
Zen 2 software	Zeiss	https://www.zeiss.com/microscopy/us/products/software/zeiss-zen.html
MACSQuantify software	Miltenyi Biotec	https://www.miltenyibiotec.com/US-en/products/macs-flow-cytometry/software/macsquantify/flow-cytometry-software.html
Python v3.10.12	Python Software Foundation	https://www.python.org
scanpy v1.10.3	Theis Lab	https://scanpy.readthedocs.io/en/stable/
pandas v2.0.3	NumFOCUS, Inc.	https://pandas.pydata.org/
numpy v1.23.5	NumFOCUS, Inc.	https://numpy.org/
scipy v1.11.2	Virtanen et al.^84^	https://scipy.org/
sklearn v1.3.0	Pedregosa et al.^85^	https://scikit-learn.org/stable/
h5py v3.11.0	Andrew Collette	https://www.h5py.org/
anndata v0.10.9	Virshup et al.^86^	https://anndata.readthedocs.io/en/stable/
cnmf_parallel v7	Kotliar et al.^87^	https://app.terra.bio/#workflows/mparikh/cnmf_parallel/7
simctest v2.6.1	Gandy et al.^88^	https://cran.r-project.org/web/packages/simctest/index.html
gprofiler2 v0.2.3	Kolberg et al.^89^	https://cran.r-project.org/web/packages/gprofiler2/index.html
decoupleR v2.8.0	Saez Lab	https://saezlab.github.io/decoupleR/
DoRothEA v1.20.0	Garcia-Alonso et al.^90^	https://www.bioconductor.org/packages/release/data/experiment/html/dorothea.html
viper v1.42.0	Alvarez et al.^91^	https://www.bioconductor.org/packages/release/bioc/html/viper.html
limma v3.66.0	Ritchie et al.^92^	https://bioconductor.org/packages/release/bioc/html/limma.html
edgeR v4.0.16	Chen et al.^93^	https://bioconductor.org/packages/release/bioc/html/edgeR.html
clusterProfiler v4.10.1	Yu et al.^94^	https://github.com/YuLab-SMU/clusterProfiler
STAR v 2.7.9a	Dobin et al.^95^	https://github.com/alexdobin/STAR
umi_tools v1.1.6	Smith et al.^96^	https://github.com/CGATOxford/UMI-tools
Cutadapt v5.2	Algorithmic Bioinformatics Group, Saarland University	https://github.com/marcelm/cutadapt/
featureCounts v2.1.1	Subread	https://subread.sourceforge.net/
biomaRt v2.58.2	Ensembl	https://grch37.ensembl.org/info/data/biomart/index.html
Other		
SH800S cell sorter system	Sony Biotechnology	N/A
NovaSeq 6000 sequencer	Illumina	CHOP High Throughput Sequencing Core
Chromium X controller	10x Genomics	PN#1000818
NextSeq 2000 sequencer	Illumina	Penn Genomics and Sequencing Core
4150 TapeStation System	Agilent	Part# G2992AA
GentleMACS Octo Dissociator	Miltenyi Biotec	Cat# 130-093-235
MACSQuant Analyzer 10	Miltenyi Biotec	Cat# 130-096-343
Zeiss LSM 710 microscope	Zeiss	N/A
LMD7000 microscope	Leica	N/A
PEN Membrane Slides	Leica	Cat# 11505158
Synergy H4 Hybrid Reader	BioTek	N/A
Bond-PRIME instrument	Leica	Cat# 91.0021

## Highlights

- Intracerebroventricular CAR T cells show increased cytotoxicity and exhaustion in CSF
- Endogenous NK, complement myeloid, and Tregs undergo dose-dependent changes post infusion
- Favorable outcomes correlate with expansion of CD56^dim^CD16^pos^ cytotoxic NK cells
- Treg expansion and baseline scavenger myeloid cells correlate with poor outcomes
