# Supplementary material for: The critical role of the endogenous immune compartment after CAR T cell therapy in recurrent GBM
Source: Cell. Author manuscript; Available in PMC 2026 Jul 27. (PMC13401735; doi:10.1016/j.cell.2026.05.026)
Supplement: Supplementary file 1 [file NIHMS2188461-supplement-1.pdf]

Data S1. Additional data on single-cell sequencing batch effects, flow cytometry gating, infusion product characteristics, CAR target expression, tumor-infiltrating immune cells, steroid management, and ligand-receptor analysis, related to **Figures 1-4, 6**.

1. Single cell batch and patient effects

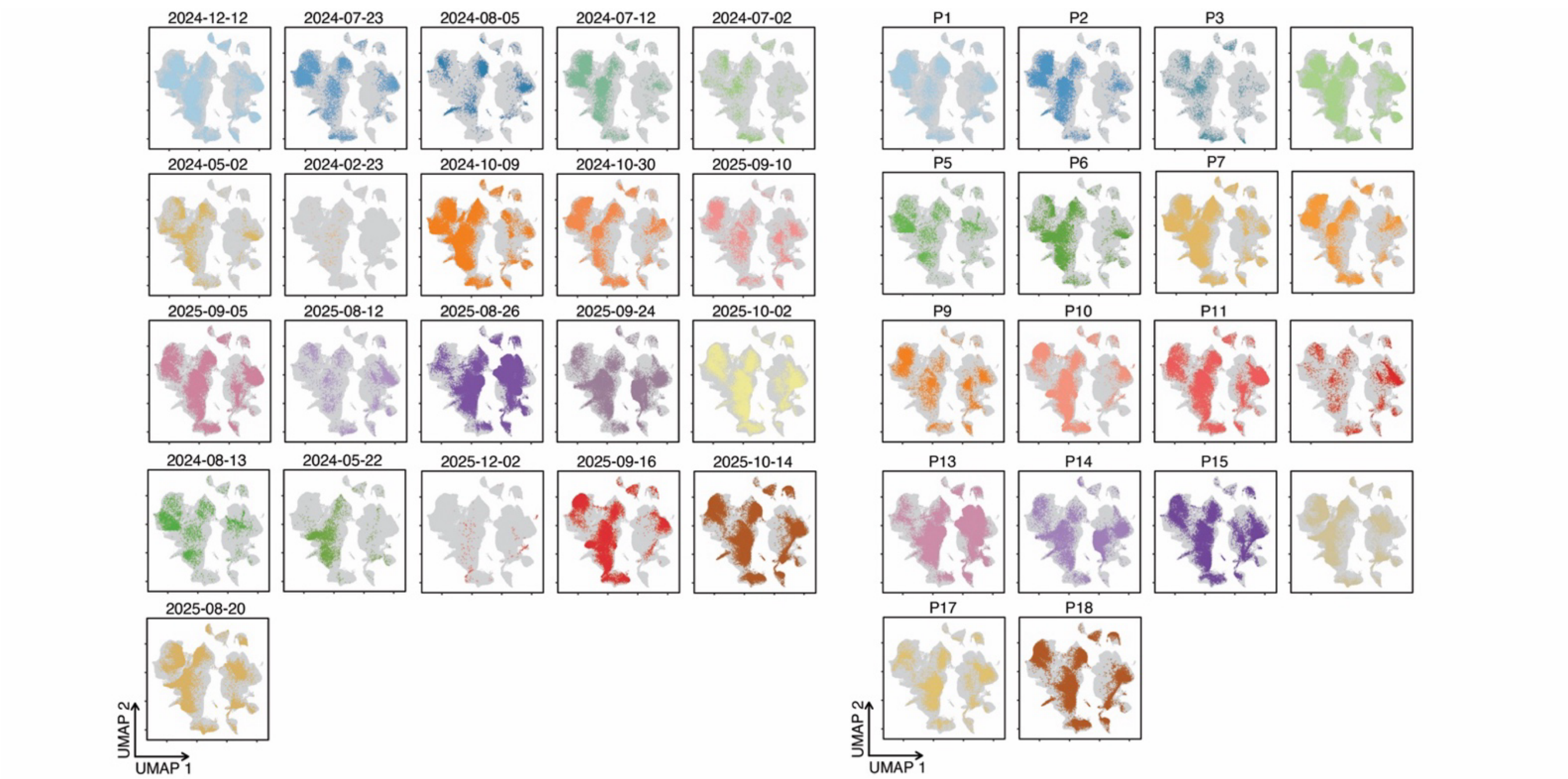

CSF batch and patient effects. UMAP embedding of CSF cells (n = 248,834 cells; 44 samples across 18 patients), corresponding to the global immune landscape shown in Main Figure 1C. Cells are colored by sequencing batch (left) and by individual patient identifier (right).

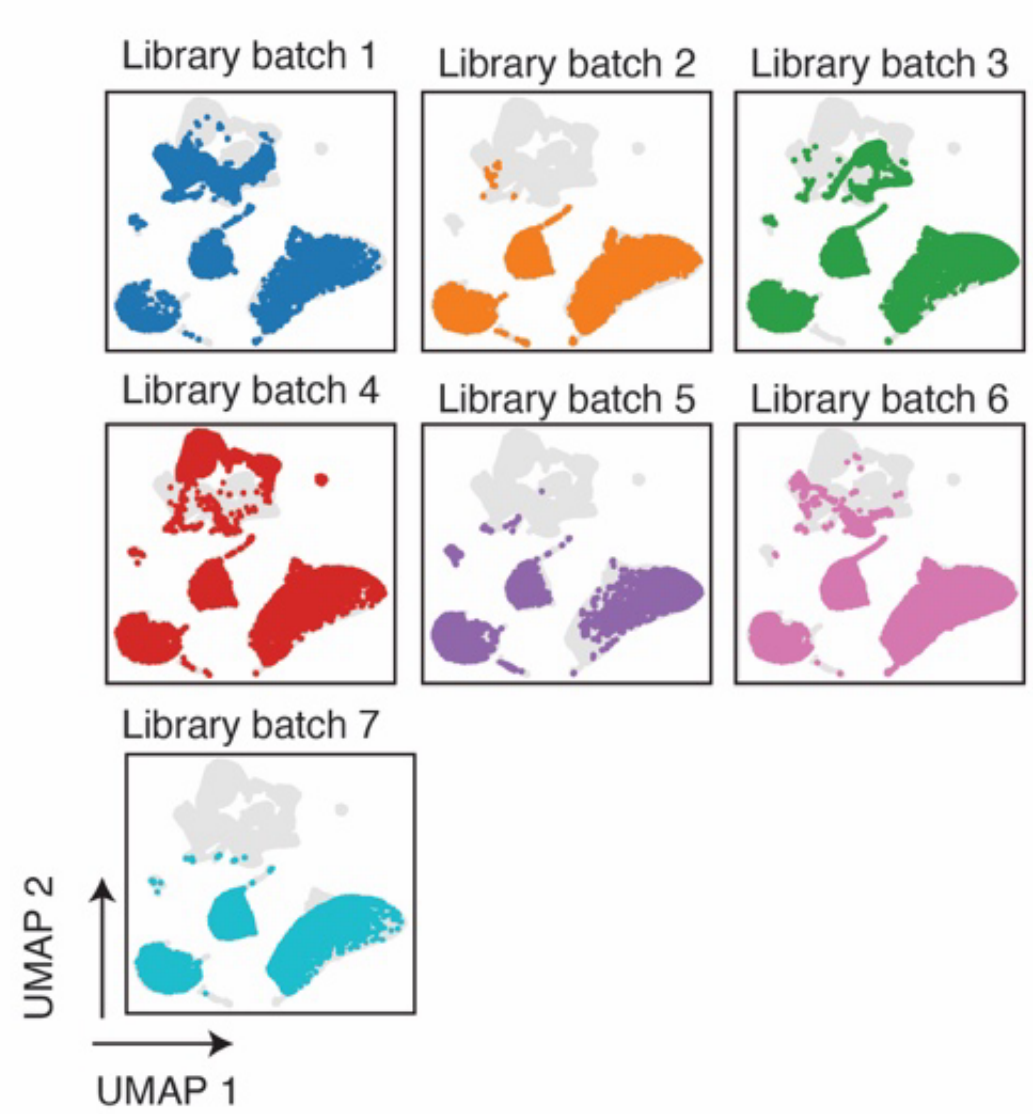

Tumor batch and patient effects. UMAP embedding of tumor tissue cells (*n* = 98,809, 10 samples across 6 patients), corresponding to the global immune landscape shown in Main Figures 3B and C. Cells are colored by sequencing batch.

2. Infusion Product

2.1. Spectral Cytometry: Gating Strategy.

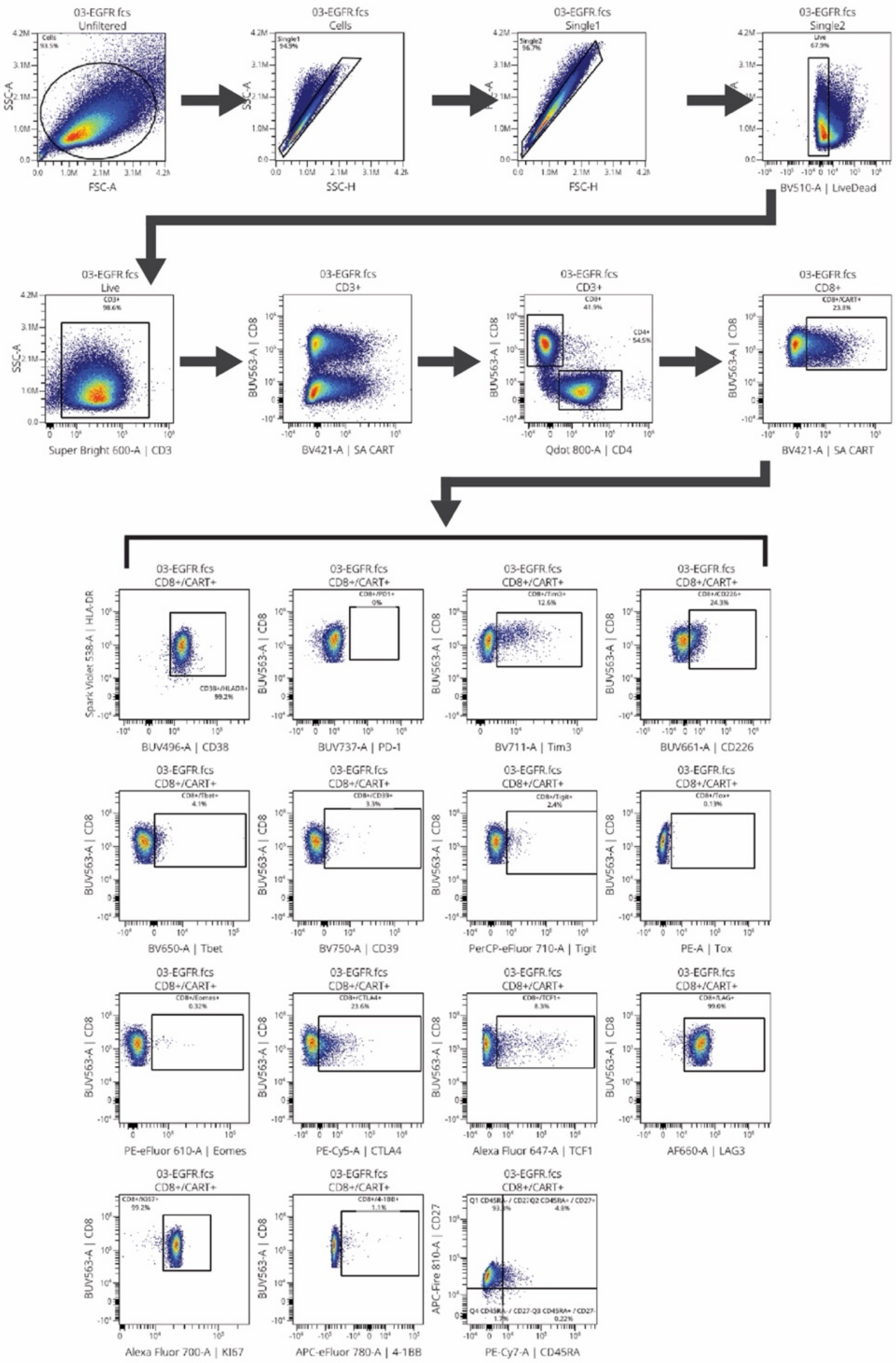

Cells are gated in a FSCA/SSCA scatterplot, then Singlets are identified using SSC-H/SSC-A and FSC-H/FSC-A. Live Cells are identified as LiveDead negative. T cells are gated on Live cells using CD3. CD4 and CD8 T cells are identified in CD3 positive cells. CD8 CART cells are further identified, and every additional marker is evaluated in this gate (CD38/HLA-DR, PD-1, Tim3, CD226, Tbet, CD39, Tigit, Tox, Eomes, CTLA-4, TCF1, LAG-3, Ki67, 41BB, CD45RA/CD27).

2.2. Infusion product characteris tics for responders vs. non-responders

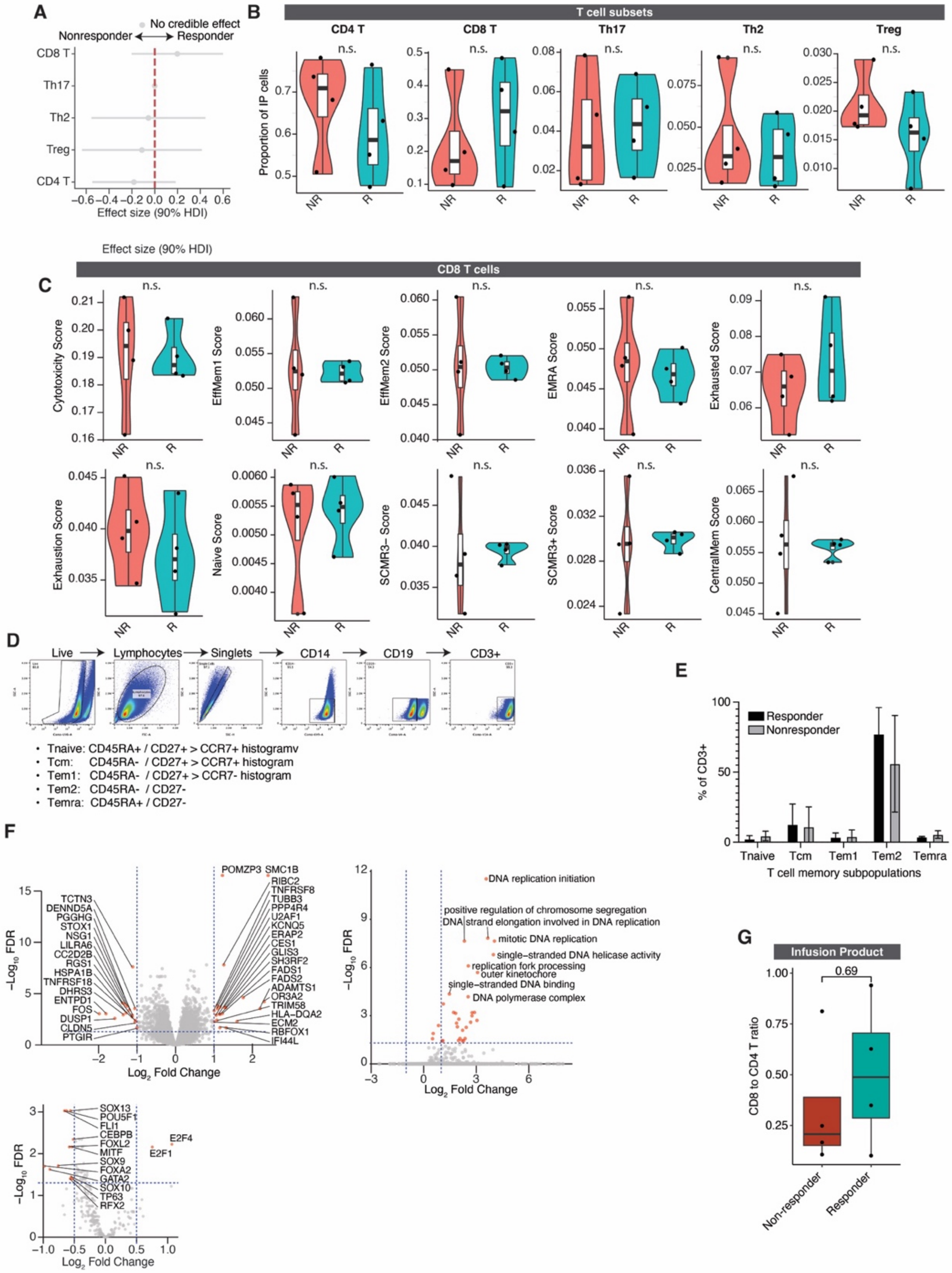

(A) Bayesian analysis of IP subset composition (Responders vs. Non-responders; *n* = 79,563 cells from 4 responders and 4 non-responders). Points represent the mean effect size and error bars represent the 90% Highest Density Interval (HDI). Grey indicates no credible effect (90% HDI overlaps 0).
(B) Proportion of each cell type in the IP stratified by response status. Violin plots show the frequency of cell types (CD4 T, CD8 T, Th17, Th2, Treg). Points represent individual patient samples. Box plots indicate median and interquartile range. Statistical significance was determined using the Wilcoxon rank-sum test.
(C) Differentiation state for CAR CD8 T cells in the IP stratified by response status, calculated using UCell scores. Statistical significance was determined using the Wilcoxon rank-sum test on pseudobulked patient-level data.
(D) Gating strategy for the infusion product. Representative gating strategy showing the hierarchy: Live > Lymphocytes > Singlets > CD14-/CD19- > CD3+. T cell memory subpopulations (Tnaive, Tcm, Tem1, Tem2, Temra) were defined based on CD45RA, CD27, CCR7, and CD95 expression.
(E) Differentiation states by flow for the infusion product. Bar graphs showing the percentage of CD3+ cells in each memory subpopulation. Data are presented as [mean ± SD]. Statistical significance was determined using the [Wilcoxon rank-sum test].
(F) Characteristics of the IP: differentially expressed genes, transcription factors, and pathway enrichment analysis. (Left, top) Volcano plot of differentially expressed genes analyzed with edgeR (pseudobulk to patient level, controlling for cell type). (Left, bottom) Volcano plot of transcription factor activity inferred using DoRothEA and VIPER, with differential analysis by limma. (Right) Volcano plot of pathway enrichment analysis performed with gprofiler2. Dashed lines indicate significance thresholds.
(G) Ratio of CAR CD8:CD4 T cells in the IP stratified by response status.

2.2. Infusion product characteristics for responders vs. non-responders (continued).

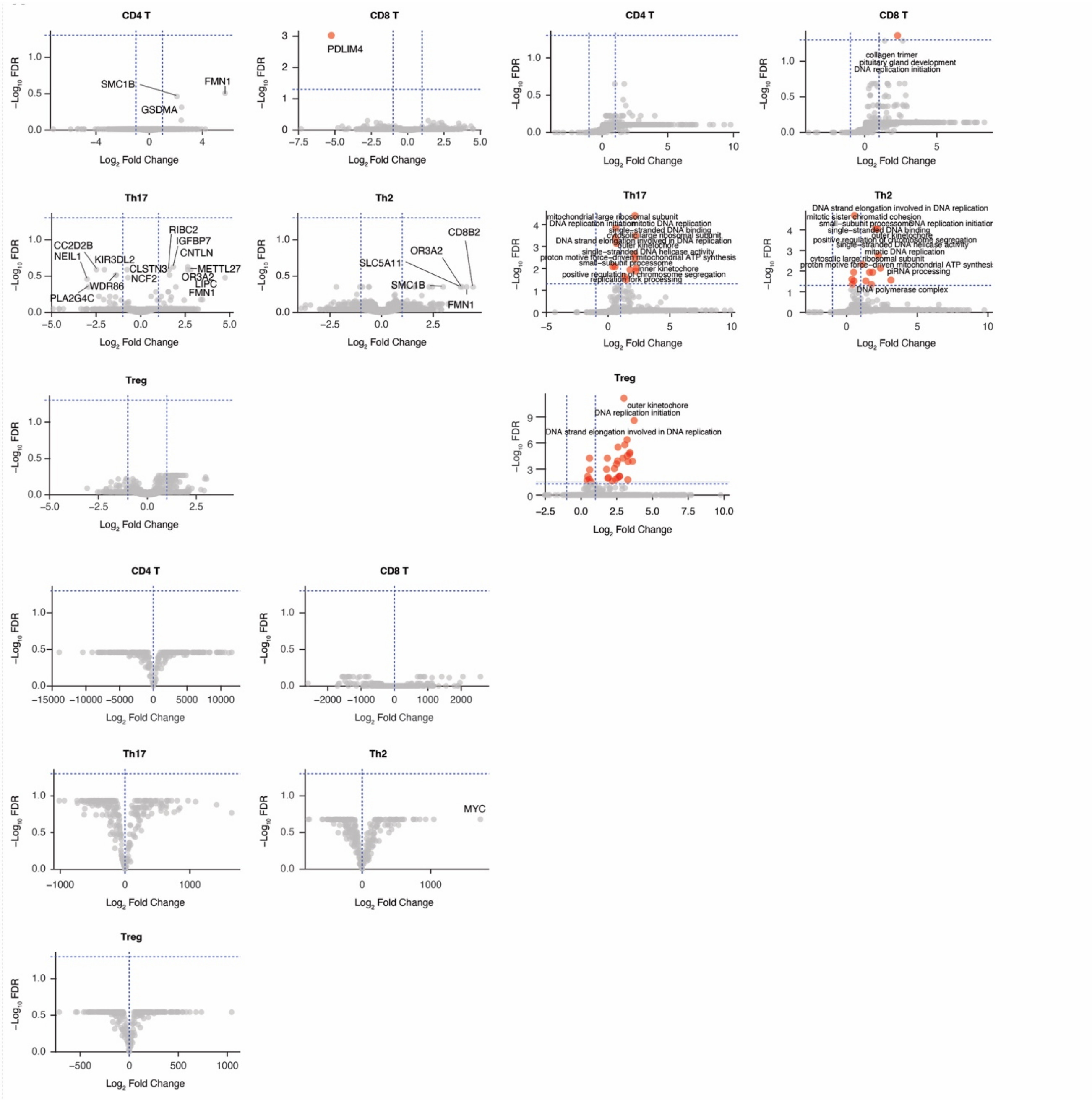

Significant differentially expressed genes, pathways, and transcription factors per cell type. Grid of volcano plots stratified by T cell subset (CD4 T, CD8 T, Th17, Th2, Treg). Top rows: Differentially expressed genes (edgeR). Middle rows: Enriched pathways (gprofiler2). Bottom rows: Differential transcription factor activity (DoRothEA/VIPER/limma). The x-axis represents Log2 Fold Change (Responder vs. Non-responder) and the y-axis represents -Log10 FDR

2.3. Infusion product characteristics of clones with matched TCR to the tumor tissue

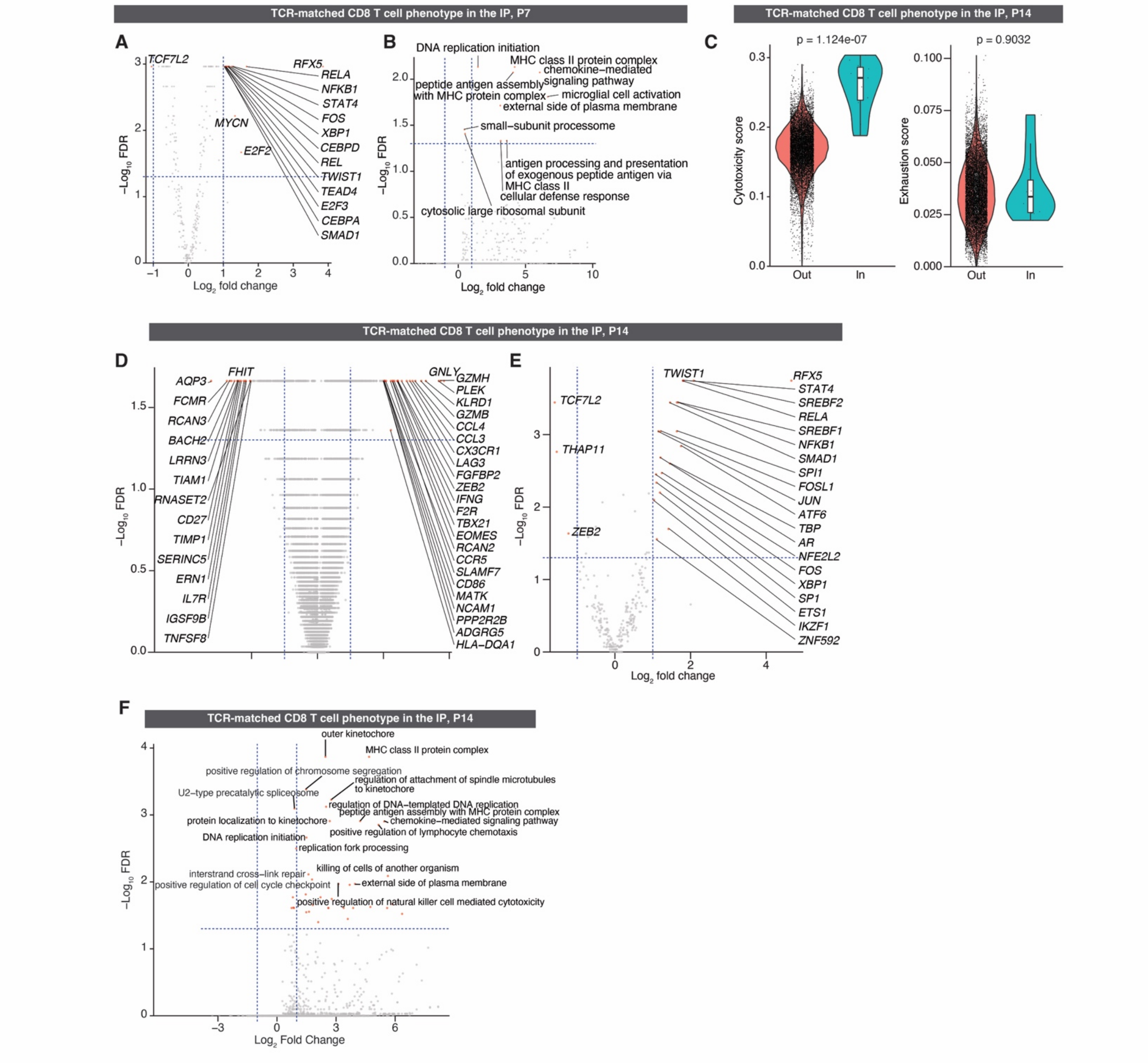

(A) Differential analysis of transcription factor activity inferred by VIPER in single cell RNA-seq data from CD8 T cells in the P7 infusion product. Transcription factor-target interactions sourced from DoRothEA (conf. levels A, B, and C). Differential analysis performed by Monte Carlo hypothesis testing with MMCtest. See methods (*Differential TF activity*) for additional details. Dashed lines indicate significance threshold. Positive fold change indicates enrichment in TCR-matched observed cells (*n* = 67 TCR-matched observed, 2,523 Non-matched).

(B) Differentially enriched gene sets in single cell RNA-seq data from CD8 T cells in patient 7 infusion product. Pathway enrichment assessed using gprofiler2. Dotted lines represent a significance threshold. Positive fold change indicates enrichment in TCR-matched observed cells (*n* = 67 TCR-matched observed, 2,523 Non-matched).

(C) Cytotoxicity (Pace et al.) and Exhaustion (Yost et al.) score of TCR-matched observed (*n* = 11) vs. Non-matched (*n* = 12,823) CD8 T cells in single-cell RNA-seq data from the P14 infusion product. P value from Wilcoxon test.

(D) Differential gene expression analysis of single-cell RNA-seq data from the patient 14 infusion product. Differential analysis performed by Monte Carlo hypothesis testing with MMCtest. Positive fold change indicates enrichment in TCR-matched observed cells (*n* = 11 TCR-matched observed, 12,823 Non-matched). Dashed lines indicate a significance threshold. See methods (Infusion Product and Infiltration Analysis) for further details.

(E) Differential analysis of transcription factor activity inferred by VIPER in single cell RNA-seq data from CD8 T cells in P14 infusion product. Transcription factor-target interactions sourced from DoRothEA (conf. levels A, B, and C). Differential analysis performed by Monte Carlo hypothesis testing with MMCtest. See methods (*Differential TF activity*) for additional details. Dashed lines indicate a significance threshold. Positive fold change indicates enrichment in TCR-matched observed cells (*n* = 11 TCR-matched observed, 12,823 Non-matched).

(F) Differentially enriched gene sets from the P14 infusion project. Gene set enrichment assessed using gprofiler2 Positive fold change indicates an enrichment in TCR-matched observed cells (*n* = 11 TCR-matched observed, 12,823 Non-matched). Dashed lines indicate a significance threshold. See methods (*Infusion Product and Infiltration Analysis*) for further details.

3. Tumor tissue

3.1. Patient tumor sample H&E and Immunohistochemistry staining

Histologic (H&E) and immunohistochemical evaluation of matched pre- and post-treatment tumor tissues to assess the spectrum of post-infusion changes, immune cell infiltration, and target expression.

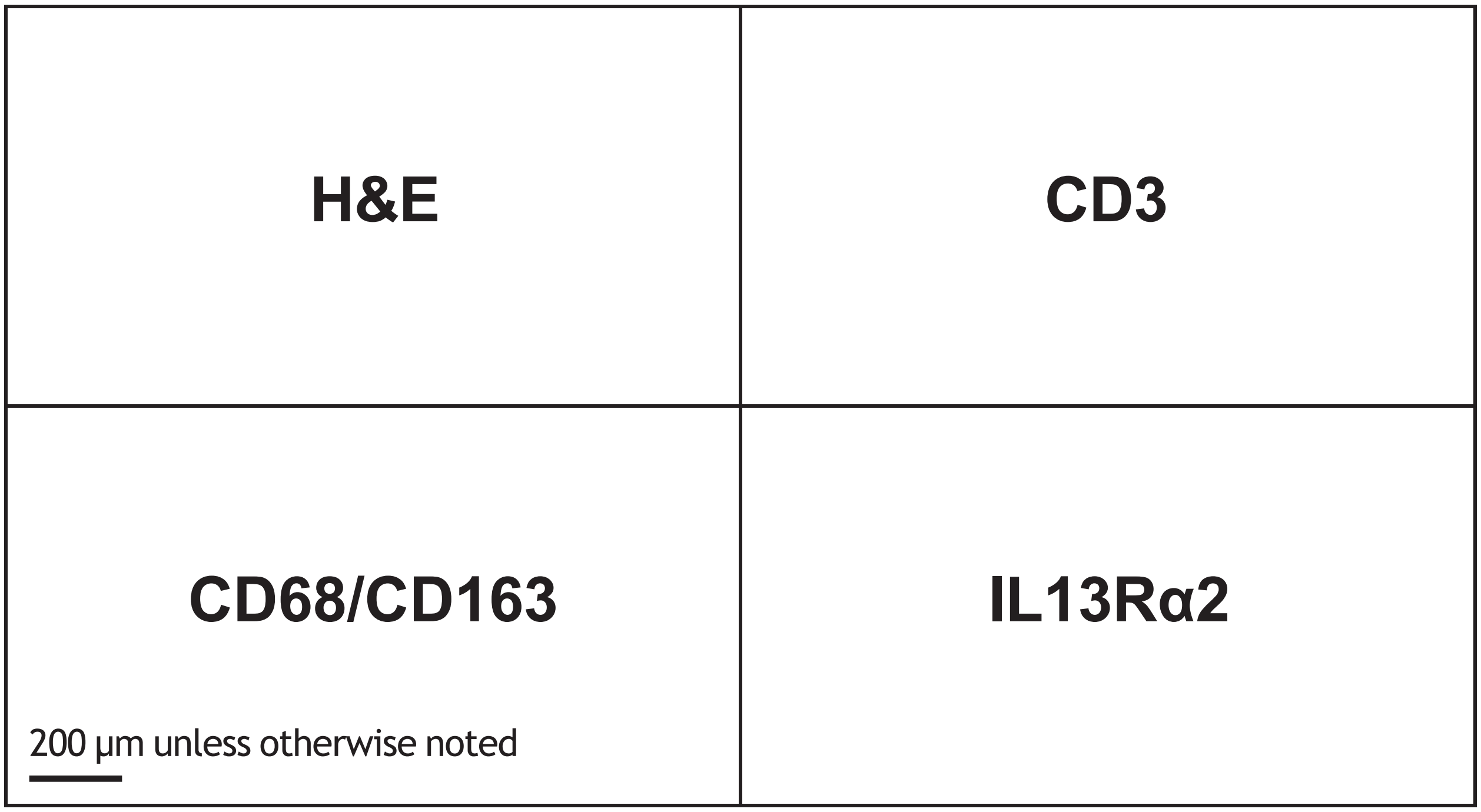

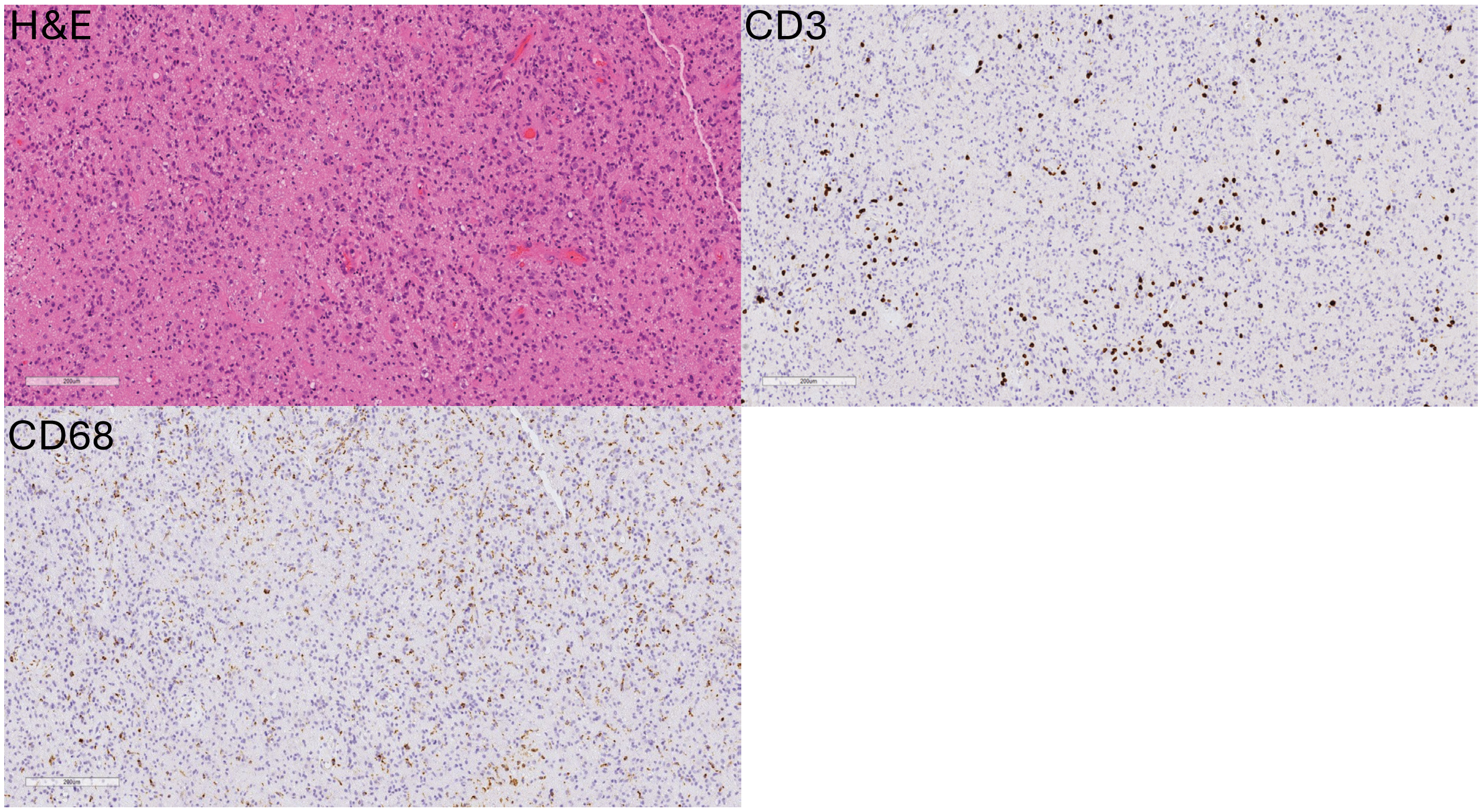

16321-04, P1, Pre-infusion

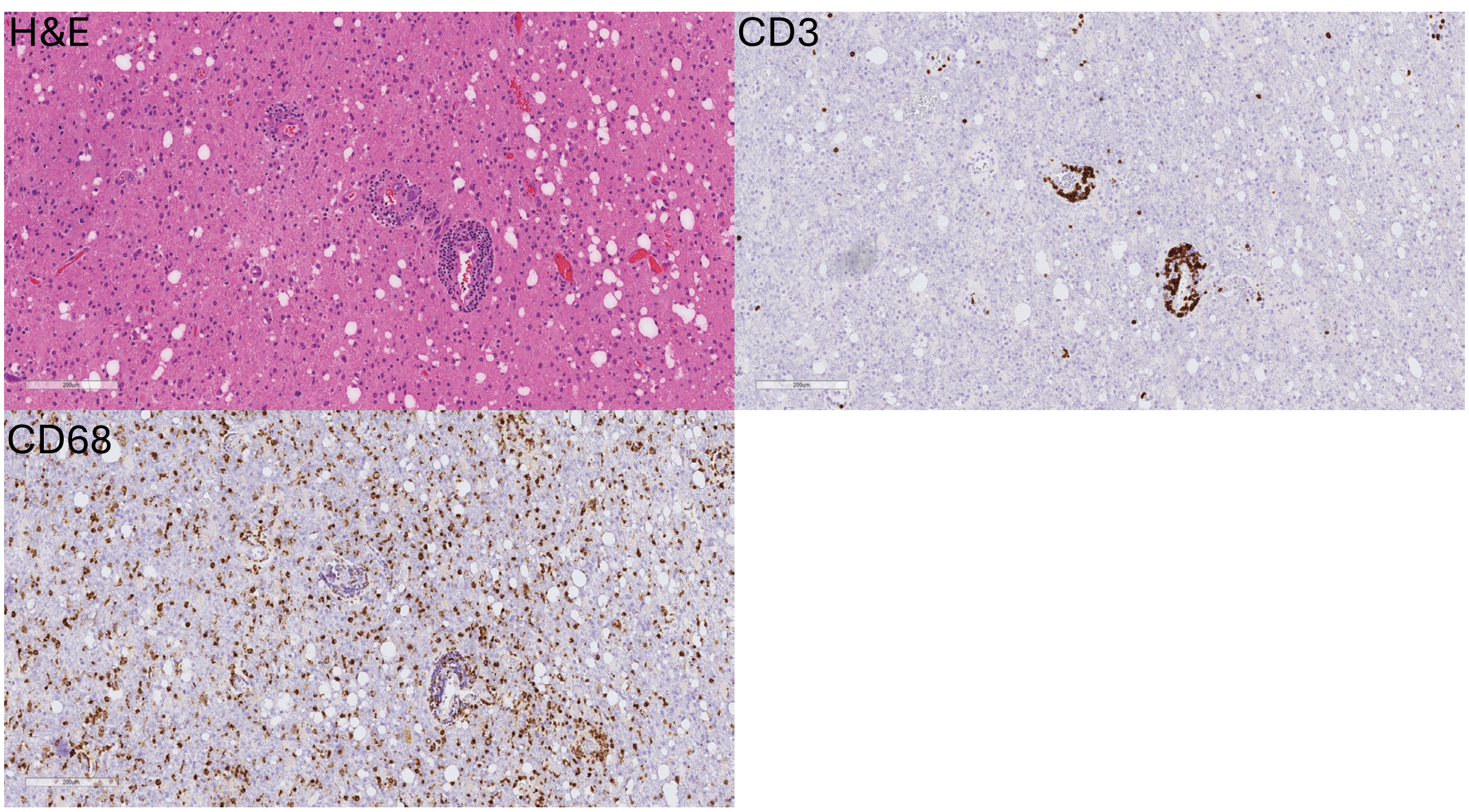

16321-04, P1, Post-infusion

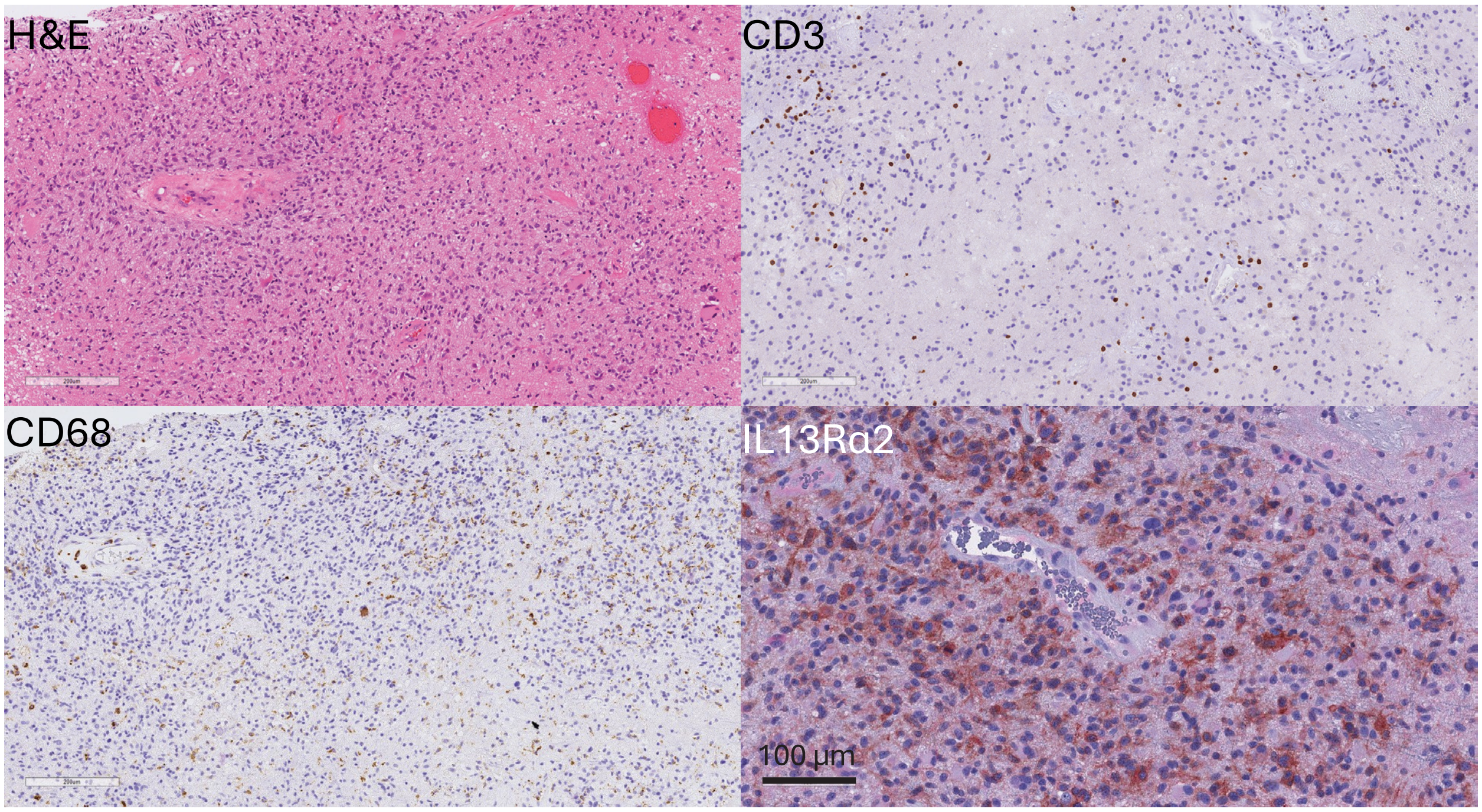

16321-10, P4, Pre-infusion

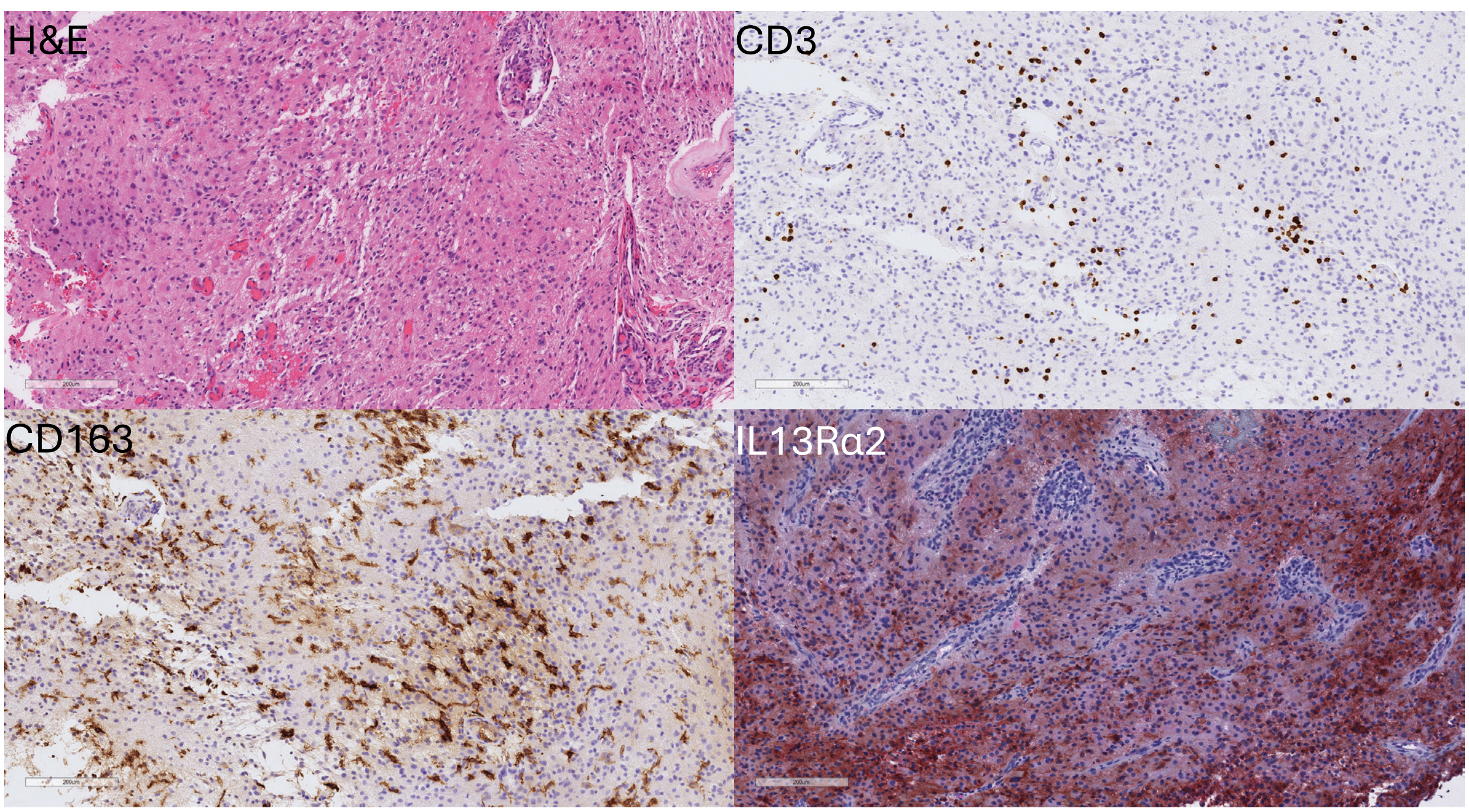

16321-10, P4, Post-infusion

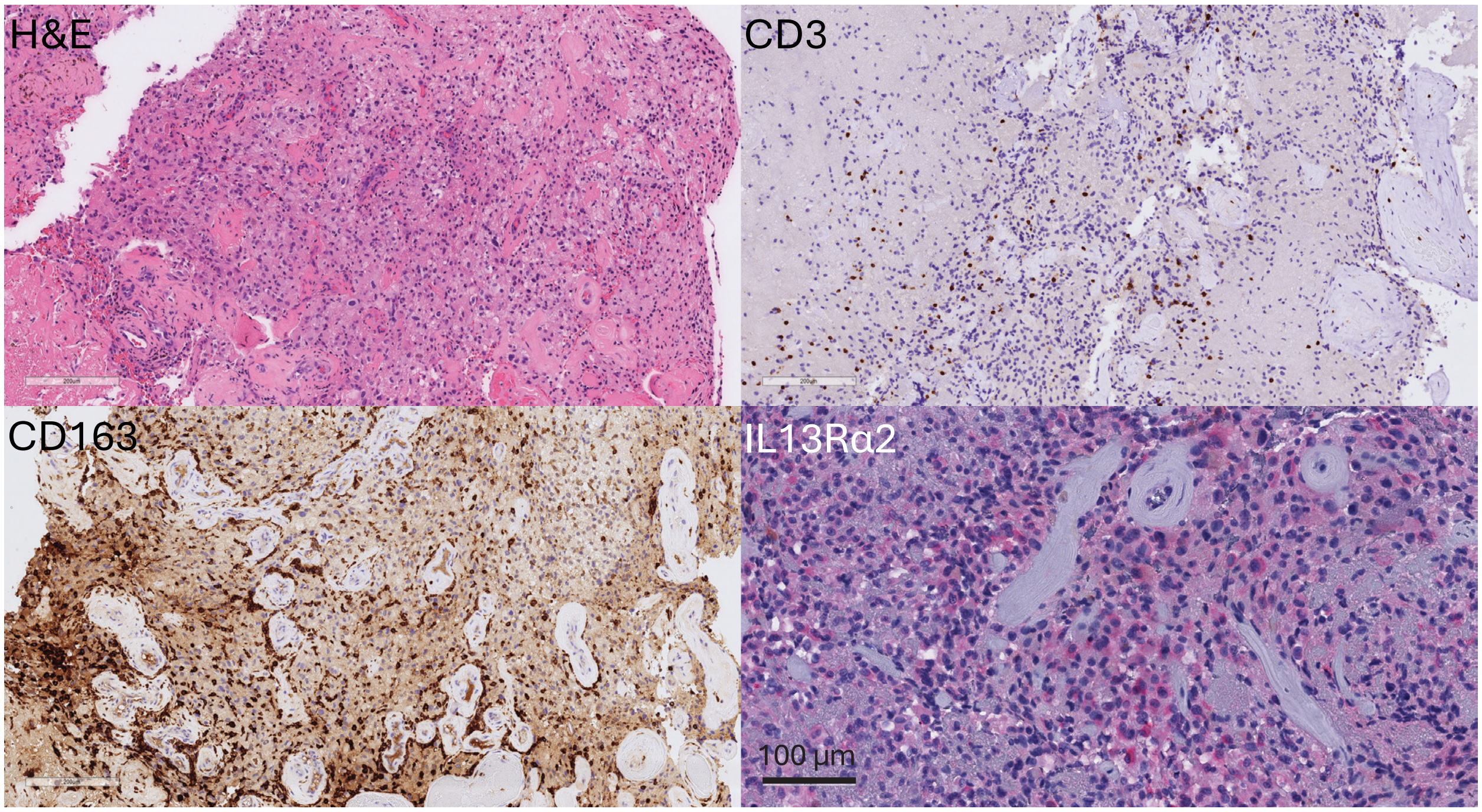

16321-28, P7, Pre-infusion

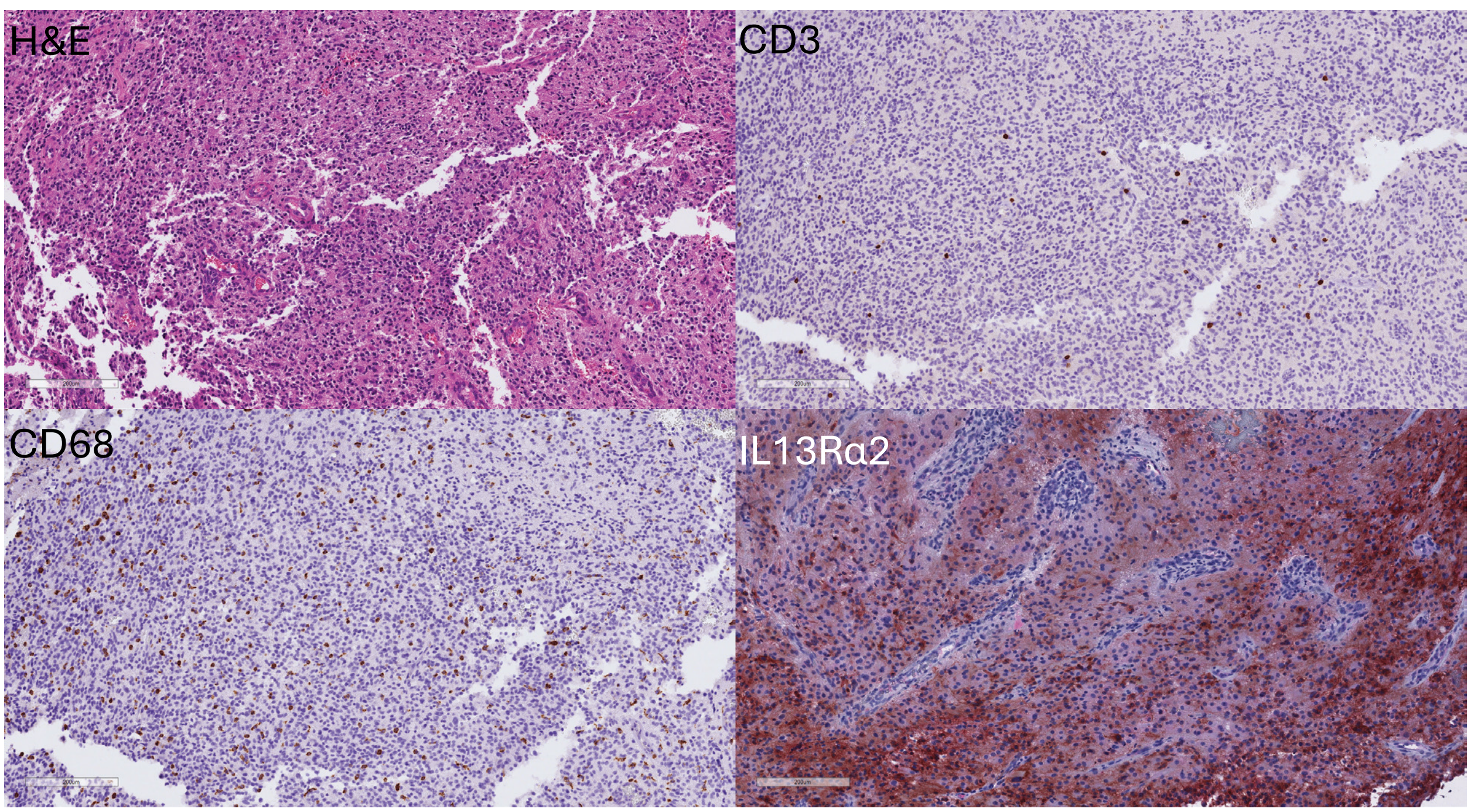

16321-28, P7, Post-infusion

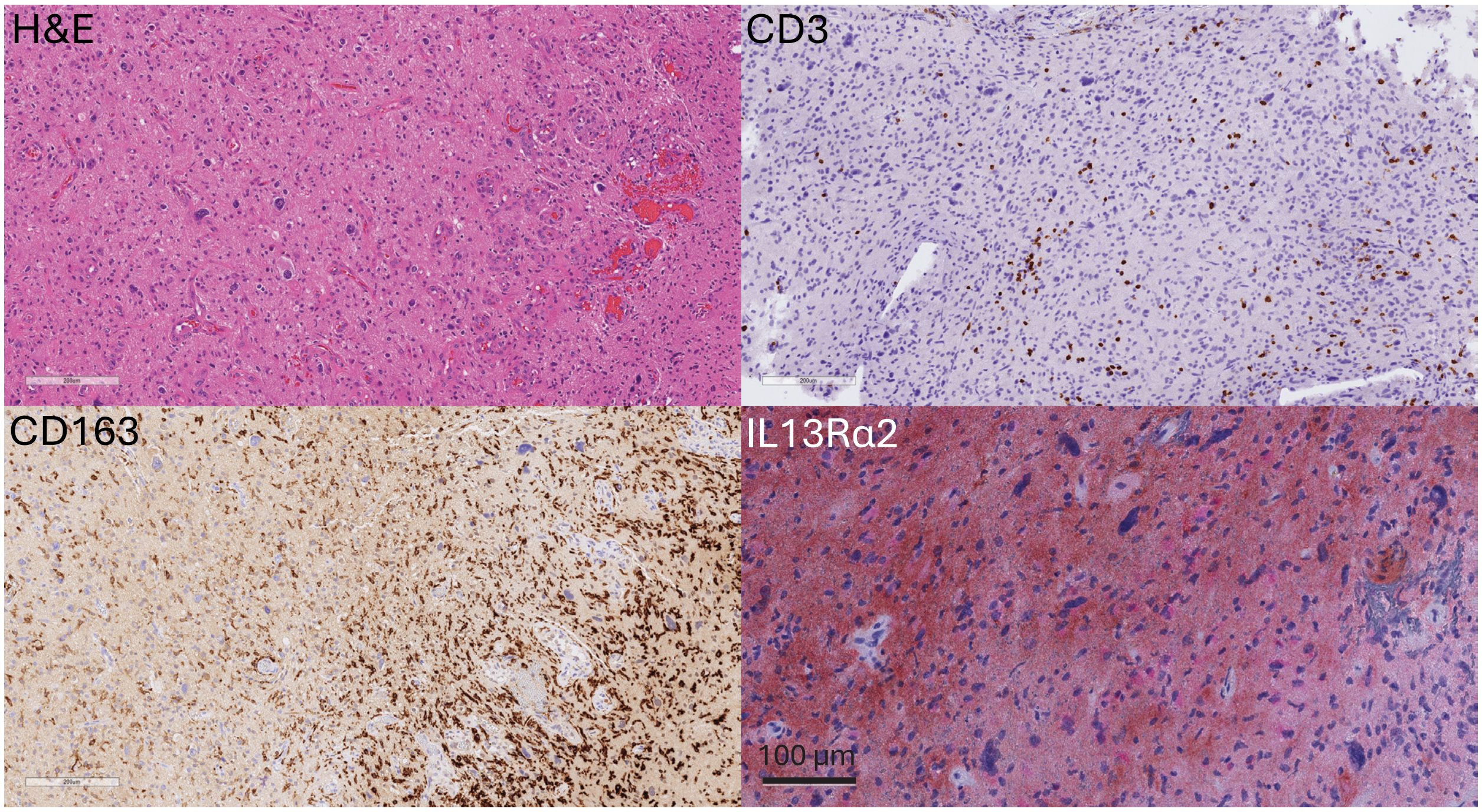

16321-52, P13, Pre-infusion

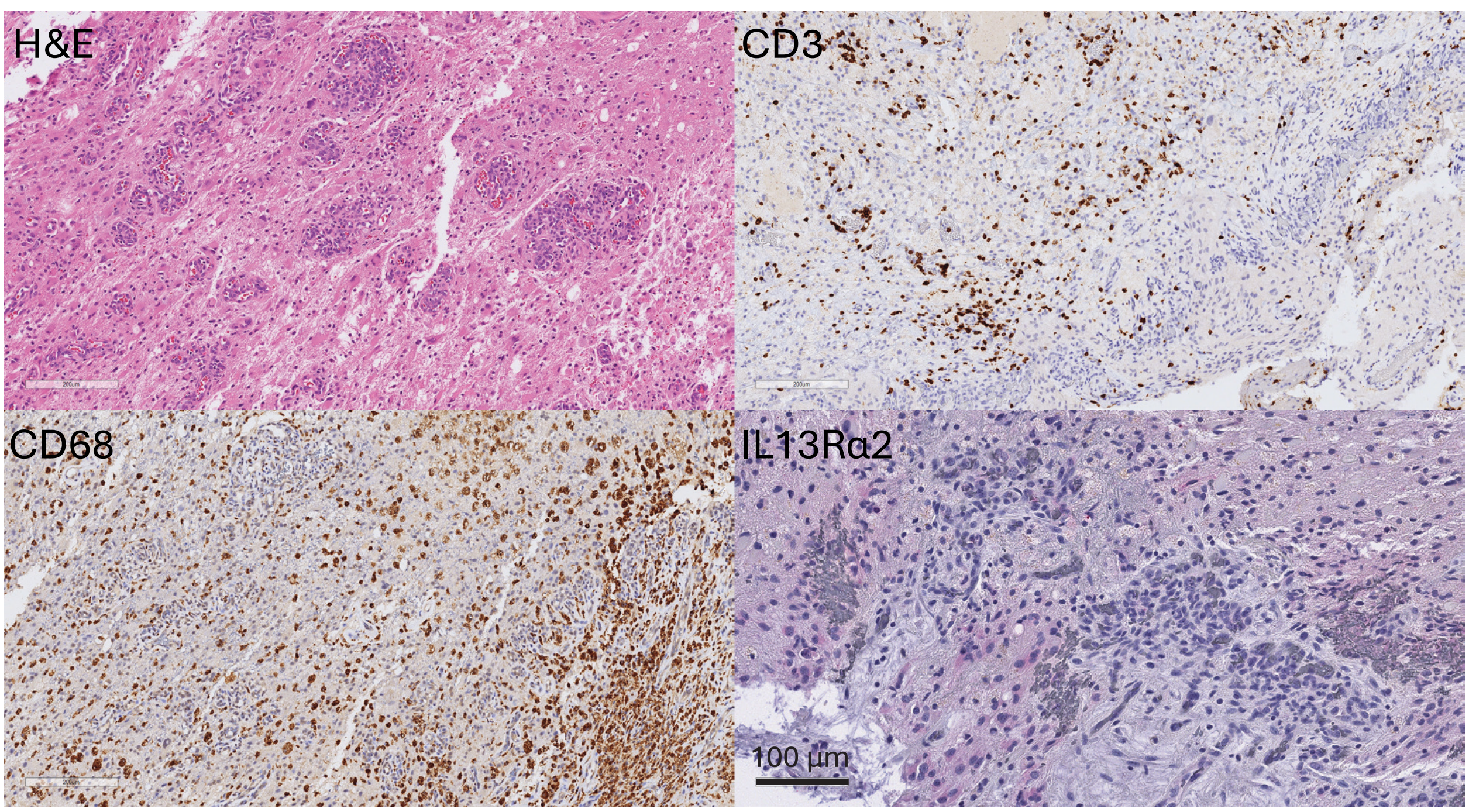

16321-52, P13, Post-infusion

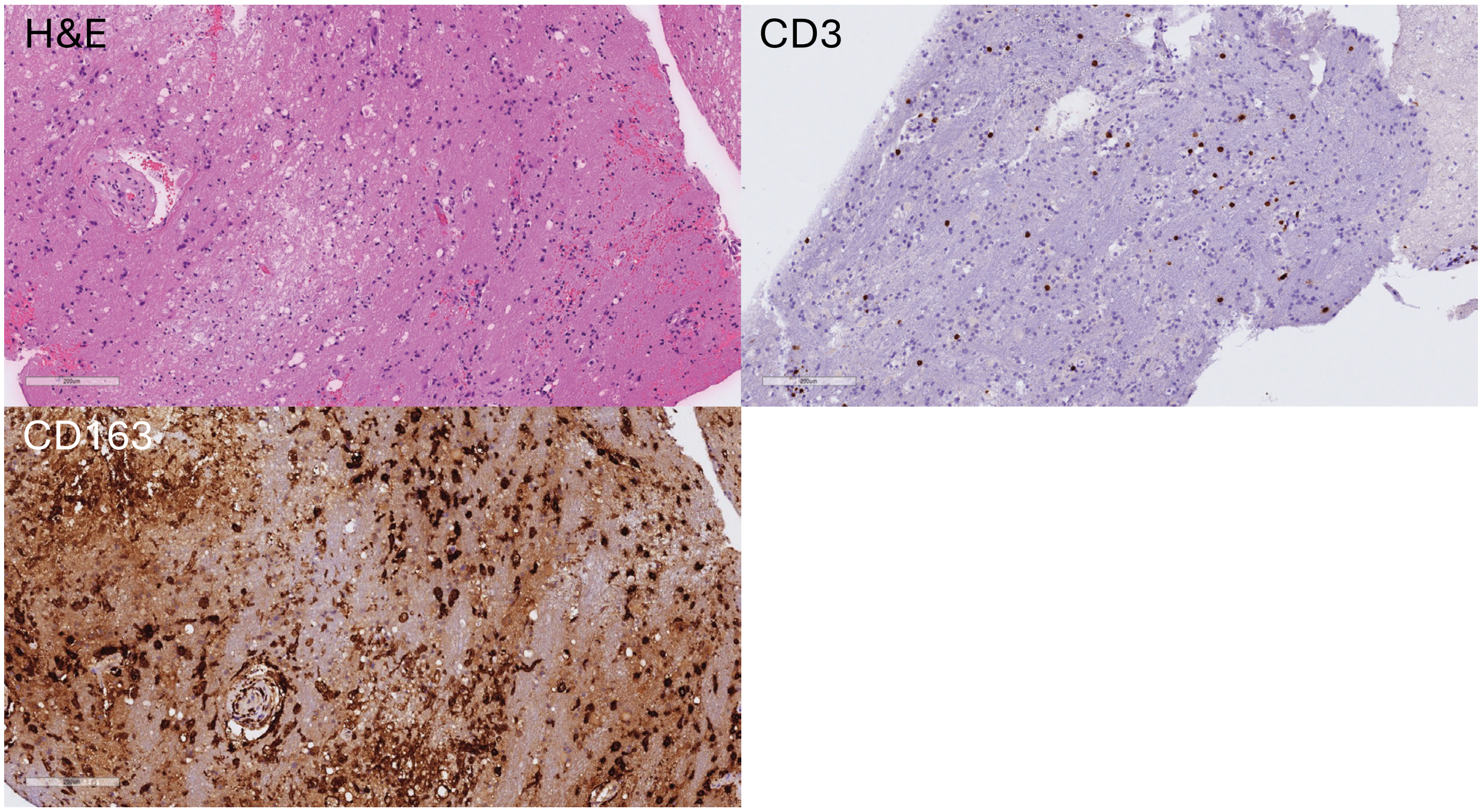

16321-55, P14, Pre-infusion

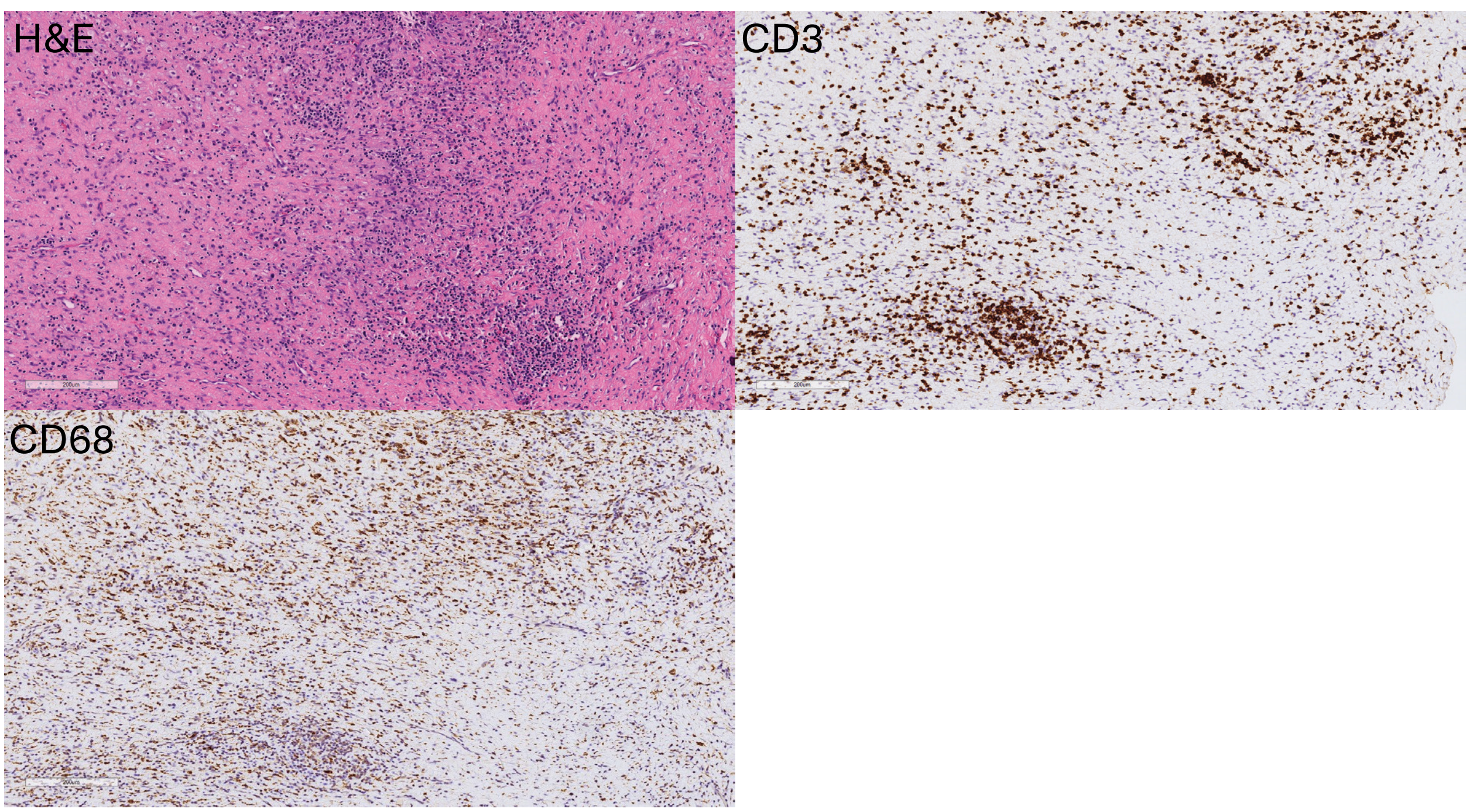

16321-55, P14, Post-infusion

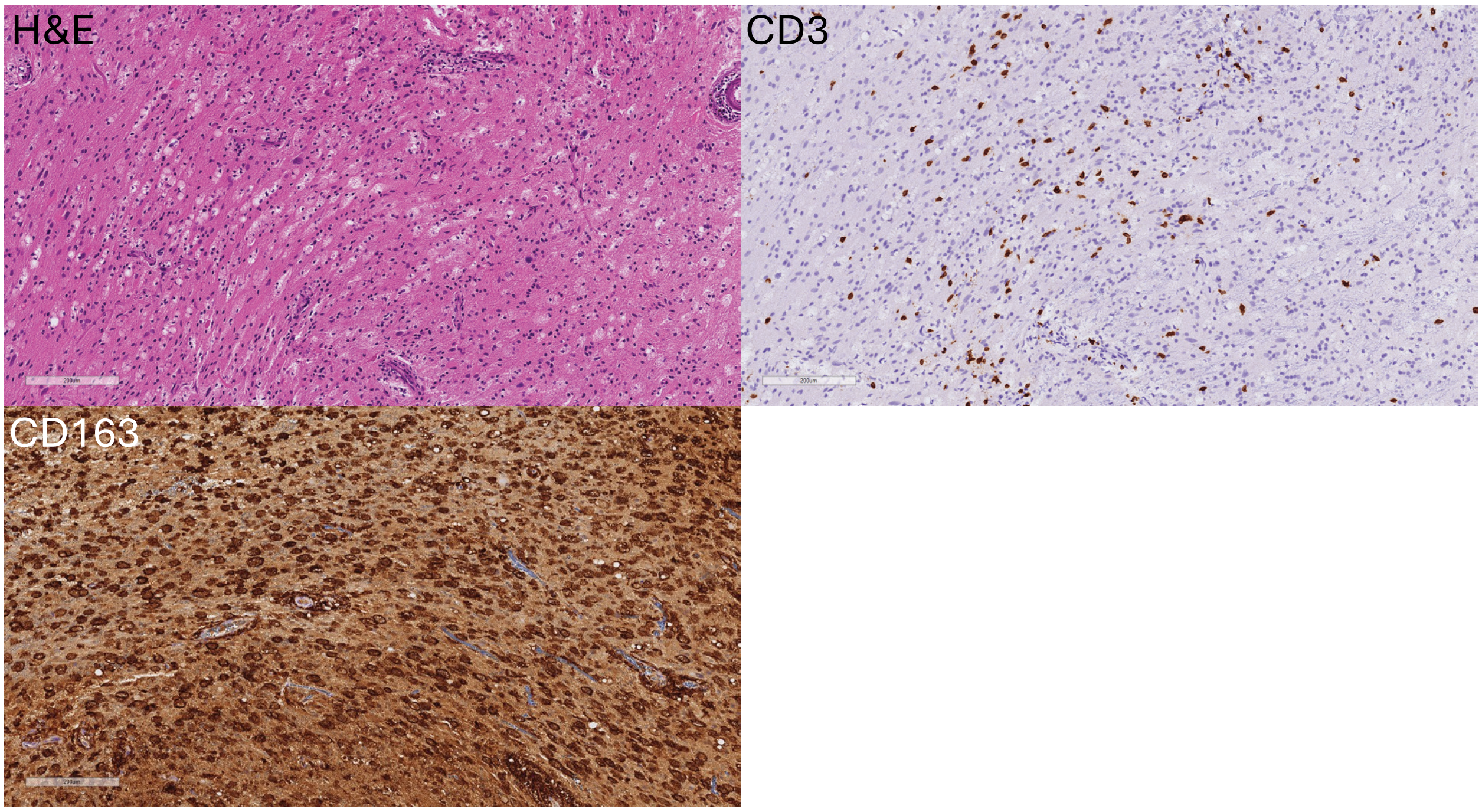

16321-47, P16, Pre-infusion

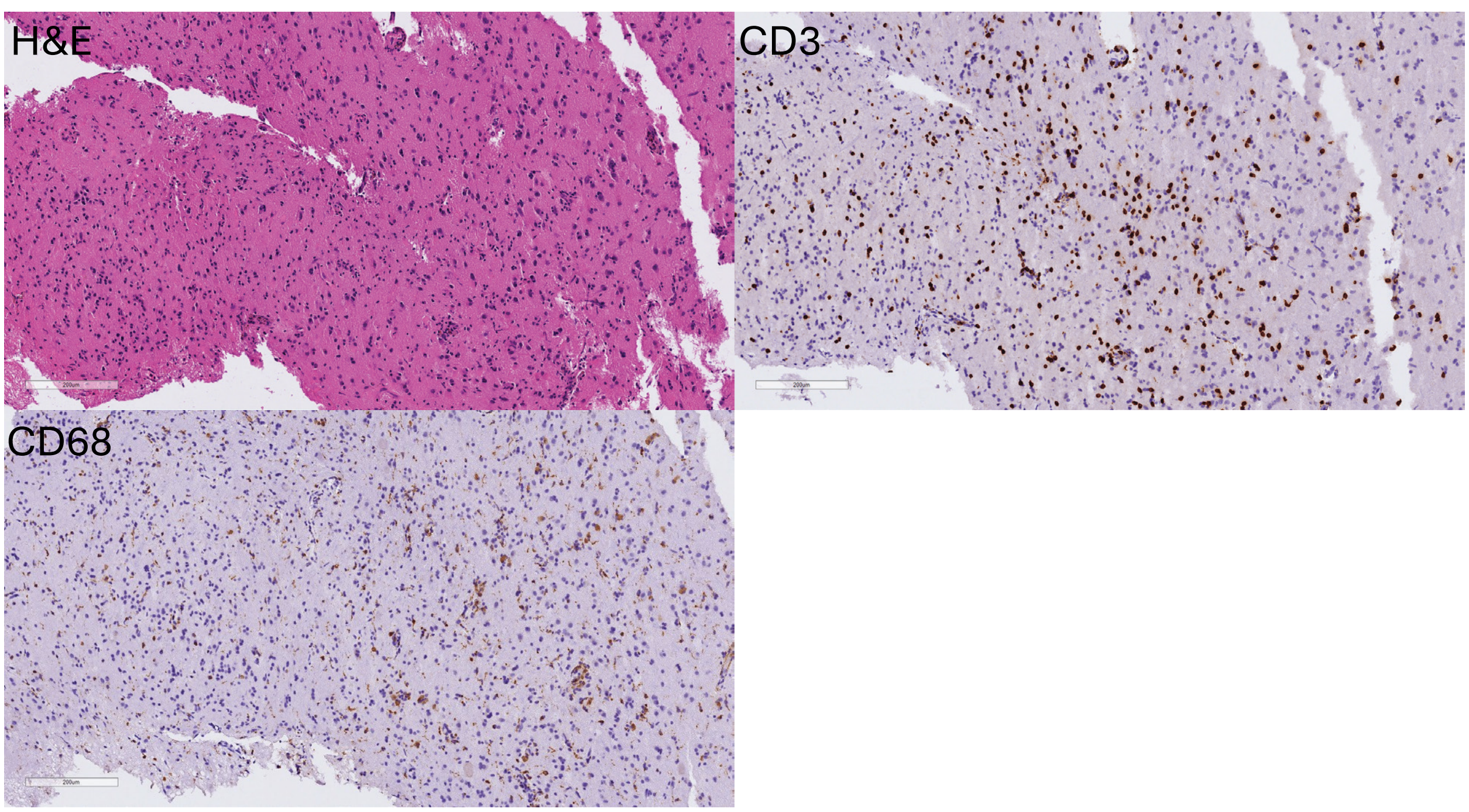

16321-47, P16, Post-infusion

3.2. EGFR in matched samples with enough residual tumor, by RNAscope and bulk RNA

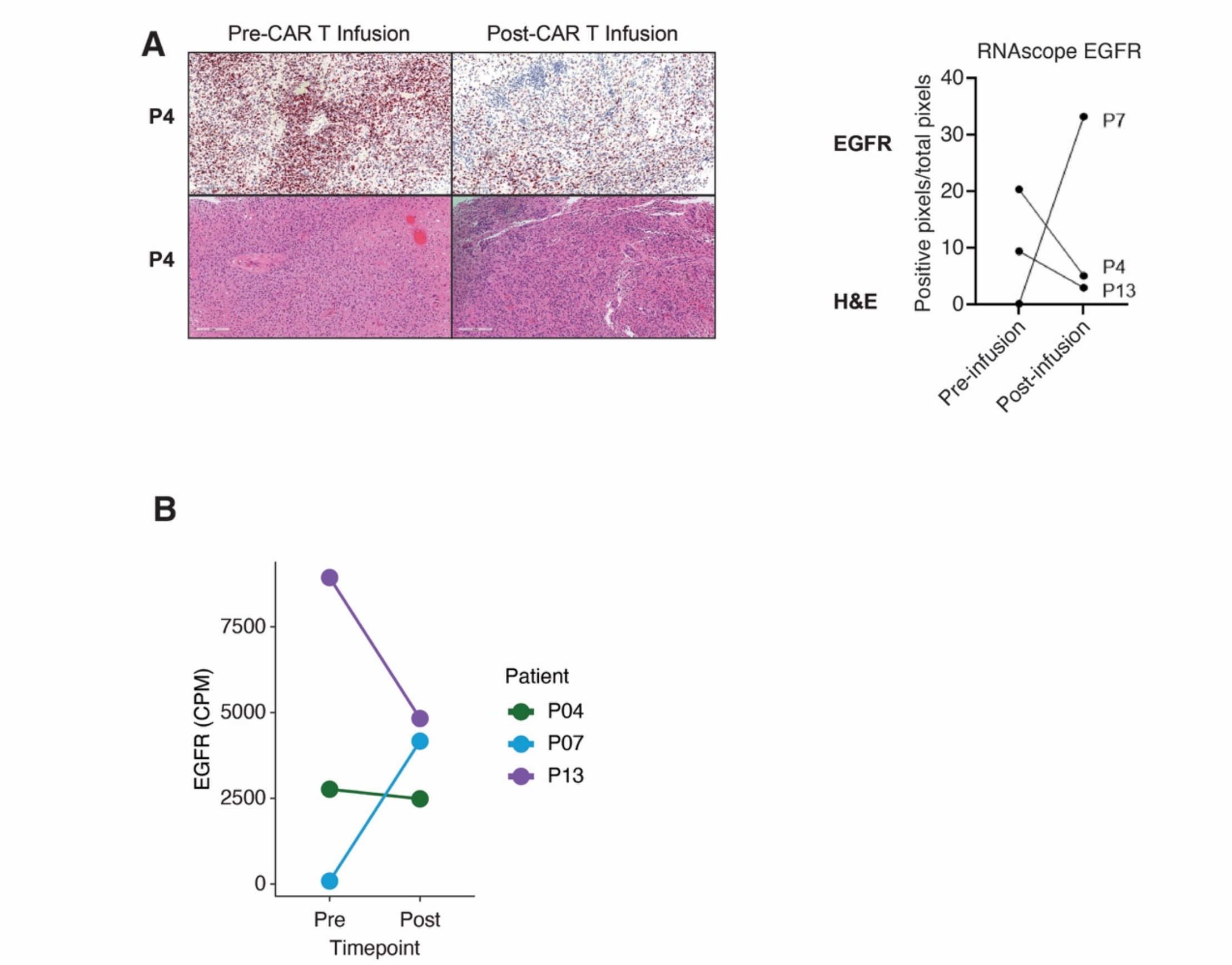

(A) EGFR expression in patient tumor expression by RNAscope on slides generated from FFPE tumor tissue (left, top), H&E stain (left, bottom), 5X magnification, and quantification (right).

(B) EGFR expression from bulk RNA sequencing (*n* = 3) from laser-capture microdissected tumor regions rich in malignant cells.

3.3. Gene expression of functional markers in CD8 T cells in matched tumor tissue

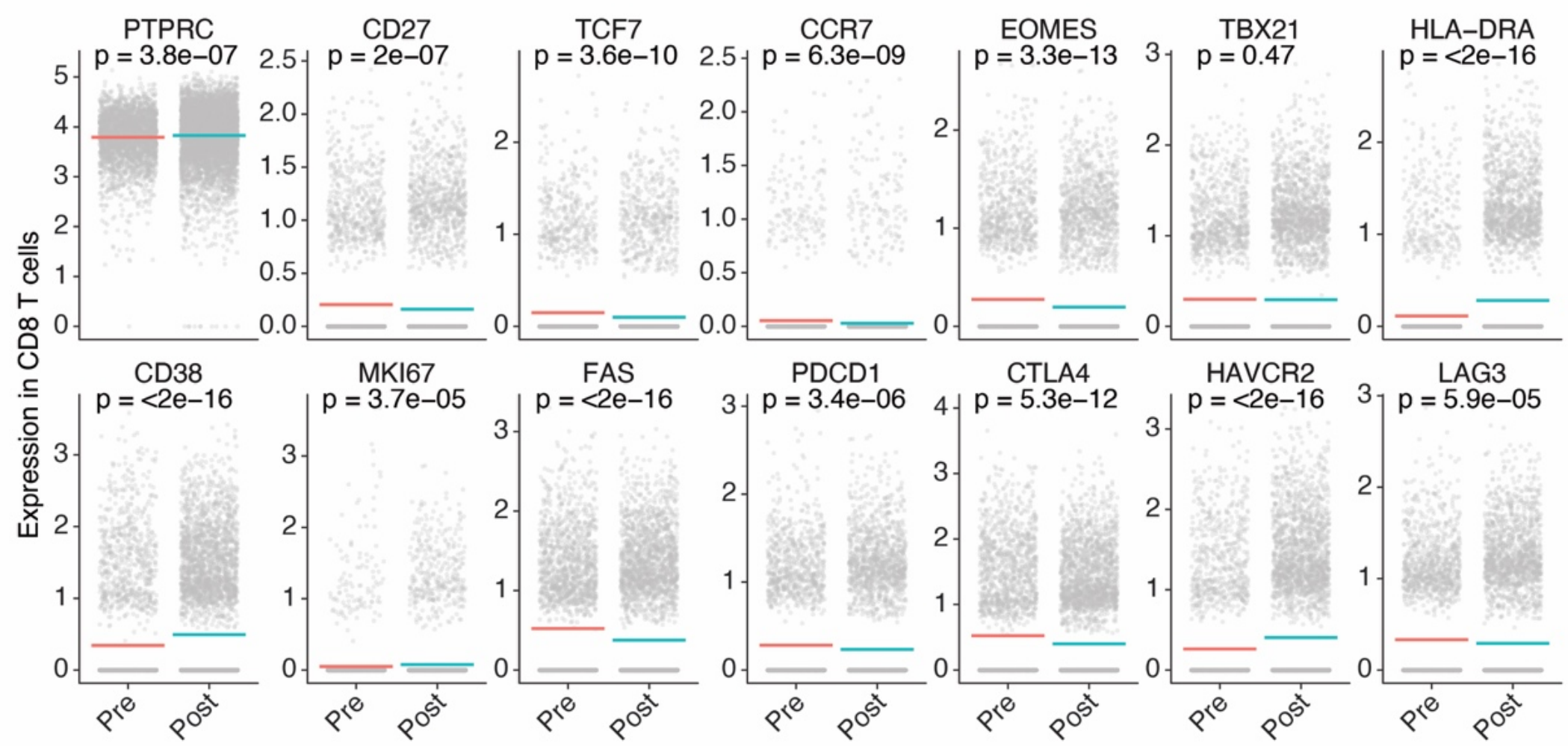

Expression of genes associated with T cell function in CD8 T cells (*n* = 8,966) in tumor single-nucleus RNA-seq data. Each dot represents a cell, and horizontal bars represent mean expression. P values from Wilcoxon tests.

4. Tumor NK cell annotation

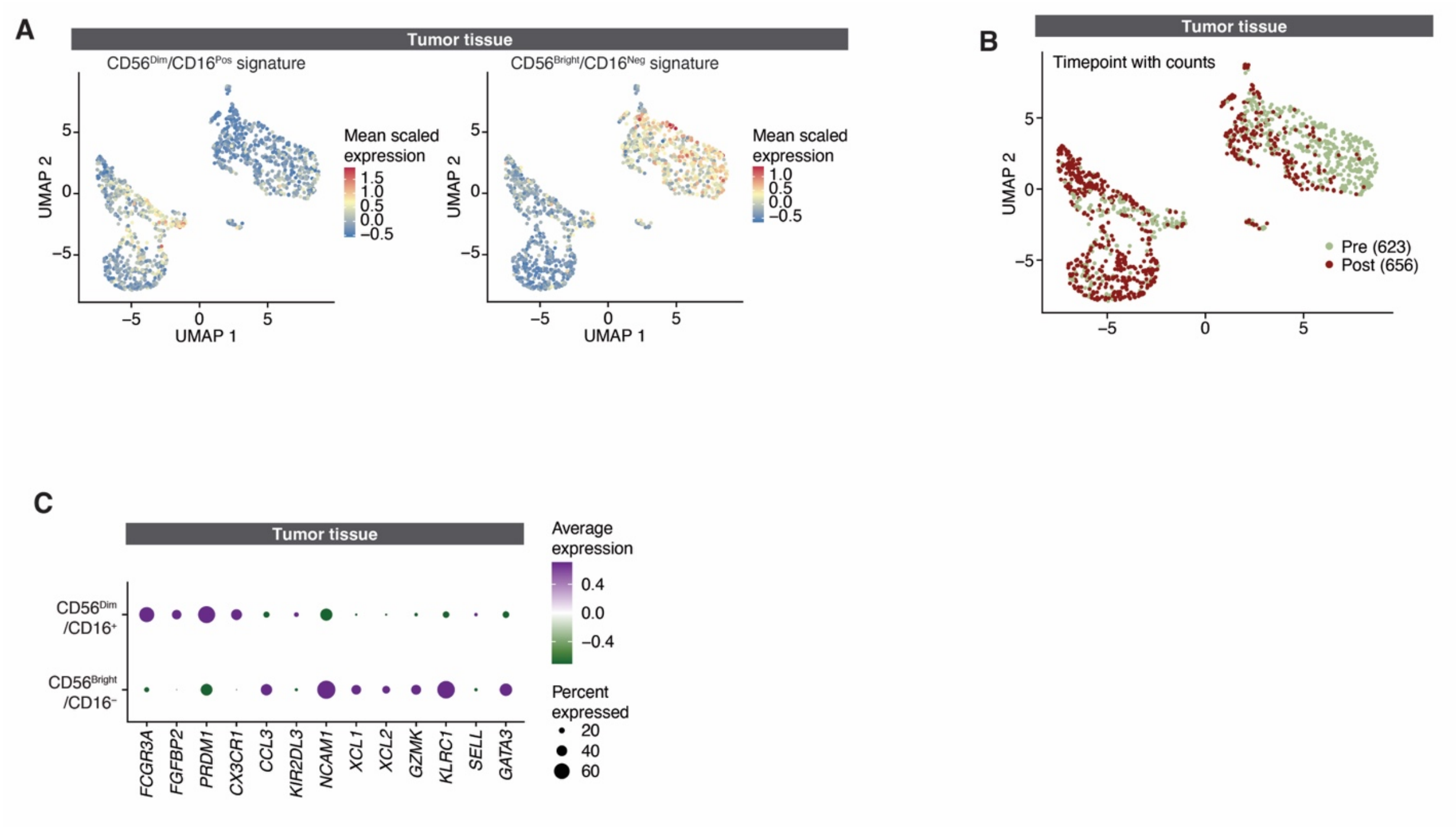

(A) UMAP visualization of tumor-infiltrating NK cells (*n* = 1279) in the tumor tissue, colored by expression of CD56^{dim}CD16^{pos} (left) and CD56^{bright}CD16^{neg} (right) gene signatures. Each point represents a single NK cell, colored by mean scaled expression of the corresponding signature.

(B) Tumor NK cells (*n* = 1279): UMAP colored by time point (I) and change in CD56^{dim}CD16^{pos} fraction (post - pre infusion) from the high-dose cohort (J; *n* = 2 time points from each of 3 patients).

(C) Marker gene expression for CD56^{bright}CD16^{neg} and CD56^{dim}CD16^{pos} NK cells in the tumor tissue. Dot size indicates the percentage of cells expressing each gene; color represents average expression level.

5. Steroid Management

The high-dose cohort received a uniform regimen, while variable steroid dosing in the low-dose cohort showed no correlation with cell population changes.

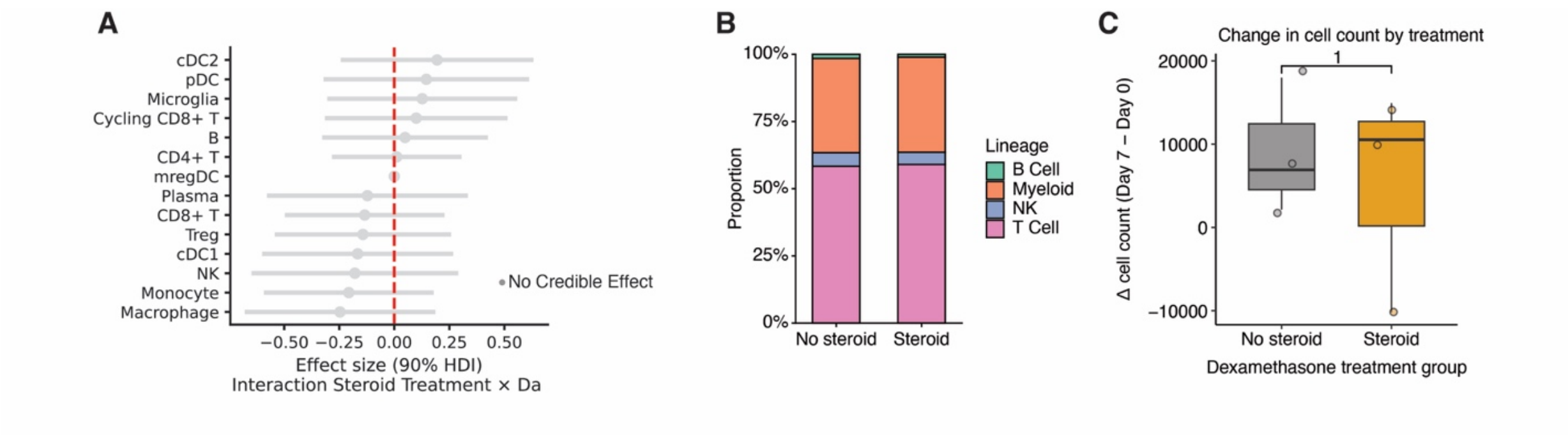

(A) Steroid treatment dependent change in cell composition Day 0 to 7 modeled by interaction estimates (Steroid x Day 7) in the low-dose cohort. Points show estimated log fold-change with 90% HDI; green indicates a credible effect, gray indicates none.

(B) Proportion of major cell lineages in steroid-treated versus steroid-spared patients in the low-dose cohort.

(C) Change in total CSF cell count (Day 7 - 0) compared between steroid-treated and steroid-spared patients in the low-dose cohort. Boxes span the interquartile range (25th - 75th percentiles) with the median shown as a central line; whiskers extend to the most extreme values within 1.5 times IQR. P values from two-sided paired Wilcoxon signed-rank test.

7. Ligand-Receptor analysis

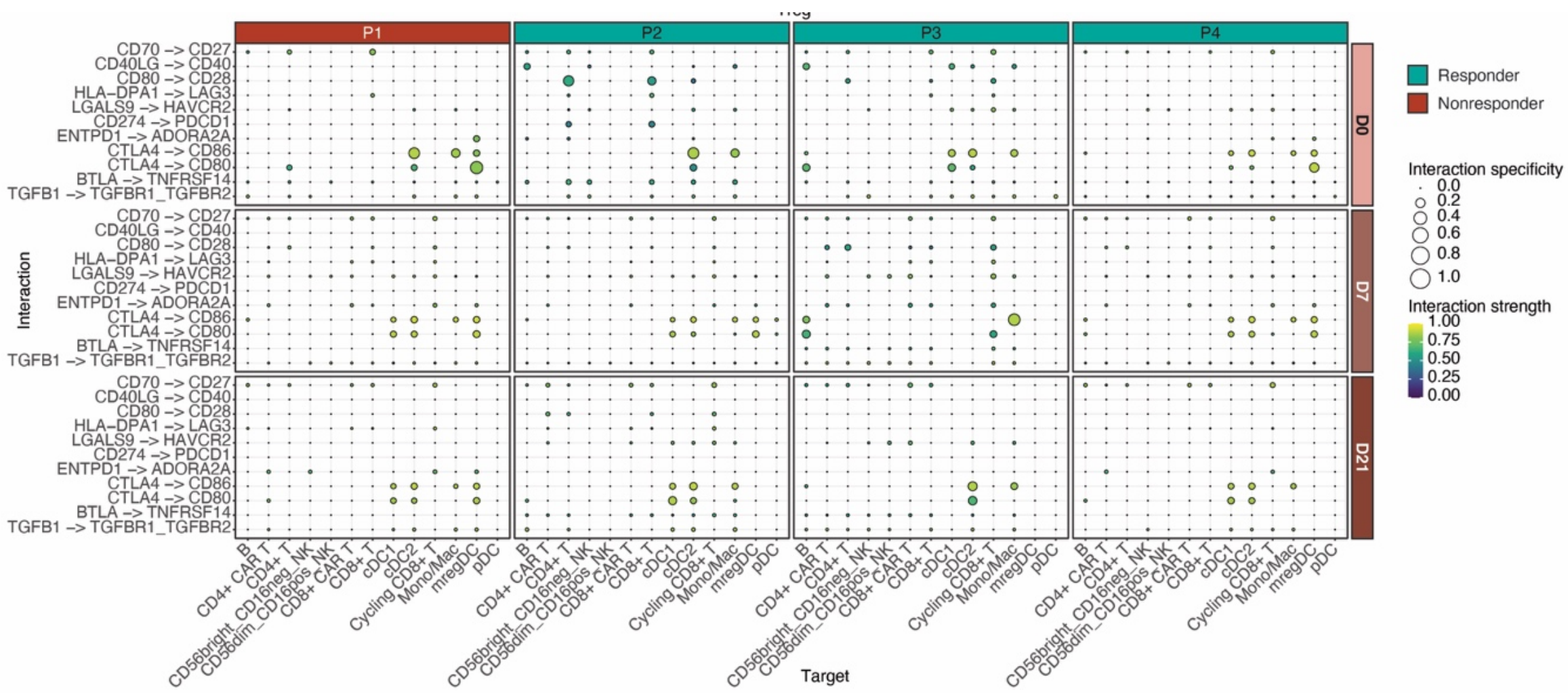

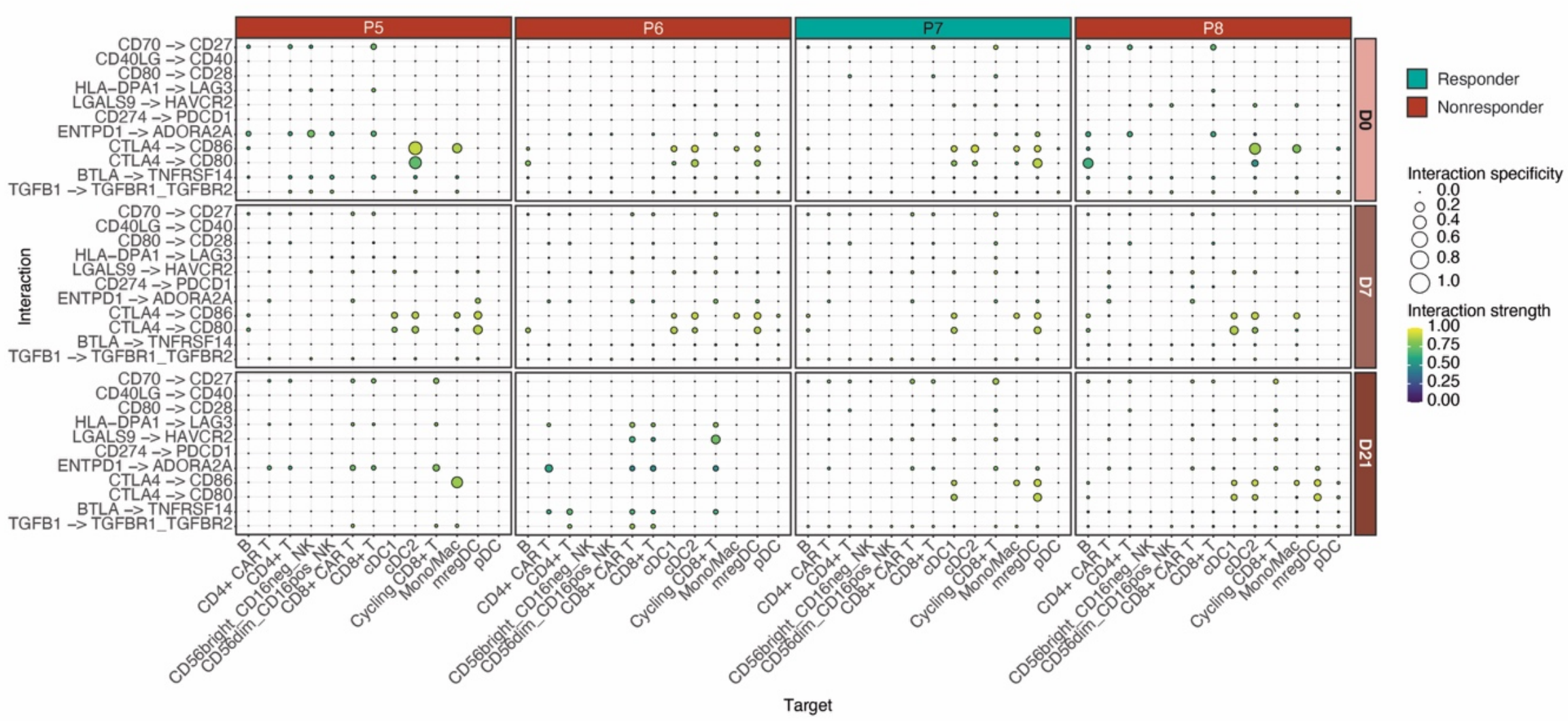

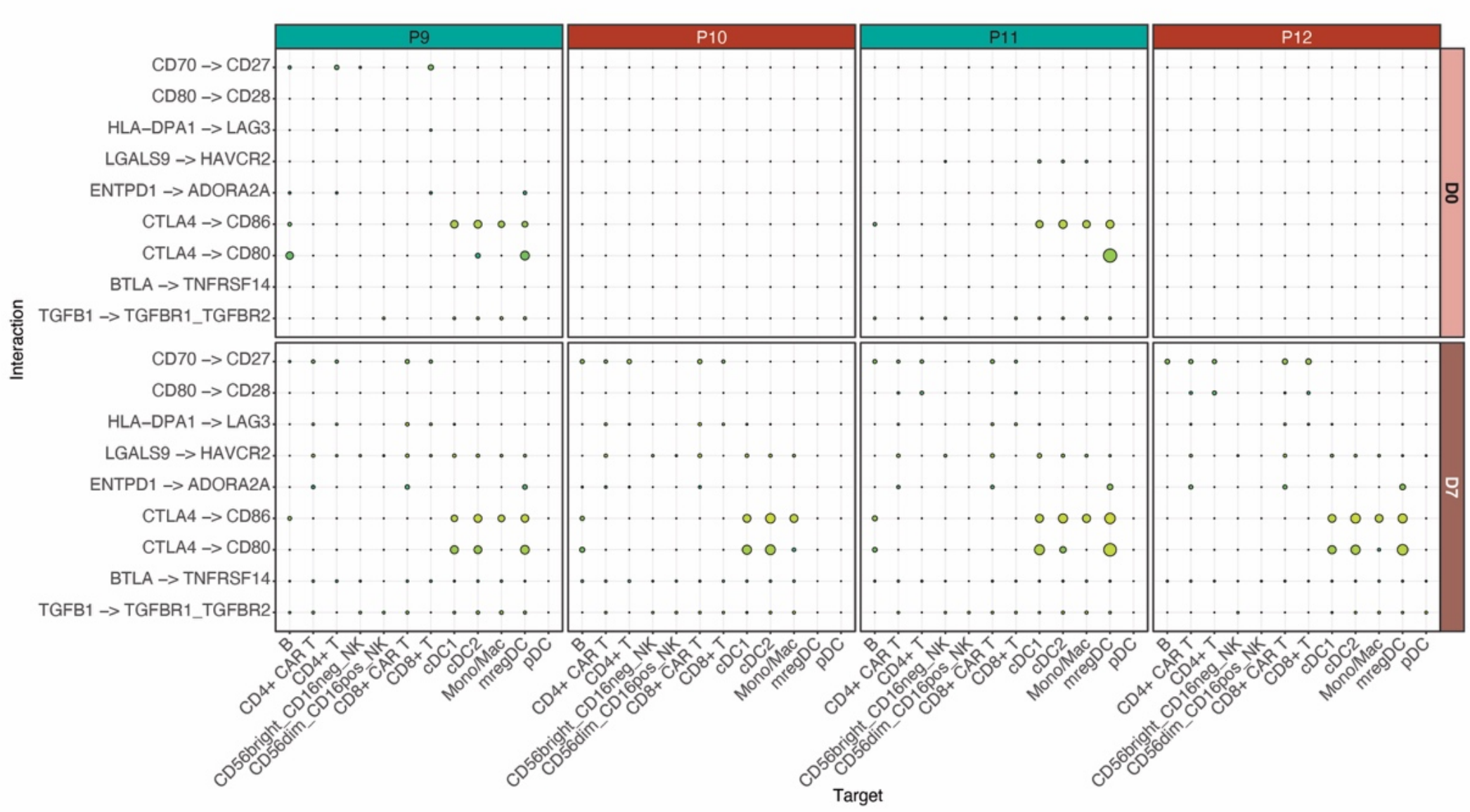

Ligand-receptor interaction map showing selected Treg interactions at Day 0, Day 7, Day 21 with indicated immune cell types in the CSF for each patient. Ligand-receptor interactions are indicated on the left in the format ligand -> receptor, where the Tregs express the ligand. Dot size corresponds to interaction specificity and color represents interaction strength. Patients 1–8 (discovery cohort) have three time points while patients 9-12 (validation cohort) have two.
