# Supplementary material for: The critical role of the endogenous immune compartment after CAR T cell therapy in recurrent GBM
Source: Cell. Author manuscript; Available in PMC 2026 Jul 27. (PMC13401735; doi:10.1016/j.cell.2026.05.026)
Supplement: Supplementary file 2 [file NIHMS2188461-supplement-4.pdf]

Figure S1

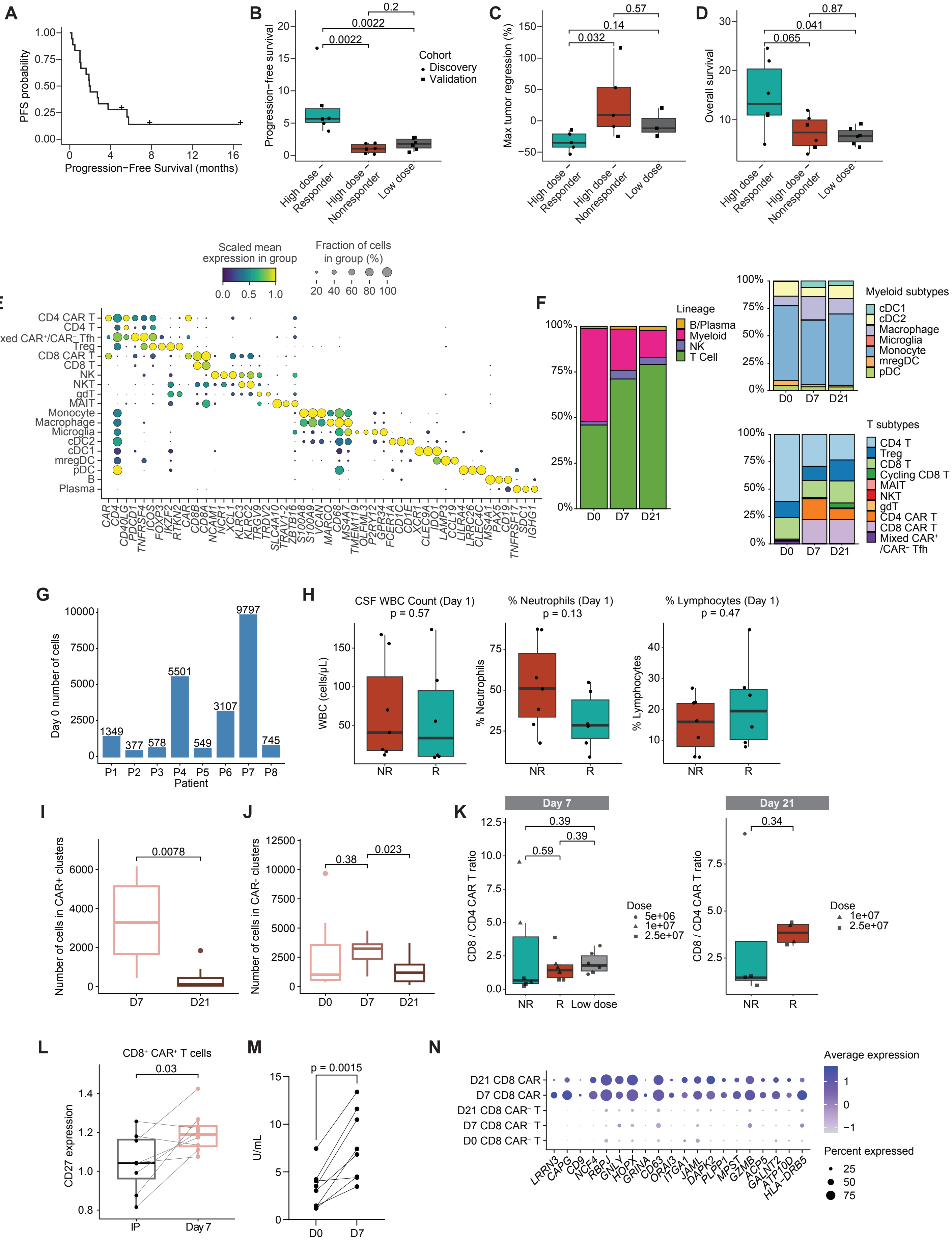

A
PFS probability
Progression−Free Survival (months)
B
Progression−free survival
Cohort
Discovery
Validation
High dose − Responder
High dose − Nonresponder
Low dose
C
Max tumor regression (%)
D
Overall survival
E
Scaled mean expression in group
Fraction of cells in group (%)
CD4 CAR T
CD4 T
Mixed CAR⁺/CAR⁻ Tfh
Treg
CD8 CAR T
CD8 T
NK
NKT
gdT
MAIT
Monocyte
Macrophage
Microglia
cDC2
cDC1
mregDC
pDC
B
Plasma
F
Lineage
B/Plasma
Myeloid
NK
T Cell
Myeloid subtypes
T subtypes
G
Day 0 number of cells
Patient
H
CSF WBC Count (Day 1) p = 0.57
% Neutrophils (Day 1) p = 0.13
% Lymphocytes (Day 1) p = 0.47
WBC (cells/μL)
% Neutrophils
% Lymphocytes
I
Number of cells in CAR+ clusters
J
Number of cells in CAR- clusters
K
Day 7
Day 21
CD8 / CD4 CAR T ratio
Dose
L
CD8⁺ CAR⁺ T cells
CD27 expression
M
U/mL
p = 0.0015
N
Average expression
Percent expressed
D21 CD8 CAR
D7 CD8 CAR
D21 CD8 CAR⁻ T
D7 CD8 CAR⁻ T
D0 CD8 CAR⁻ T

Figure S1. Clinical trial design, patient outcomes, and baseline CSF cellularity, related to Figure 1

(A) Kaplan–Meier curve showing the percentage of patients remaining progression-free over time following treatment (*n* = 18 patients, full cohort).
(B-D) Boxplot by High-Dose responders, High-Dose non-responders and Low-Dose showing of (**B**) progression-free survival (PFS) in months, (**C**) maximum percent reduction in tumor size during the first month after CAR T cell infusion, measured by MRI, and (**D**) Overall survival (OS) in months (*n* = 18 patients in each panel).
(E) Marker genes used to annotate immune cell types. Dot size: percent expressing; color: average expression.
(F) Cell type composition by day (*n* = 18 patients).
(G) Quantification of absolute cell recovery from CSF samples at baseline (D0).
(H) Count of White blood cells (WBC), percentage Neutrophils, and percentage Lymphocytes obtained from patient medical records on D1 CSF, responders vs non-responders, (n =12, High-dose).
(I and J) Number of cells in (**I**) CAR+ and (**J**) CAR- clusters per patient at D0, D7 and D21 (Discovery cohort).
(K) Ratio of CD8 to CD4 T cells in CAR+ clusters in the CSF D7 in responders (*n* = 6), non-responders (*n* = 6) and Low-Dose (*n* = 6) and D21 in responders (*n* = 6), and non-responders (*n* = 6). Low-Dose CSF was not processed for D21.
(L) CD27 expression in CD8 CAR T cells in IP and D7 CSF (*n* = 8, discovery cohort), as measured by scRNA-seq.
(M) Quantitative levels of sCD27 in D0 and D7 CSF as measured by ELISA (*n* = 8, discovery cohort).
(N) Top genes contributing to enrichment of the neoantigen-reactive TIL signature in CD8 CAR T cells as in Fig1I. Dot size indicates the percentage of cells expressing each gene; color represents average expression level.

Data in (**B-D, H-L**) Boxes span the interquartile range (25th - 75th percentiles) with the median shown as a central line; whiskers extend to the most extreme values within 1.5 times IQR. P values: paired Wilcoxon signed-rank (**I, J, L**), two-sided Wilcoxon rank-sum test (**B–D, H, K**), or two-sided paired t-test (**M**).

Figure S2

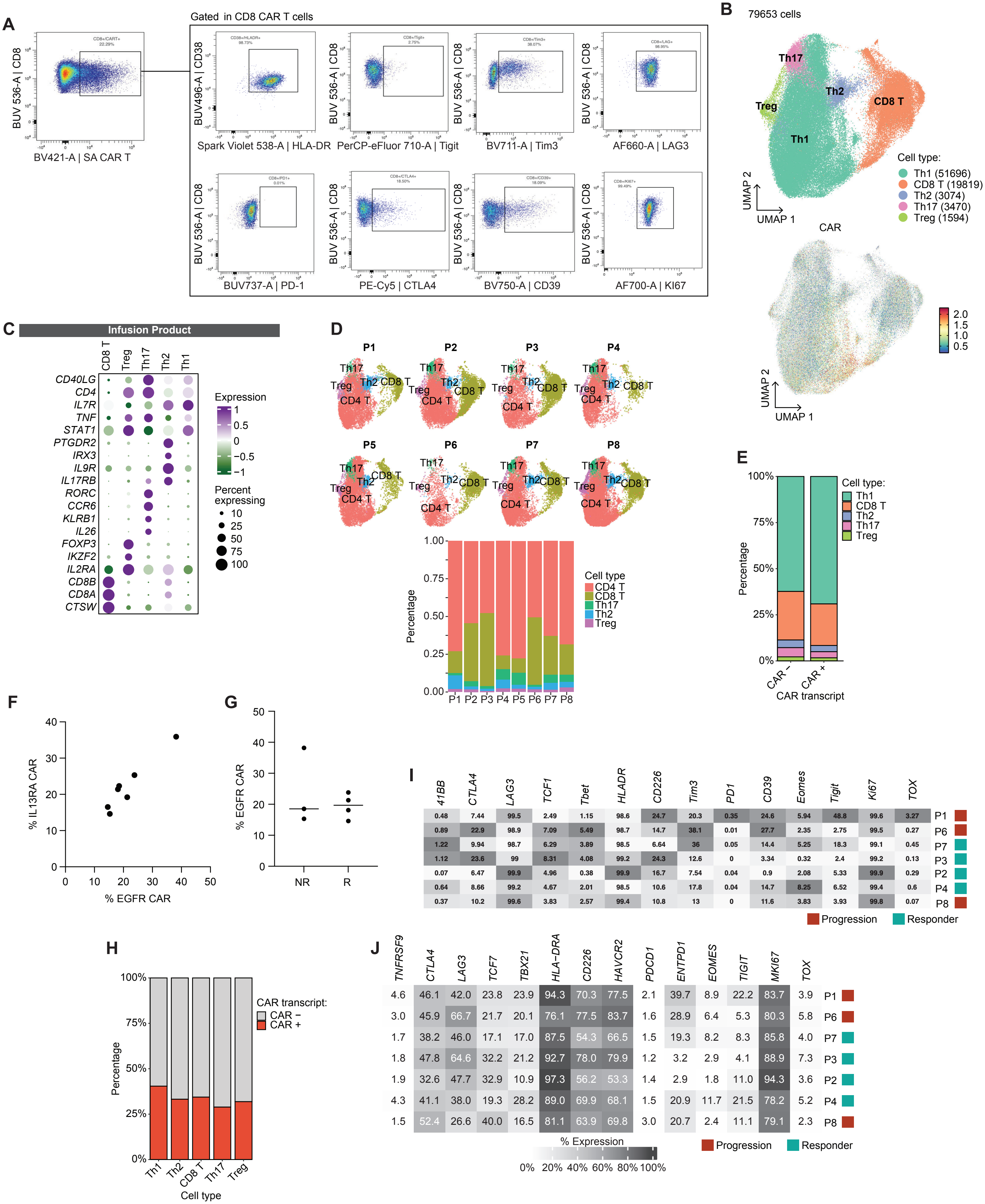

A
Gated in CD8 CAR T cells
BUV 536-A | CD8
BV421-A | SA CAR T
BUV496-A | CD38
Spark Violet 538-A | HLA-DR
PerCP-eFluor 710-A | Tigit
BV711-A | Tim3
AF660-A | LAG3
BUV737-A | PD-1
PE-Cy5 | CTLA4
BV750-A | CD39
AF700-A | KI67
B
79653 cells
Th17
Th2
Treg
CD8 T
Th1
UMAP 2
UMAP 1
Cell type:
Th1 (51696)
CD8 T (19819)
Th2 (3074)
Th17 (3470)
Treg (1594)
CAR
2.0
1.5
1.0
0.5
C
Infusion Product
CD8 T
Treg
Th17
Th2
Th1
CD40LG
CD4
IL7R
TNF
STAT1
PTGDR2
IRX3
IL9R
IL17RB
RORC
CCR6
KLRB1
IL26
FOXP3
IKZF2
IL2RA
CD8B
CD8A
CTSW
Expression
Percent expressing
10
25
50
75
100
D
P1
P2
P3
P4
P5
P6
P7
P8
Th17
Treg
Th2
CD8 T
CD4 T
Percentage
Cell type
CD4 T
CD8 T
Th17
Th2
Treg
E
Percentage
Cell type:
Th1
CD8 T
Th2
Th17
Treg
CAR –
CAR +
CAR transcript
F
% IL13RA CAR
% EGFR CAR
G
% EGFR CAR
NR
R
H
Percentage
CAR transcript:
CAR –
CAR +
Th1
Th2
CD8 T
Th17
Treg
Cell type
I
41BB | CTLA4 | LAG3 | TCF1 | Tbet | HLADR | CD226 | Tim3 | PD1 | CD39 | Eomes | Tigit | Ki67 | TOX
0.48 | 7.44 | 99.5 | 2.49 | 1.15 | 98.6 | 24.7 | 20.3 | 0.35 | 24.6 | 5.94 | 48.8 | 99.6 | 3.27 | P1
0.89 | 22.9 | 98.9 | 7.09 | 5.49 | 98.7 | 14.7 | 38.1 | 0.01 | 27.7 | 2.35 | 2.75 | 99.5 | 0.27 | P6
1.22 | 9.94 | 98.7 | 6.29 | 3.89 | 98.5 | 6.64 | 36 | 0.05 | 14.4 | 5.25 | 18.3 | 99.1 | 0.45 | P7
1.12 | 23.6 | 99 | 8.31 | 4.08 | 99.2 | 24.3 | 12.6 | 0 | 3.34 | 0.32 | 2.4 | 99.2 | 0.13 | P3
0.07 | 6.47 | 99.9 | 4.96 | 0.38 | 99.9 | 16.7 | 7.54 | 0.04 | 0.9 | 2.08 | 5.33 | 99.9 | 0.29 | P2
0.64 | 8.66 | 99.2 | 4.67 | 2.01 | 98.5 | 10.6 | 17.8 | 0.04 | 14.7 | 8.25 | 6.52 | 99.4 | 0.6 | P4
0.37 | 10.2 | 99.6 | 3.83 | 2.57 | 99.4 | 10.8 | 13 | 0 | 11.6 | 3.83 | 3.93 | 99.8 | 0.07 | P8
Progression
Responder
J
TNFRSF9 | CTLA4 | LAG3 | TCF7 | TBX21 | HLA-DRA | CD226 | HAVCR2 | PDCD1 | ENTPD1 | EOMES | TIGIT | MKI67 | TOX
4.6 | 46.1 | 42.0 | 23.8 | 23.9 | 94.3 | 70.3 | 77.5 | 2.1 | 39.7 | 8.9 | 22.2 | 83.7 | 3.9 | P1
3.0 | 45.9 | 66.7 | 21.7 | 20.1 | 76.1 | 77.5 | 83.7 | 1.6 | 28.9 | 6.4 | 5.3 | 80.3 | 5.8 | P6
1.7 | 38.2 | 46.0 | 17.1 | 17.0 | 87.5 | 54.3 | 66.5 | 1.5 | 19.3 | 8.2 | 8.3 | 85.8 | 4.0 | P7
1.8 | 47.8 | 64.6 | 32.2 | 21.2 | 92.7 | 78.0 | 79.9 | 1.2 | 3.2 | 2.9 | 4.1 | 88.9 | 7.3 | P3
1.9 | 32.6 | 47.7 | 32.9 | 10.9 | 97.3 | 56.2 | 53.3 | 1.4 | 2.9 | 1.8 | 11.0 | 94.3 | 3.6 | P2
4.3 | 41.1 | 38.0 | 19.3 | 28.2 | 89.0 | 69.9 | 68.1 | 1.5 | 20.9 | 11.7 | 21.5 | 78.2 | 5.2 | P4
1.5 | 52.4 | 26.6 | 40.0 | 16.5 | 81.1 | 63.9 | 69.8 | 3.0 | 20.7 | 2.4 | 11.1 | 79.1 | 2.3 | P8
% Expression
0% 20% 40% 60% 80% 100%
Progression
Responder

Figure S2. Infusion product composition and phenotype, related to Figure 2.
(A) Representative example of flow cytometry plots showing markers of activation (HLA-DR, CD38), proliferation (Ki-67) and exhaustion (PD-1, CTLA-4, Tigit, Tim3, CD39, LAG3) in CD8 CAR T cells in the infusion product (here, Patient 6).
(B) UMAP embedding of infusion product cells from scRNA-seq of the first 8 patients (discovery cohort, n = 79,653 cells) annotated by cell type (left) and CAR transcript count (right).
(C) Gene expression of key lineage-specific genes used to identify major T cell subpopulations in the infusion product scRNA-seq data.
(D) UMAP in (B) subsetted per patient and column bar showing per patient composition
(E) Relative abundance of the different immune cell types in the infusion product, stratified by CAR transcript detection status (CAR+ vs CAR-), based on the scRNA-seq data.
(F) Scatterplot showing proportions of EGFR-CAR and IL13Rα2-CAR T cells within the CD8 T cells of the infusion products, as determined by two separate stainings with corresponding biotinylated proteins, measured by spectral cytometry (n = 7 patients, discovery cohort).
(G) Proportions of EGFR-CAR T cells in the CD8 T cells within infusion products of responders (R) and non-responders (NR), measured by spectral cytometry. Line represents median (n = 7 patients, discovery cohort).
(H) Proportion of cells with detected CAR transcript within each T cell subset across patients, measured by the infusion product scRNA-seq data.
(I) Heatmap of surface marker expression in CD8 CAR T cells from the infusion product, based on the 30-marker spectral cytometry panel (n = 7 patients, discovery cohort).
(J) Heatmap of gene expression for activation, exhaustion, and proliferation markers in CD8 T cells with detectable CAR transcripts from infusion products, based on the infusion product scRNA-seq data (n = 8 patients, discovery cohort).

Figure S3

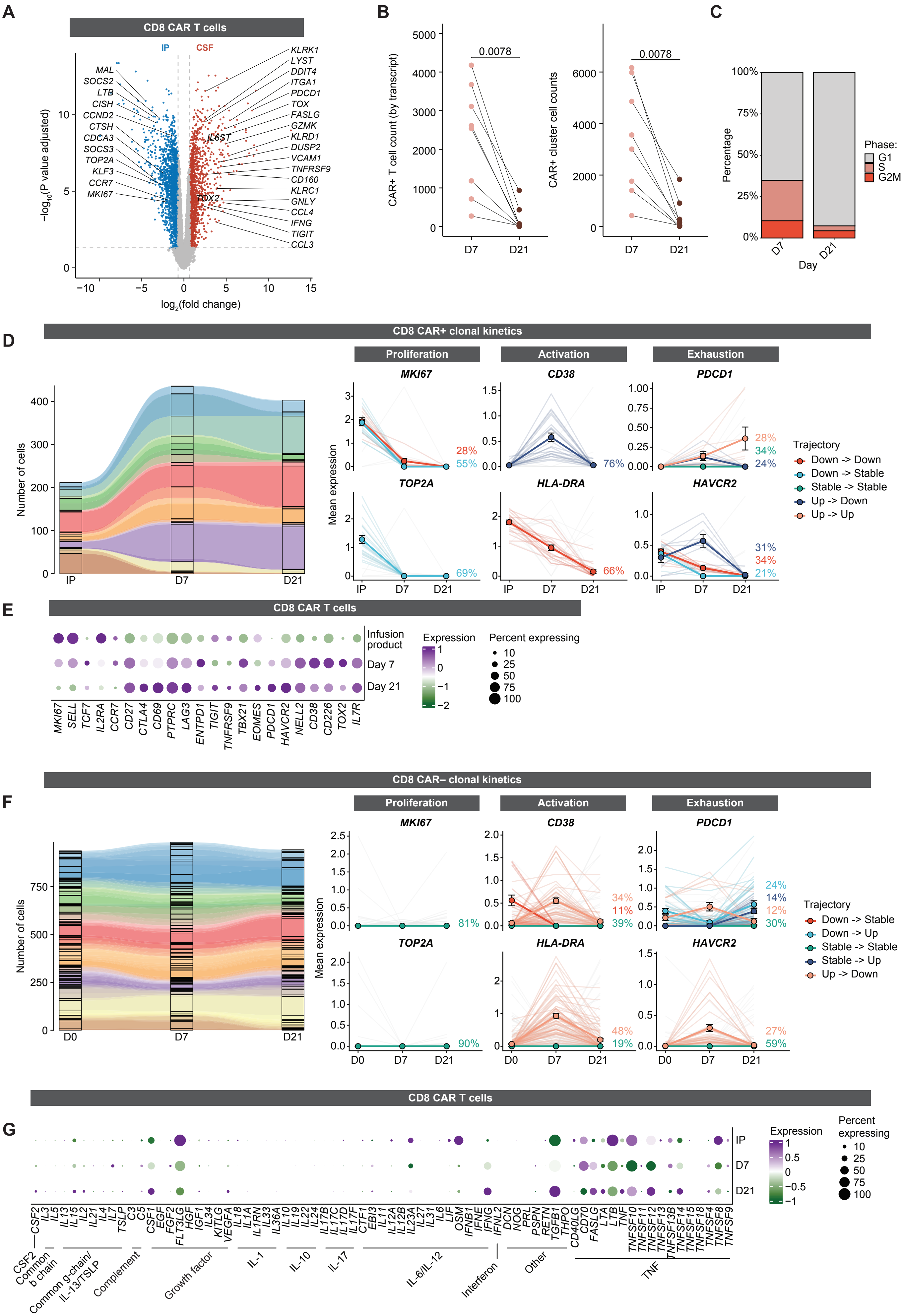
A
CD8 CAR T cells
IP
CSF
MAL
SOCS2
LTB
CISH
CCND2
CTSH
CDCA3
SOCS3
TOP2A
KLF3
CCR7
MKI67
KLRK1
LYST
DDIT4
ITGA1
PDCD1
TOX
FASLG
GZMK
KLRD1
DUSP2
VCAM1
TNFRSF9
CD160
KLRC1
GNLY
CCL4
IFNG
TIGIT
CCL3
IL6ST
TOX2
−log10(P value adjusted)
log2(fold change)
B
0.0078
CAR+ T cell count (by transcript)
CAR+ cluster cell counts
D7
D21
C
Percentage
Phase:
G1
S
G2M
Day
D
CD8 CAR+ clonal kinetics
Proliferation
Activation
Exhaustion
MKI67
CD38
PDCD1
TOP2A
HLA-DRA
HAVCR2
Number of cells
Mean expression
IP
D7
D21
Trajectory
Down -> Down
Down -> Stable
Stable -> Stable
Up -> Down
Up -> Up
E
CD8 CAR T cells
Infusion product
Day 7
Day 21
Expression
Percent expressing
MKI67
SELL
TCF7
IL2RA
CCR7
CD27
CTLA4
CD69
PTPRC
LAG3
ENTPD1
TIGIT
TNFRSF9
TBX21
EOMES
PDCD1
HAVCR2
NELL2
CD38
CD226
TOX2
IL7R
F
CD8 CAR− clonal kinetics
Proliferation
Activation
Exhaustion
Number of cells
Mean expression
D0
D7
D21
Trajectory
Down -> Stable
Down -> Up
Stable -> Stable
Stable -> Up
Up -> Down
G
CD8 CAR T cells
IP
D7
D21
Expression
Percent expressing
CSF2
IL3
IL5
IL13
IL15
IL2
IL21
IL4
IL7
TSLP
C3
C5
CSF1
EGF
FGF2
FLT3LG
HGF
IGF1
IL34
KITLG
VEGFA
IL18
IL1A
IL1RN
IL36A
IL10
IL19
IL22
IL24
IL17B
IL17C
IL17D
IL17F
CTF1
EBI3
IL11
IL12A
IL12B
IL23A
IL27
IL31
IL6
LIF
OSM
IFNB1
IFNE
IFNG
IFNL2
DCN
NOG
PRL
PSPN
RETN
TGFB1
THPO
CD40LG
CD70
FASLG
LTA
LTB
TNF
TNFSF10
TNFSF11
TNFSF12
TNFSF13
TNFSF13B
TNFSF14
TNFSF15
TNFSF18
TNFSF4
TNFSF8
TNFSF9
CSF2
Common b chain
Common g-chain/ IL-13/TSLP
Complement
Growth factor
IL-1
IL-10
IL-17
IL-6/IL-12
Interferon
Other
TNF

Figure S3. Longitudinal transcriptional remodeling of CD8 CAR T cells in the CSF, related to Figure 2.
(A) Differential gene expression analysis comparing CD8 CAR T cells in the CSF (all time points) and IP. P values from two-sided quasi-likelihood F-test of edgeR. Genes with Benjamini-Hochberg-adjusted $p < 0.05$ and absolute fold change > 2 are highlighted. Selected significant DE genes are labeled.
(B) Total number of CD8 CAR T cells detected at D7 and D21 across patients, as determined by presence of CAR transcripts (left) or assignment to CAR+ clusters (right). P values from two-sided paired Wilcoxon signed-rank tests.
(C) Cell cycle phase distribution of CD8 CAR T cells across time points.
(**D**) TCR-based shared clones and their activation kinetics. Bold colored lines and points denote the mean expression and SEM of trajectories with > 10% frequency and paired Wilcoxon signed-rank test $p < 0.05$ for CAR+ clones ($n = 29$ clones).
(E) Average expression (color) and percent of cells expressing (dot size) of the same key genes shown in **Fig 2E**, but restricted to the subset of CD8 CAR T cells with TCR clonotypes shared across all three timepoints (Infusion product, CSF D7, and CSF D21).
(**F**) Same as in (D), shown for CAR^{-} clones (n = 145 clones).
(G) Dot plot shows average expression (color) and percent of CD8 CAR T cells expressing each gene (dot size) across infusion product (IP), D7, and D21, highlighting cytokines and other soluble immune ligands.
(A-G) All panels are from the discovery cohort.

Figure S4

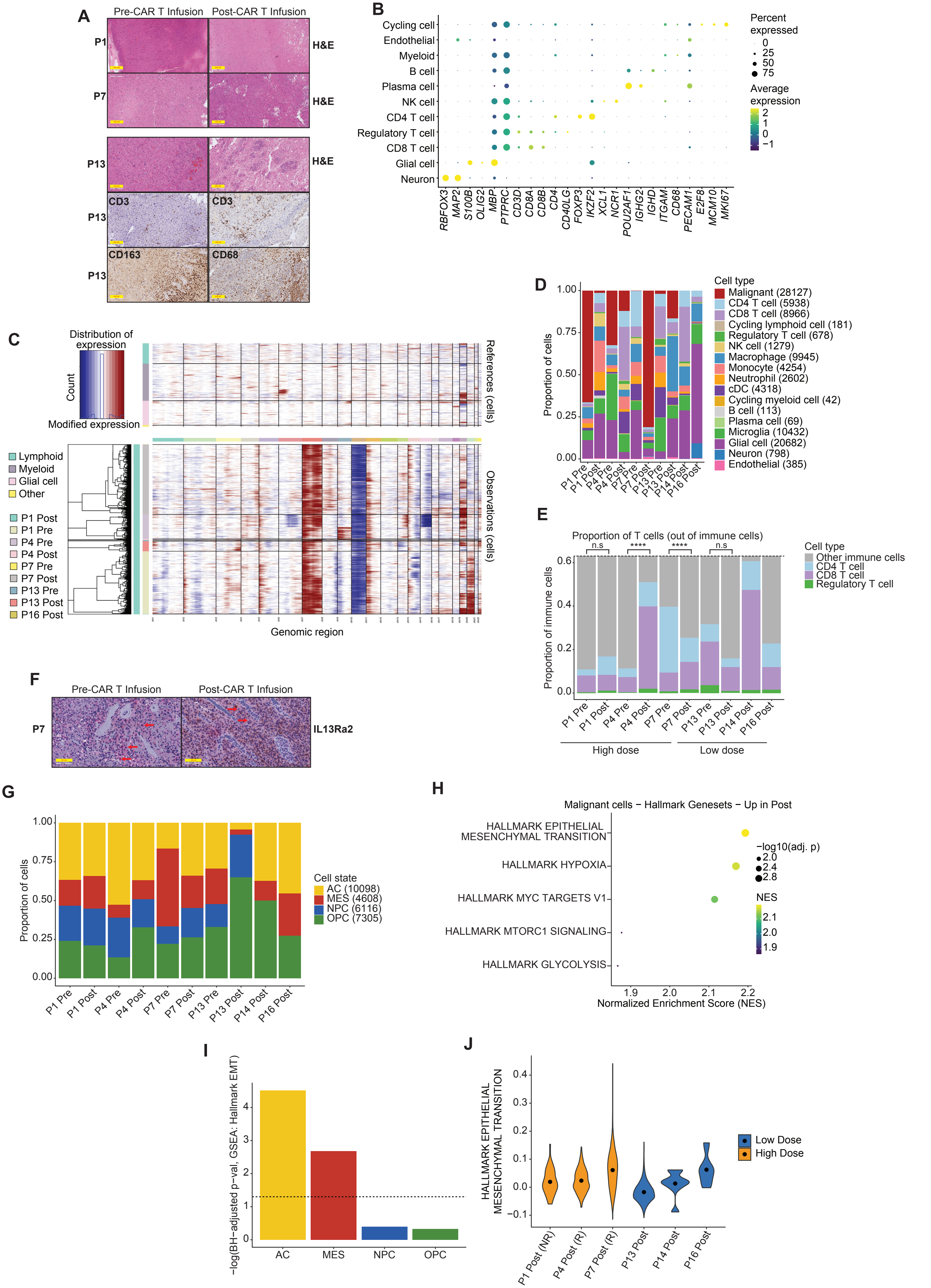

A
Pre-CAR T Infusion
Post-CAR T Infusion
P1
P7
P13
H&E
CD3
CD163
CD68
B
Cycling cell
Endothelial
Myeloid
B cell
Plasma cell
NK cell
CD4 T cell
Regulatory T cell
CD8 T cell
Glial cell
Neuron
RBFOX3 MAP2 S100B OLIG2 MBP PTPRC CD3D CD8A CD8B CD4 CD40LG FOXP3 IKZF2 XCL1 NCR1 POU2AF1 IGHG2 IGHD ITGAM CD68 PECAM1 E2F8 MCM10 MKI67
Percent expressed
0
25
50
75
Average expression
2
1
0
−1
C
Distribution of expression
Count
Modified expression
References (cells)
Observations (cells)
Genomic region
Lymphoid
Myeloid
Glial cell
Other
P1 Post
P1 Pre
P4 Pre
P4 Post
P7 Pre
P7 Post
P13 Pre
P13 Post
P16 Post
D
Proportion of cells
Cell type
Malignant (28127)
CD4 T cell (5938)
CD8 T cell (8966)
Cycling lymphoid cell (181)
Regulatory T cell (678)
NK cell (1279)
Macrophage (9945)
Monocyte (4254)
Neutrophil (2602)
cDC (4318)
Cycling myeloid cell (42)
B cell (113)
Plasma cell (69)
Microglia (10432)
Glial cell (20682)
Neuron (798)
Endothelial (385)
P1 Pre
P1 Post
P4 Pre
P4 Post
P7 Pre
P7 Post
P13 Pre
P13 Post
P14 Post
P16 Post
E
Proportion of T cells (out of immune cells)
Proportion of immune cells
n.s
****
****
n.s
Cell type
Other immune cells
CD4 T cell
CD8 T cell
Regulatory T cell
High dose
Low dose
F
Pre-CAR T Infusion
Post-CAR T Infusion
P7
IL13Ra2
G
Proportion of cells
Cell state
AC (10098)
MES (4608)
NPC (6116)
OPC (7305)
H
Malignant cells − Hallmark Genesets − Up in Post
HALLMARK EPITHELIAL MESENCHYMAL TRANSITION
HALLMARK HYPOXIA
HALLMARK MYC TARGETS V1
HALLMARK MTORC1 SIGNALING
HALLMARK GLYCOLYSIS
−log10(adj. p)
2.0
2.4
2.8
NES
2.1
2.0
1.9
Normalized Enrichment Score (NES)
I
−log(BH-adjusted p-val, GSEA: Hallmark EMT)
AC
MES
NPC
OPC
J
HALLMARK EPITHELIAL MESENCHYMAL TRANSITION
P1 Post (NR)
P4 Post (R)
P7 Post (R)
P13 Post
P14 Post
P16 Post
Low Dose
High Dose

Figure S4. Extending Longitudinal evolution of GBM tumor tissue after intracerebroventricular CAR T cell infusion, related to Figure 3.

(A) H&E or IHC (CD3, CD68, P13 only) tissue sections of pre-CAR T infusion (Left) and post-CAR T infusion (right) tumor tissue from P1, P7, and P13 (top to bottom). Both pre- and post-CAR T infusion tissue showed a mix of tumor and treatment effect. P1 images at 4X, P7, and P13 images at 10X. Scale bars represent 500 μM (P1) and 200 μM (P7, P13).

(B) Average expression (color) and percent of cells (dot size) expressing lineage specific marker genes, used to annotate cell types in single-nucleus RNA-seq data from tumor samples.

(C) Inference of neoplastic cells based on copy number alterations in single-nucleus RNA-seq data. Color reflects inferred copy number alteration (Red higher).

(D) Cell type composition of single-nucleus RNA-seq data (n = 98,809) per sample across pre- and post-treatment tumor samples.

(E) Cell type composition of T cells as a fraction of total immune cells (n = 48817, **** = CD8 T cell composition $p < 0.0001$, one-sided Fisher exact test), in single-nucleus RNA-seq data from each tumor sample.

(F) Expression of IL13Rα2 (pink) by immunohistochemistry in formalin-fixed, paraffin-embedded P7 tumor samples before and after CAR T infusion, with hematoxylin counterstain. Red arrows mark positive cells. Scale bars represent 100 μM.

(G) Proportion of Neftel et al. malignant cell states derived from tumor single-nucleus RNA sequencing.

(H) Top genesets enriched in post-infusion malignant cells from single-nucleus RNA-seq (vs. pre-infusion, MSigDB Hallmark, n = 28,127). GSEA performed with clusterProfiler based on ranked logfold changes from Seurat FindMarkers.

(I) Log-transformed p-value for enrichment of MSigDB HALLMARK_EPITHELIAL_MESENCHYMAL_TRANSITION calculated by GSEA (clusterProfiler) based on ranked log-fold changes from FindMarkers (Seurat). Enrichment calculated separately for each Neftel et al. malignant cell state.

(J) Enrichment of genes in MSigDB HALLMARK_EPITHELIAL_MESENCHYMAL_TRANSITION calculated by AddModuleScore (Seurat) in post-infusion tumor cells (n = 13,423), colored by dose level.

Figure S5

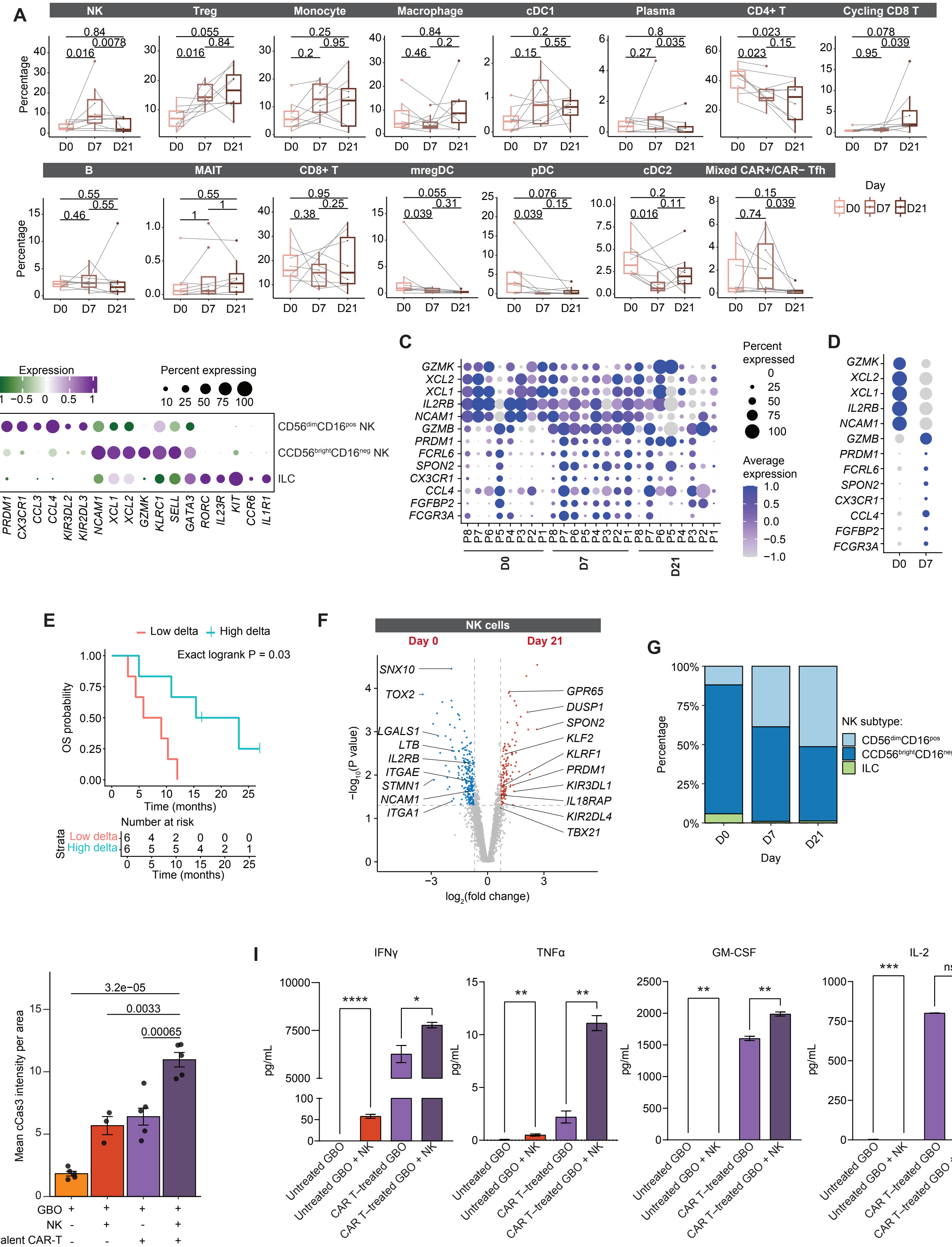
A
NK
Treg
Monocyte
Macrophage
cDC1
Plasma
CD4+ T
Cycling CD8 T
B
MAIT
CD8+ T
mregDC
pDC
cDC2
Mixed CAR+/CAR− Tfh
Percentage
Day
D0
D7
D21
B
Expression
Percent expressing
CD56dimCD16pos NK
CCD56brightCD16neg NK
ILC
C
Percent expressed
Average expression
D
Average expression
Percent expressed
E
Low delta
High delta
Exact logrank P = 0.03
OS probability
Time (months)
Number at risk
Strata
F
NK cells
Day 0
Day 21
log2(fold change)
G
NK subtype:
CD56dimCD16pos
CCD56brightCD16neg
ILC
H
Mean cCas3 intensity per area
GBO
NK
Bivalent CAR-T
I
IFNγ
TNFα
GM-CSF
IL-2
pg/mL
Untreated GBO
Untreated GBO + NK
CAR T–treated GBO
CAR T–treated GBO + NK

Figure S5. Extending phenotypic shift of NK cells associates with improved clinical outcomes, related to Figure 4.

(A) Proportional abundance of non-CAR immune subsets in CSF at D0, D7, and D21. Boxes span the interquartile range (25th - 75th percentiles) with the median shown as a central line; whiskers extend to the most extreme values within 1.5 times IQR (discovery cohort, n = 8 patients). P values from two-sided paired Wilcoxon signed-rank test.

(B) Marker gene expression for $CD56^{bright}CD16^{neg}$, $CD56^{dim}CD16^{pos}$ and ILC populations in the CSF. Dot size indicates the percentage of cells expressing each gene; color represents average expression level.

(C) CSF NK cell phenotypes from D0 to D21 in all patients. Dot color indicates average expression level (scaled), and dot size represents the percentage of NK cells expressing the gene within each sample.

(D) Marker genes for $CD56^{bright}CD16^{neg}$ and $CD56^{dim}CD16^{pos}$ across D0 and D7 in the CSF validation cohort.

(E) Kaplan–Meier curves of overall survival (OS) in patients stratified by the median change in $CD56^{dim}CD16^{pos}$ CSF NK cell fraction (D7-D0, n = 12 patients, High-dose). Tick marks indicate censored observations; numbers at risk are shown below. *P* value from a two-sided exact log-rank test.

(F) Differential gene expression analysis of CSF NK cells at D0 vs D21 (discovery cohort, n = 8 patients). P values from two-sided quasi-likelihood F-test of edgeR. Genes with nominal $p < 0.05$ and absolute fold change > 2 are highlighted. Selected significant DE genes are labeled.

(G) Proportion of NK cell subtypes in CSF at D0, D7, and D21 (discovery cohort, n = 8 patients).

(H) Tumor cell lysis (cleaved caspase-3) in GBO co-cultures batch 2 (n = 6 replicates). Batch 1 is shown in Fig 4L. Mean ± SD, unpaired t-test.

(I) Quantification of inflammatory mediators from the supernatant of Batch 1 GBO-CAR T-NK cell co-culture, shown in Fig 4L. Mean ± SD, unpaired t-test.

Figure S6

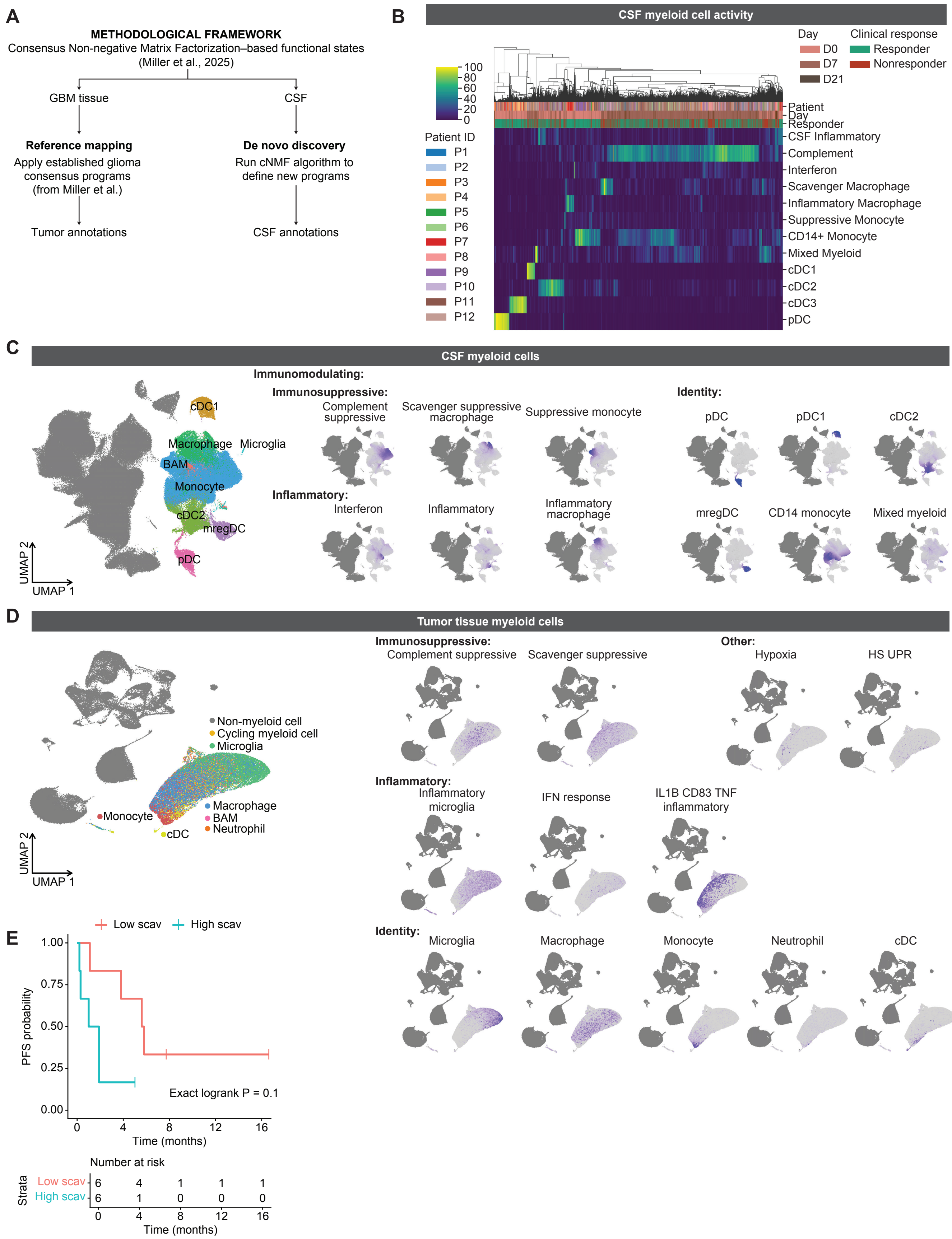

A
METHODOLOGICAL FRAMEWORK
Consensus Non-negative Matrix Factorization–based functional states
(Miller et al., 2025)
GBM tissue
CSF
Reference mapping
Apply established glioma consensus programs (from Miller et al.)
De novo discovery
Run cNMF algorithm to define new programs
Tumor annotations
CSF annotations
B
CSF myeloid cell activity
Day
D0
D7
D21
Clinical response
Responder
Nonresponder
100
80
60
40
20
0
Patient ID
P1
P2
P3
P4
P5
P6
P7
P8
P9
P10
P11
P12
Patient
Day
Responder
CSF Inflammatory
Complement
Interferon
Scavenger Macrophage
Inflammatory Macrophage
Suppressive Monocyte
CD14+ Monocyte
Mixed Myeloid
cDC1
cDC2
cDC3
pDC
C
CSF myeloid cells
cDC1
Macrophage
Microglia
BAM
Monocyte
cDC2
mregDC
pDC
UMAP 2
UMAP 1
Immunomodulating:
Immunosuppressive:
Complement suppressive
Scavenger suppressive macrophage
Suppressive monocyte
Inflammatory:
Interferon
Inflammatory
Inflammatory macrophage
Identity:
pDC
pDC1
cDC2
mregDC
CD14 monocyte
Mixed myeloid
D
Tumor tissue myeloid cells
Non-myeloid cell
Cycling myeloid cell
Microglia
Macrophage
BAM
Neutrophil
Monocyte
cDC
UMAP 2
UMAP 1
Immunosuppressive:
Complement suppressive
Scavenger suppressive
Other:
Hypoxia
HS UPR
Inflammatory:
Inflammatory microglia
IFN response
IL1B CD83 TNF inflammatory
Identity:
Microglia
Macrophage
Monocyte
Neutrophil
cDC
E
Low scav
High scav
PFS probability
1.00
0.75
0.50
0.25
0.00
Exact logrank P = 0.1
0
4
8
12
16
Time (months)
Number at risk
Strata
Low scav
High scav
6 4 1 1 1
6 1 0 0 0
Time (months)

Figure S6. Extending high baseline myeloid immunosuppression associates with poor response, related to Figure 5.

(A) Methodological framework.
(B) Heatmap showing hierarchical clustering of CSF myeloid cells based on cell identity and cell activity program usage. Rows represent usage scores for each program. Columns represent individual cells annotated by patient, day and clinical response. Discovery and validation cohort.
(C and D) UMAP embedding of myeloid cells annotated by their cell types (left) and by their usage of cell identity and cell activity programs (right) in (**C**) CSF (*n* = 74,761 cells, discovery and validation cohort) and (**D**) tumor tissue (*n* = 31,593 cells).
(E) Kaplan–Meier curve of PFS stratified by median baseline Scavenger program usage in CSF (*n* = 12 patients). P value from exact log-rank test.

Figure S7

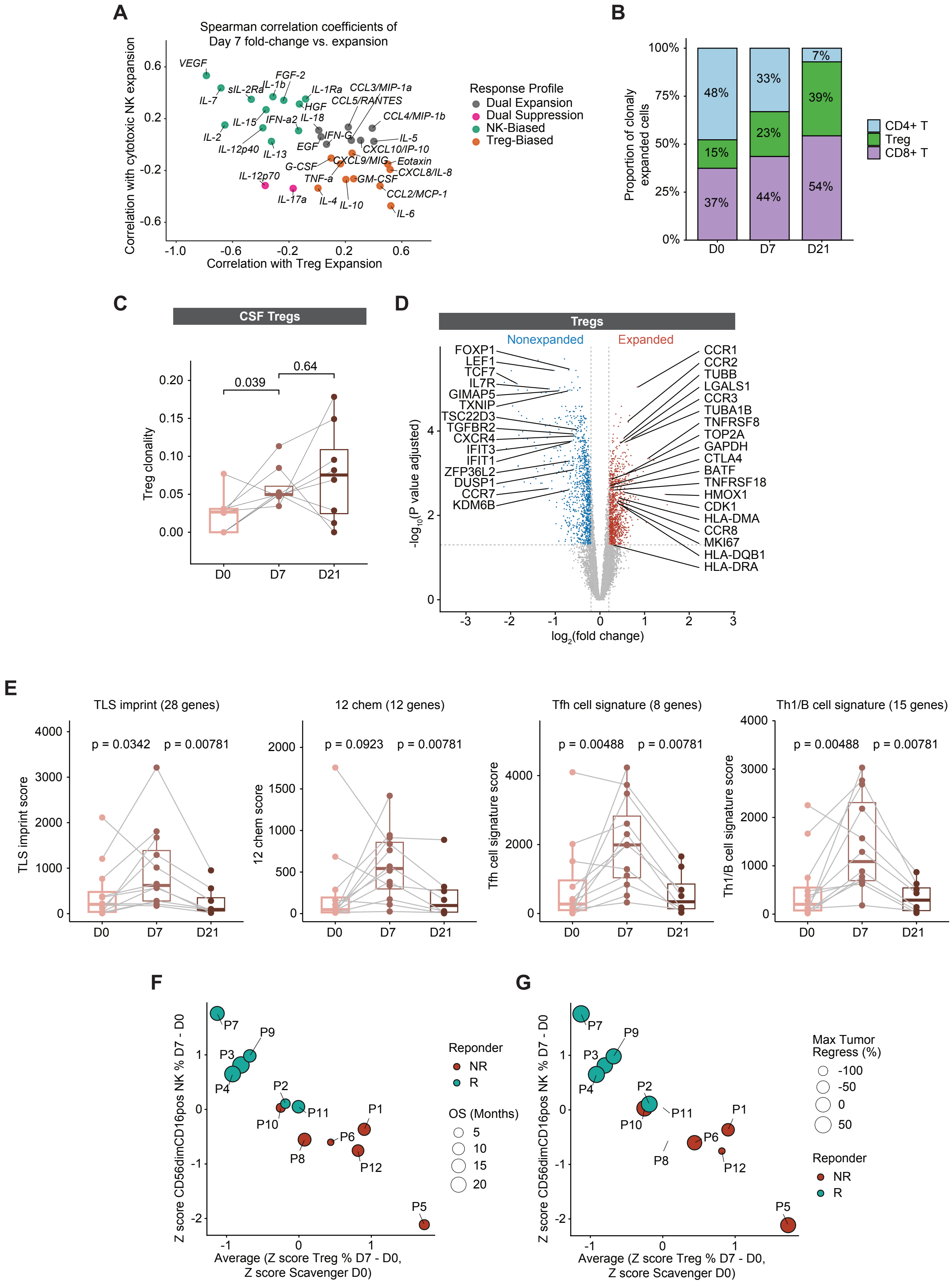
A
Spearman correlation coefficients of
Day 7 fold-change vs. expansion
Correlation with cytotoxic NK expansion
Correlation with Treg Expansion
VEGF
IL-7
sIL-2Ra
IL-1b
FGF-2
IL-1Ra
CCL3/MIP-1a
HGF
CCL5/RANTES
IL-18
CCL4/MIP-1b
IL-15
IFN-a2
IL-2
IL-12p40
IL-13
EGF
IFN-G
IL-5
CXCL10/IP-10
G-CSF
CXCL9/MIG
Eotaxin
CXCL8/IL-8
IL-12p70
TNF-a
GM-CSF
IL-17a
IL-4
IL-10
CCL2/MCP-1
IL-6
Response Profile
Dual Expansion
Dual Suppression
NK-Biased
Treg-Biased
B
Proportion of clonaly expanded cells
48%
15%
37%
33%
23%
44%
7%
39%
54%
D0
D7
D21
CD4+ T
Treg
CD8+ T
C
CSF Tregs
Treg clonality
0.039
0.64
D0
D7
D21
D
Tregs
Nonexpanded
Expanded
FOXP1
LEF1
TCF7
IL7R
GIMAP5
TXNIP
TSC22D3
TGFBR2
CXCR4
IFIT3
IFIT1
ZFP36L2
DUSP1
CCR7
KDM6B
CCR1
CCR2
TUBB
LGALS1
CCR3
TUBA1B
TNFRSF8
TOP2A
GAPDH
CTLA4
BATF
TNFRSF18
HMOX1
CDK1
HLA-DMA
CCR8
MKI67
HLA-DQB1
HLA-DRA
-log10(P value adjusted)
log2(fold change)
E
TLS imprint (28 genes)
p = 0.0342
p = 0.00781
TLS imprint score
12 chem (12 genes)
p = 0.0923
p = 0.00781
12 chem score
Tfh cell signature (8 genes)
p = 0.00488
p = 0.00781
Tfh cell signature score
Th1/B cell signature (15 genes)
p = 0.00488
p = 0.00781
Th1/B cell signature score
F
Z score CD56dimCD16pos NK % D7 - D0
Average (Z score Treg % D7 - D0,
Z score Scavenger D0)
P7
P9
P3
P4
P2
P11
P10
P1
P8
P6
P12
P5
Reponder
NR
R
OS (Months)
5
10
15
20
G
Max Tumor
Regress (%)
-100
-50
0
50
Reponder
NR
R

Figure S7. Extending regulatory T cell expansion inversely correlates with tumor size reduction, related to Figure 6.

(A) Scatter plot of Spearman rank correlation coefficients between log fold increase of individual cytokines at D7 and $CD56^{dim}CD16^{pos}$ NK expansion (y-axis) or non-CAR Treg expansion (x-axis), discovery and validation cohorts. Colors denote profiles defined by correlation direction.

(B) Cell type composition of expanded (>1 cell) non-CAR T cell clones at D0 and D7 in the discovery and validation cohorts combined (*n* = 12 patients; High-dose) and D21 in the discovery cohort alone (*n* = 8 patients).

(C) Change in clonality of the non-CAR Treg TCR repertoire in the discovery cohort (*n* = 8) at D0, D7, and D21.

(D) Differential gene expression analysis comparing clonally expanded (right) and unexpanded (left) non-CAR Tregs at D7. High-dose cohort. P values from two-sided quasi-likelihood F-test of edgeR. Genes with Benjamini-Hochberg-adjusted $p < 0.05$ and absolute fold change > 1.25 are highlighted. Selected significant DE genes are labeled.

(E) Paired box plots of pseudobulk expression scores for four TLS gene signatures in CSF samples at D0, D7, and D21 (D0-D7: n = 12 patients, High-dose, D21: n = 8 patients, discovery cohort) . Scores represent the geometric mean of aggregated single-cell counts across all cell types for genes in each signature. Gray lines connect paired samples from the same patient. *P* values from two-sided paired Wilcoxon signed-rank test.

(F and G) Scatter plots showing the standardized change in $CD56^{dim}CD16^{pos}$ NK cell proportion (D7 - D0, y-axis) versus a composite immunosuppressive metric (mean Z-score of D7 - D0 non-CAR Treg proportion and baseline Scavenger Macrophages, x-axis) (n = 12 patients, High-dose. Patients are colored by response. Point size represents (**F**) overall survival and (**G**) tumor size reduction.

Supplemental Table 2. Per patient comparison of cytotoxicity and exhaustion signature scores between CAR and non-CAR T cells.

Related to Figure 2.

Patient	Feature	p-value	Mean (Non-CAR T)	Mean (CAR T)	Direction of change	Adj. p-val	Cohort
P1	Cytotoxicity	2.22E-16	0.161	0.191	CART>Non-CART	2.66E-16	discovery
P2	Cytotoxicity	2.22E-16	0.181	0.223	CART>Non-CART	2.66E-16	discovery
P3	Cytotoxicity	0.00028356	0.185	0.198	CART>Non-CART	0.00030934	discovery
P4	Cytotoxicity	2.22E-16	0.174	0.225	CART>Non-CART	2.66E-16	discovery
P5	Cytotoxicity	2.22E-16	0.164	0.209	CART>Non-CART	2.66E-16	discovery
P6	Cytotoxicity	2.22E-16	0.175	0.199	CART>Non-CART	2.66E-16	discovery
P7	Cytotoxicity	2.22E-16	0.168	0.209	CART>Non-CART	2.66E-16	discovery
P8	Cytotoxicity	2.22E-16	0.162	0.229	CART>Non-CART	2.66E-16	discovery
P9	Cytotoxicity	2.22E-16	0.167	0.234	CART>Non-CART	2.66E-16	validation
P10	Cytotoxicity	2.22E-16	0.164	0.252	CART>Non-CART	2.66E-16	validation
P11	Cytotoxicity	2.22E-16	0.191	0.212	CART>Non-CART	2.66E-16	validation
P12	Cytotoxicity	2.22E-16	0.161	0.201	CART>Non-CART	2.66E-16	validation
P1	Exhaustion	2.22E-16	0.146	0.224	CART>Non-CART	2.66E-16	discovery
P2	Exhaustion	2.22E-16	0.149	0.239	CART>Non-CART	2.66E-16	discovery
P3	Exhaustion	2.22E-16	0.155	0.236	CART>Non-CART	2.66E-16	discovery
P4	Exhaustion	2.22E-16	0.144	0.217	CART>Non-CART	2.66E-16	discovery
P5	Exhaustion	2.22E-16	0.152	0.218	CART>Non-CART	2.66E-16	discovery
P6	Exhaustion	2.22E-16	0.155	0.227	CART>Non-CART	2.66E-16	discovery
P7	Exhaustion	2.22E-16	0.138	0.236	CART>Non-CART	2.66E-16	discovery
P8	Exhaustion	2.22E-16	0.136	0.243	CART>Non-CART	2.66E-16	discovery
P9	Exhaustion	2.22E-16	0.148	0.236	CART>Non-CART	2.66E-16	validation
P10	Exhaustion	2.22E-16	0.124	0.258	CART>Non-CART	2.66E-16	validation
P11	Exhaustion	2.22E-16	0.146	0.219	CART>Non-CART	2.66E-16	validation
P12	Exhaustion	2.22E-16	0.148	0.226	CART>Non-CART	2.66E-16	validation

Supplemental Table 3. Per patient differential expression analysis of selected genes in CAR vs. non-CAR T cells.

Related to Figure 2.

Patient	gene	mean_CARneg	mean_CARpos	diff	p_value	direction	p.adj
P1	CD38	0.391	1.228	0.837	2.22E-16	higher in CAR+	2.96E-16
P1	GNLY	0.299	1.777	1.478	2.22E-16	higher in CAR+	2.96E-16
P1	HAVCR2	0.466	0.849	0.383	2.22E-16	higher in CAR+	2.96E-16
P1	HLA-DRA	0.479	1.516	1.037	2.22E-16	higher in CAR+	2.96E-16
P1	IFI6	0.36	0.427	0.067	2.22E-16	higher in CAR+	2.96E-16
P1	LAG3	0.724	0.638	-0.086	0.02440323	higher in CAR-	0.02603011
P1	PRF1	1.169	0.996	-0.173	0.00013589	higher in CAR-	0.00015812
P1	STAT1	0.794	1.336	0.542	2.22E-16	higher in CAR+	2.96E-16
P2	CD38	0.18	0.763	0.583	2.22E-16	higher in CAR+	2.96E-16
P2	GNLY	0.969	1.621	0.652	2.22E-16	higher in CAR+	2.96E-16
P2	HAVCR2	0.187	0.903	0.716	2.22E-16	higher in CAR+	2.96E-16
P2	HLA-DRA	0.272	1.371	1.099	2.22E-16	higher in CAR+	2.96E-16
P2	IFI6	0.286	0.787	0.501	2.22E-16	higher in CAR+	2.96E-16
P2	LAG3	0.555	0.767	0.212	2.22E-16	higher in CAR+	2.96E-16
P2	PRF1	1.128	1.146	0.018	0.95423389	higher in CAR+	0.96938046
P2	STAT1	0.583	1.434	0.851	2.22E-16	higher in CAR+	2.96E-16
P3	CD38	0.239	0.752	0.513	2.22E-16	higher in CAR+	2.96E-16
P3	GNLY	0.504	1.164	0.66	1.72E-10	higher in CAR+	2.12E-10
P3	HAVCR2	0.168	0.926	0.758	2.22E-16	higher in CAR+	2.96E-16
P3	HLA-DRA	0.35	0.795	0.445	1.68E-14	higher in CAR+	2.20E-14
P3	IFI6	0.26	0.478	0.218	4.21E-11	higher in CAR+	5.29E-11
P3	LAG3	0.516	0.599	0.083	0.02177852	higher in CAR+	0.02362415
P3	PRF1	1.15	1.168	0.018	0.97071239	higher in CAR+	0.97071239
P3	STAT1	0.505	0.86	0.355	3.41E-11	higher in CAR+	4.37E-11
P4	CD38	0.267	1.213	0.946	2.22E-16	higher in CAR+	2.96E-16
P4	GNLY	0.76	2.454	1.694	2.22E-16	higher in CAR+	2.96E-16
P4	HAVCR2	0.131	0.757	0.626	2.22E-16	higher in CAR+	2.96E-16
P4	HLA-DRA	0.291	1.131	0.84	2.22E-16	higher in CAR+	2.96E-16
P4	IFI6	0.25	0.692	0.442	2.22E-16	higher in CAR+	2.96E-16

P4	LAG3	0.456	0.9	0.444	2.22E-16	higher in CAR+	2.96E-16
P4	PRF1	1.064	1.182	0.118	0.00021618	higher in CAR+	0.00024706
P4	STAT1	0.973	1.528	0.555	2.22E-16	higher in CAR+	2.96E-16
P5	CD38	0.123	0.697	0.574	2.22E-16	higher in CAR+	2.96E-16
P5	GNLY	0.678	2.711	2.033	2.22E-16	higher in CAR+	2.96E-16
P5	HAVCR2	0.182	0.702	0.52	2.22E-16	higher in CAR+	2.96E-16
P5	HLA-DRA	0.247	0.992	0.745	2.22E-16	higher in CAR+	2.96E-16
P5	IFI6	0.265	0.585	0.32	2.22E-16	higher in CAR+	2.96E-16
P5	LAG3	0.61	0.67	0.06	0.06119173	higher in CAR+	0.06420116
P5	PRF1	1.055	1.152	0.097	0.12099521	higher in CAR+	0.12489828
P5	STAT1	0.805	1.034	0.229	4.72E-06	higher in CAR+	5.59E-06
P6	CD38	0.117	0.934	0.817	2.22E-16	higher in CAR+	2.96E-16
P6	GNLY	0.745	1.461	0.716	2.22E-16	higher in CAR+	2.96E-16
P6	HAVCR2	0.088	0.67	0.582	2.22E-16	higher in CAR+	2.96E-16
P6	HLA-DRA	0.268	1.143	0.875	2.22E-16	higher in CAR+	2.96E-16
P6	IFI6	0.158	0.513	0.355	2.22E-16	higher in CAR+	2.96E-16
P6	LAG3	0.581	0.621	0.04	0.01455175	higher in CAR+	0.0163388
P6	PRF1	1.135	1.086	-0.049	0.02121521	higher in CAR-	0.02340989
P6	STAT1	0.563	1.069	0.506	2.22E-16	higher in CAR+	2.96E-16
P7	CD38	0.142	0.785	0.643	2.22E-16	higher in CAR+	2.96E-16
P7	GNLY	0.382	1.747	1.365	2.22E-16	higher in CAR+	2.96E-16
P7	HAVCR2	0.085	0.846	0.761	2.22E-16	higher in CAR+	2.96E-16
P7	HLA-DRA	0.298	1.185	0.887	2.22E-16	higher in CAR+	2.96E-16
P7	IFI6	0.211	0.561	0.35	2.22E-16	higher in CAR+	2.96E-16
P7	LAG3	0.437	0.709	0.272	2.22E-16	higher in CAR+	2.96E-16
P7	PRF1	0.917	1.169	0.252	2.22E-16	higher in CAR+	2.96E-16
P7	STAT1	0.994	1.332	0.338	2.22E-16	higher in CAR+	2.96E-16
P8	CD38	0.302	1.043	0.741	2.22E-16	higher in CAR+	2.96E-16
P8	GNLY	0.208	2.348	2.14	2.22E-16	higher in CAR+	2.96E-16
P8	HAVCR2	0.173	1.079	0.906	2.22E-16	higher in CAR+	2.96E-16
P8	HLA-DRA	0.23	1.2	0.97	2.22E-16	higher in CAR+	2.96E-16
P8	IFI6	0.51	0.79	0.28	2.22E-16	higher in CAR+	2.96E-16
P8	LAG3	0.592	1.042	0.45	2.22E-16	higher in CAR+	2.96E-16
P8	PRF1	0.945	1.572	0.627	2.22E-16	higher in CAR+	2.96E-16

P8	STAT1	1.309	1.534	0.225	2.60E-08	higher in CAR+	3.13E-08
P9	CD38	0.193	1.034	0.841	2.22E-16	higher in CAR+	2.84E-16
P9	GNLY	0.262	0.926	0.664	5.53E-11	higher in CAR+	6.56E-11
P9	HAVCR2	0.263	1.005	0.742	2.22E-16	higher in CAR+	2.84E-16
P9	HLA-DRA	0.234	1.485	1.251	2.22E-16	higher in CAR+	2.84E-16
P9	IFI6	0.562	1.623	1.061	2.22E-16	higher in CAR+	2.84E-16
P9	LAG3	0.508	1.073	0.565	2.22E-16	higher in CAR+	2.84E-16
P9	PRF1	0.957	1.258	0.301	5.86E-06	higher in CAR+	6.25E-06
P9	STAT1	0.797	1.605	0.808	2.22E-16	higher in CAR+	2.84E-16
P10	CD38	0.189	1.415	1.226	2.22E-16	higher in CAR+	2.84E-16
P10	GNLY	0.427	1.721	1.294	2.22E-16	higher in CAR+	2.84E-16
P10	HAVCR2	0.013	1.094	1.081	2.22E-16	higher in CAR+	2.84E-16
P10	HLA-DRA	0.24	1.238	0.998	2.22E-16	higher in CAR+	2.84E-16
P10	IFI6	0.731	1.345	0.614	2.22E-16	higher in CAR+	2.84E-16
P10	LAG3	0.577	1.341	0.764	2.22E-16	higher in CAR+	2.84E-16
P10	PRF1	0.815	1.731	0.916	2.22E-16	higher in CAR+	2.84E-16
P10	STAT1	1.681	1.984	0.303	4.77E-12	higher in CAR+	5.87E-12
P11	CD38	0.087	1.134	1.047	2.22E-16	higher in CAR+	2.84E-16
P11	GNLY	1.307	1.971	0.664	2.22E-16	higher in CAR+	2.84E-16
P11	HAVCR2	0.008	0.697	0.689	2.22E-16	higher in CAR+	2.84E-16
P11	HLA-DRA	0.215	0.736	0.521	2.22E-16	higher in CAR+	2.84E-16
P11	IFI6	0.201	0.717	0.516	2.22E-16	higher in CAR+	2.84E-16
P11	LAG3	0.55	0.617	0.067	1.25E-06	higher in CAR+	1.37E-06
P11	PRF1	1.214	1.127	-0.087	6.81E-05	higher in CAR-	7.03E-05
P11	STAT1	0.975	1.332	0.357	2.22E-16	higher in CAR+	2.84E-16
P12	CD38	0.259	1.224	0.965	2.22E-16	higher in CAR+	2.84E-16
P12	GNLY	1.216	3.074	1.858	2.22E-16	higher in CAR+	2.84E-16
P12	HAVCR2	0.049	1.089	1.04	2.22E-16	higher in CAR+	2.84E-16
P12	HLA-DRA	0.072	0.659	0.587	2.22E-16	higher in CAR+	2.84E-16
P12	IFI6	0.121	0.821	0.7	2.22E-16	higher in CAR+	2.84E-16
P12	LAG3	0.634	1.004	0.37	2.30E-07	higher in CAR+	2.63E-07
P12	PRF1	1.096	1.298	0.202	0.04785647	higher in CAR+	0.04785647
P12	STAT1	0.609	1.538	0.929	2.22E-16	higher in CAR+	2.84E-16

Supplemental Table 4. Quantification of immunohistochemistry staining for tumor-infiltrating immune cells.

Related to Figure 3, Figure S4.

	P1		P4		P7	
Patient	Pre-Infusion	Post-Infusion	Pre-Infusion	Post-Infusion	Pre-Infusion	Post-Infusion
Macrophages/microglia in tumor	3	n/a	4	4	3	2
Macrophages/microglia in treatment	n/a	4	3	n/a	4	4
CD3 in tumor	4	n/a	1	3	2	2
CD3 in treatment	n/a	2	2	n/a	4	3
Amount of tumor	high	minimal	high	high	minimal	high

	P13		P14		P16	
Patient	Pre-Infusion	Post-Infusion	Pre-Infusion	Post-Infusion	Pre-Infusion	Post-Infusion
Macrophages/microglia in tumor	4	5	n/a	n/a	n/a	3
Macrophages/microglia in treatment	2	6	3	5	4	4
CD3 in tumor	4	2	n/a	n/a	n/a	4
CD3 in treatment	n/a	6	3	5	3	2
Amount of tumor	high	focal	minimal	minimal	minimal	minimal

Supplemental Table 6. Quality and filtering metrics for CSF, tumor samples, and infusion product. Related to Figure 1, Figure 2, Figure 3.

sample	tissue_type	Average RNA counts per cell post filtering	Average genes detected per cell post filtering	Total cells post filtering	Fraction of cells manually filtered after CellRanger
P1D0	CSF	12268.13	3266.40	1089	0.61
P1D7	CSF	21503.61	4589.13	5692	0.50
P1D21	CSF	4220.02	1833.76	1635	0.50
P2D0	CSF	13765.71	3406.60	359	0.76
P2D7	CSF	18517.03	4404.65	6828	0.47
P2D21	CSF	10812.83	2900.20	769	0.46
P3D0	CSF	18043.17	4094.80	572	0.54
P3D7	CSF	17756.83	4364.90	1275	0.40
P3D21	CSF	10041.79	2880.16	463	0.36
P4D0	CSF	13825.02	3538.15	5472	0.43
P4D7	CSF	16089.43	4123.77	10881	0.29
P4D21	CSF	3514.64	1557.25	1633	0.56
P5D0	CSF	12984.47	3275.60	533	0.39
P5D7	CSF	9995.60	3189.37	3100	0.26
P5D21	CSF	14109.29	3641.70	428	0.51
P6D0	CSF	13403.68	3662.66	3070	0.38
P6D7	CSF	18196.29	4393.91	4672	0.30
P6D21	CSF	8258.72	2738.21	135	0.19
P7D0	CSF	12032.79	3402.55	9777	0.36
P7D7	CSF	19260.59	4562.61	9145	0.41
P7D21	CSF	10550.63	3263.02	4602	0.35
P8D0	CSF	14341.08	3823.66	741	0.49
P8D7	CSF	14403.21	3940.79	9367	0.43
P8D21	CSF	12849.90	3616.12	3571	0.48
P9D0	CSF	13036.82	3355.69	2358	0.31
P9D7	CSF	16063.47	3875.21	3906	0.33
P10D0	CSF	16971.73	4107.17	306	0.59
P10D7	CSF	15763.24	3778.04	15416	0.26
P11D0	CSF	11747.00	3154.26	5077	0.28
P11D7	CSF	14537.26	3682.96	11350	0.23
P12D0	CSF	22753.08	5094.33	64	0.67
P12D7	CSF	18543.13	4402.26	3672	0.34
P13D0	CSF	16984.90	3983.18	18337	0.27
P13D7	CSF	12040.49	3016.52	20488	0.27
P14D0	CSF	13826.48	3782.34	14675	0.21

P14D7	CSF	10543.73	2905.33	4472	0.27
P15D0	CSF	11899.68	3019.87	2924	0.20
P15D7	CSF	15235.16	3599.26	13460	0.26
P16D0	CSF	13522.22	3287.30	1257	0.34
P16D7	CSF	15277.91	3823.48	16180	0.24
P17D0	CSF	10799.33	2616.88	1256	0.35
P17D7	CSF	13257.49	3060.80	8171	0.27
P18D0	CSF	11896.43	2867.55	837	0.25
P18D7	CSF	15809.39	3480.67	18819	0.23
P1IP	IP	23962.22	5043.27	9060	0.27
P2IP	IP	21578.92	4649.44	13847	0.19
P3IP	IP	27492.66	5704.97	6782	0.39
P4IP	IP	27495.73	5299.82	5661	0.46
P5IP	IP	20946.31	4604.79	9978	0.42
P6IP	IP	25348.03	5078.11	3993	0.35
P7IP	IP	20718.49	4498.82	10008	0.23
P8IP	IP	7519.82	2603.68	20324	0.56
P1 Pre	Tumor	14200.16	3795.31	15804	0.32
P1 Post	Tumor	13748.17	3159.86	2605	0.17
P4 Pre	Tumor	11217.23	3435.49	12345	0.13
P4 Post	Tumor	14864.89	3341.88	1554	0.17
P7 Pre	Tumor	9483.46	2979.27	12826	0.28
P7 Post	Tumor	10762.09	3529.65	14133	0.22
P13 Pre	Tumor	8126.43	2670.05	8663	0.30
P13 Post	Tumor	7946.55	2630.15	10278	0.26
P14 Post	Tumor	6602.21	2338.20	12620	0.20
P16 Post	Tumor	16252.77	3951.17	7981	0.19
